# Identification and Characterization of Glycoproteins and Their Responsive Patterns upon Ethylene Stimulation in the Rubber Latex

**DOI:** 10.3390/ijms21155282

**Published:** 2020-07-25

**Authors:** Li Yu, Boxuan Yuan, Lingling Wang, Yong Sun, Guohua Ding, Ousmane Ahmat Souleymane, Xueyan Zhang, Quanliang Xie, Xuchu Wang

**Affiliations:** 1 College of Life Sciences, Shihezi University, Shihezi 832003, China; yulixjnu@163.com (L.Y.); yuanboxuan111@163.com (B.Y.); 2 Key Laboratory for Ecology of Tropical Islands, Ministry of Education, College of Life Sciences, Hainan Normal University, Haikou 571158, China; wll_198927@126.com (L.W.); dingguohuasw@163.com (G.D.); ousmaneben062@gmail.com (O.A.S.); zhangxueyan_caas@126.com (X.Z.); 3 Rubber Research Institute, Chinese Academy of Tropical Agricultural Sciences, Danzhou 571737, China; sunyong_03119308@126.com

**Keywords:** comparative proteomics, *Hevea brasiliensis*, Hydroxymethylglutaryl-CoA synthase, glycosylated proteins, natural rubber biosynthesis, rubber latex

## Abstract

Natural rubber is an important industrial material, which is obtained from the only commercially cultivated rubber tree, *Hevea brasiliensis*. In rubber latex production, ethylene has been extensively used as a stimulant. Recent research showed that post-translational modifications (PTMs) of latex proteins, such as phosphorylation, glycosylation and ubiquitination, are crucial in natural rubber biosynthesis. In this study, comparative proteomics was performed to identify the glycosylated proteins in rubber latex treated with ethylene for different days. Combined with Pro-Q Glycoprotein gel staining and mass spectrometry techniques, we provided the first visual profiling of glycoproteomics of rubber latex and finally identified 144 glycosylated protein species, including 65 differentially accumulated proteins (DAPs) after treating with ethylene for three and/or five days. Gene Ontology (GO) functional annotation showed that these ethylene-responsive glycoproteins are mainly involved in cell parts, membrane components and metabolism. Pathway analysis demonstrated that these glycosylated rubber latex proteins are mainly involved in carbohydrate metabolism, energy metabolism, degradation function and cellular processes in rubber latex metabolism. Protein–protein interaction analysis revealed that these DAPs are mainly centered on acetyl-CoA acetyltransferase and hydroxymethylglutaryl-CoA synthase (HMGS) in the mevalonate pathway for natural rubber biosynthesis. In our glycoproteomics, three protein isoforms of HMGS2 were identified from rubber latex, and only one HMGS2 isoform was sharply increased in rubber latex by ethylene treatment for five days. Furthermore, the *HbHMGS2* gene was over-expressed in a model rubber-producing grass *Taraxacum Kok-saghyz* and rubber content in the roots of transgenic rubber grass was significantly increased over that in the wild type plant, indicating HMGS2 is the key component for natural rubber production.

## 1. Introduction

Natural rubber (NR), as a kind of high-molecular polymer with unique properties, is produced from laticifer cells in the bark of the rubber tree (*Hevea brasiliensis*), which is the only tree species that is widely planted as a commercially available source of NR used for many products, varying from tires to medical products [1]. In NR production, rubber latex is usually obtained by regular tapping of the trunk bark with a two- or three-day interval with the application of ethylene as a stimulant [2]. As one of the most important phytohormones, ethylene has been widely used to accelerate the processes of plant growth and development, including the production of NR [3]. When applying or spraying the ethylene generator, known as ethephon or ethrel (chloro-2-ethyl phosphonic acid), on the trunk bark of the tapped rubber tree, both the fresh latex production and latex regeneration between different tapping events can be significantly increased [4]. The ^14^C-labeled experiment demonstrated that ethylene was released from the treated bark to the upper leaf shortly after supplying with ethephon. This stimulation effect is associated with marked changes in both physiology and metabolism in laticifer cells [5,6]. Our previous results also demonstrated that ethylene stimulation can sharply improve the yield of fresh latex and dry matter, prolonging the latex flow time [7], as well as markedly accelerating the generation of small rubber particles (SRPs) in rubber latex [8].

However, it is still a mystery that, although the ethephon-stimulated rubber production has been widely used for several decades in commercial NR production, many genes involved in NR biosynthesis (NRB) were found to be significantly inhibited after ethylene application [4,9]. Our recently published results also proved that the expression levels of the 27 selected genes were inhibited upon ethylene treatment. The other 25 transcripts either did not change or changed less than the level of their encoded proteins. Among the down-regulated ones, the genes encoding several enzymes known to be the key factors in rubber biosynthesis have been determined [5]. More recently published results also revealed that proteins might be the key regulators for NRB [10,11,12,13,14], and post-translational modifications (PTMs) of different protein isoforms may play crucial roles in NRB upon ethylene treatment [7,15,16], especially in small rubber particles [8]. There are more than three hundred kinds of reported PTMs in proteins [17]. Among them, phosphorylation, glycosylation and ubiquitination are considered as the most three important kinds of PTMs in controlling the final function of enzymes involved in NRB [7,8,14]. Our proteomics results have demonstrated that isoform-specific phosphorylation of proteins in rubber particles (RPs) is important for ethylene-stimulated latex production [7,8].

Almost all proteins can produce different kinds of PTMs, and these PTMs are important for responding to the external environmental changes in different plant cells [18,19]. As one of the most important PTMs, protein glycosylation is an essential co- and post-translational modification in secretory and membrane proteins in many eukaryotes, and it is widely observed in many plant species [20]. Recently, the roles of protein glycosylation have received more considerable attention by researchers for their regulation mechanism of the cell biology field in eukaryotic cells [19,20]. In many plants, it was reported that protein glycosylation can control tissue and organ formation, signal recognition, protein activity promotion, protein structure and protein stability [18,19,20]. Protein-glycosylated modifications result from adding sugar chains to the amino acid residues of proteins and maintain the folding and stability of proteins to perform their biological functions [21]. Two different kinds of sugar chains, named O- and N-sugar chains, are usually examined in glycosylated proteins. Among them, O-glycosylation is a common PTM in many proteins, and these O-sugar chains have different roles in development, cell differentiation, pathogenesis and proteolysis in plant cells [18]. On the other side, N-sugar chains can mainly affect the folding and structure of proteins, and thus are traditionally found to regulate the subcellular localization and protein secretion in different plant cells [21,22]. It was reported that N-sugar chains play regulatory roles in both the plant–pathogen interaction [23] and receptor recognition processes to mediate plant immunity [24].

With the development of proteomics, different kinds of technologies have been used to identify, enrich and separate the glycoproteins. Among them, concanavalin A lectin affinity chromatography has been widely used to separate glycoproteins. With mass spectrometry technology, Catala and coworkers determined many glycoproteins involved in the secretion pathway in ripe tomato fruit [25]. Meanwhile, this technique had also been used to enrich the glycoproteins in the xylem juice of cabbage, and the results demonstrated that most of the enriched proteins are involved in regulating the secretion process [26]. Besides, proteomics analyses also revealed that glycosylated proteins might play important roles in plant defense against fungal invasion [27] and the course of transforming enzymes in white wine as sweet molecular renaissance [28]. Another classic method for detecting glycoproteins is Pro-Q Emerald 488 glycoprotein stain, which can provide direct detection of glycoproteins in gels and on blots rapidly and sensitively. It reacts with periodate-oxidized carbohydrate groups, creating a bright green fluorescent signal on glycoproteins [29]. This stain has been widely used in detecting glycoproteins from human idiopathic pulmonary fibrosis [30], human total serum and mouse liver [31] and skeletal muscle [32]. These above results indicate that the proteomic technique is of great significance for the identification of glycoproteins and the study of plant glycoproteins in pathological regulation and protein catalytic mechanisms.

In this study, we used gel-based experiments to determine the glycoproteins in the total proteins from the collected rubber latex after ethylene stimulation and identified more than one hundred glycoproteins in the stained gel spots with mass spectrometry. To our best knowledge, this is the first proteomics report on ethylene-stimulated glycosylation proteins, which may provide new insights for revealing the regulation mechanism of NRB from the aspects of glycosylation proteins in the rubber latex.

## 2. Results

### 2.1. Profiles of the Glycosylated Proteins from Rubber Latex in One-Dimensional Gel

**Figure 1:**
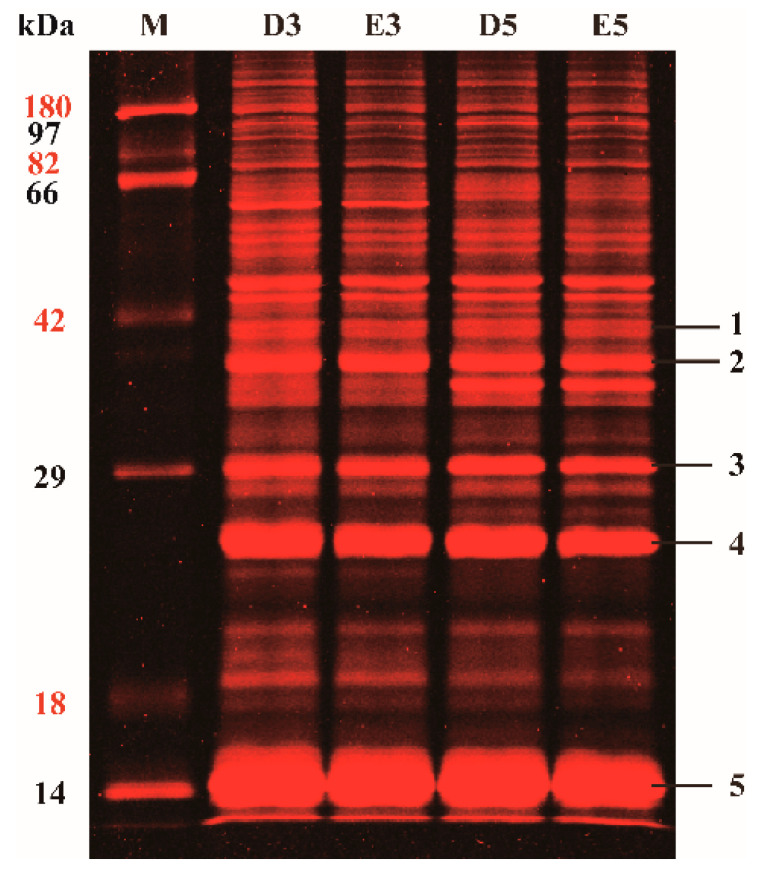
Profiles of glycosylated proteins from the rubber latex in 1-DE gel. Total proteins were extracted from the rubber latex after treating with water and ethylene for three (D3; E3) and five (D5; E5) days, and then the glycosylated protein bands were visualized by Pro-Q Emerald 488 stain. The main bands in the gels were exposed to MALDI TO/TOF MS and five main protein bands were positively identified from these bands. M, protein markers.

Pro-Q Emerald 488 staining is a sensitive method to detect glycoproteins in both gels and blots. In this study, total proteins from rubber latex treated with double-distilled water (ddH_2_O) and ethylene for three days (D3, E3) and five days (D5, E5) were respectively extracted to perform one-dimensional electrophoresis (1-DE) analysis and the glycosylated proteins were then stained by using Pro-Q Emerald 488 stain to a red color. More than thirty red bands, ranging from 14 to 180 kDa, were detected in the lines of each 1-DE gel (Figure 1), indicating that most proteins were glycosylated in the rubber latex. Although several differential bands were observed, the main red bands showed similar change patterns after different time length treatments. We excised the most abundant bands from the 1-DE gel and then performed in-gel digestion, and finally identified five proteins by matrix-assisted laser desorption ionization tandem time-of-flight (MALDI TOF/TOF) mass spectrometry (MS). The five protein bands were identified as β-1,3-glucanse (bands 1 and 2), small rubber particle protein (SRPP, band 3) and rubber elongation factor (REF, bands 4 and 5). Among them, the most abundant bands were REF, which were respectively approximately 14 and 23 kDa.

The changed patterns of the protein bands in the 1-DE gels for protein samples from different water and ethylene treatments were compared, and the protein abundance of the bands was found to vary with different treated time points. That means more obviously changed bands can be detected from the latex samples collected from different days. Several main bands with high molecular weights (approximately 60 kDa) could only be detected in the samples treated for three days (D3 and E3) but disappeared after the five-day treatment. On the other hand, several abundant bands with approximately 40 kDa could only be observed in the five-day treatment (D5 and E5) latex samples. These results indicated that the changes in protein abundance between different time points were more obvious than that from different ethylene treatments. Therefore, we divided three-day and five-day treatments as the two groups and paid more attention to the differential proteins that were collected from the same time point with different treatments in the following study.

### 2.2. Profiles of Glycosylated Proteins Abundance in the Reference 2-DE Proteome Map for the Rubber Latex

**Figure 2:**
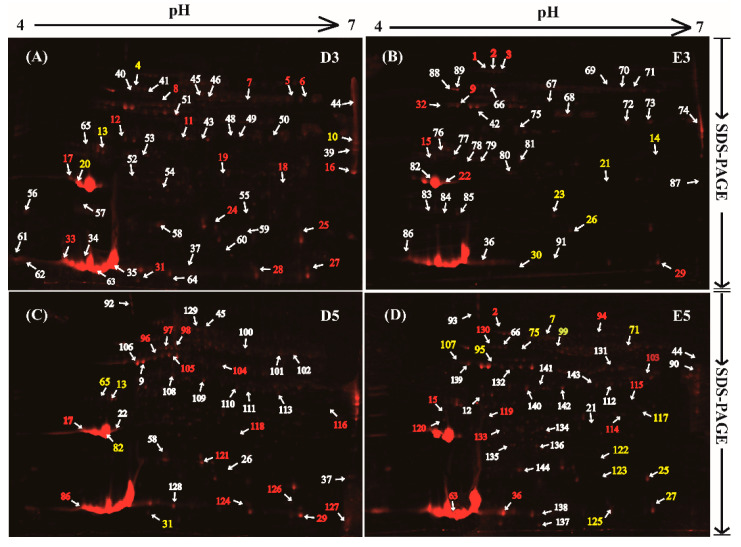
Reference proteome profiles of the glycosylated proteins in 2-DE gels and MS identification of differentially accumulated glycoproteins after ethylene treatments. The glycosylated proteins in total rubber latex from the rubber tree treated with water (**A**,**C**) and ethylene (**B**,**D**) for three (D3, E3) and five days (D5, E5) were dyed with Pro-Q Emerald 488 in the 2-DE gels and the protein spots were visualized by a typhoon scanner. The main protein spots were incised, and 144 protein spots were positively identified by MS. The ethylene-induced protein spots are marked with red numbers, and reduced spots are yellow numbers. The white numbers stand for the unchanged protein spots upon stimulation. These typical 2-DE gels were selected from the three biological replicates. Both protein accumulation levels and the fold change patterns are the average value from the three replicates. The glycosylated protein identities are presented in Table S1 and Figure S1.

Our results showed that many protein bands in the 1-DE gel (Figure 1) and protein spots in the 2-DE gels (Figure 2) could be dyed to a red color, indicating that these proteins are likely to be glycosylated in total rubber latex. The protein spots with an abundance value (Vol%) > 0.02 in the 2-DE gels of different treatments were selected and used to perform MS, and finally, 144 protein spots were successfully identified from the 2-DE gels for latex proteins from different treatments (Figure 2). These 144 proteins are encoded by 98 genes (Table S1 and Figure S1). The highly abundant proteins identified in this study are REF (spots 34, 35, 63 and 82), SRPP (spot 20), anhydrase (spots 10), protease (spot 74), triosephosphate isomerase (spot 61), enolase (spot51), cis-trans isomerase (spot 106), chitinase (spot 9) and a REF/SRPP-like protein (spot 39). Among them, the most abundant protein spot is REF (spot 35), followed by SRPP (spot 82) and anhydrase (spot 74).

The Kyoto Encyclopedia of Genes and Genomes (KEGG) pathway and Gene Ontology (GO) function analyses were performed to determine the potential metabolism processes and biological functions for the identified 98 unique glycoproteins. KEGG results exhibited that these proteins are involved in 32 global and overview maps. Among them, 20 enzymes are referred to a carbohydrate metabolism pathway, including aldehyde dehydrogenase, phosphoglycerate kinase, enolase, acetyl-CoA acetyltransferase (ACAT), hydroxymethylglutaryl-CoA synthase (HMGS), fructokinase, phospholipase C3, glucosidase and chitinase. Twelve other proteins, including ACAT, HMGS, REF, glutamine synthetase, adenosylhomocysteinase, S-adenosylmethionine synthase, cysteine synthase, malate dehydrogenase, aldehyde dehydrogenase and proline iminopeptidase isoform X1, are involved in amino acid metabolism. Another 12 proteins are annotated to be implicated in an energy metabolism pathway. Moreover, nine members related to folding, sorting and degradation function processes: they are heat shock cognate protein (HSP80, spot 108, only one), enolase members (spots 25 and 51, two) and proteasome subunits or their homologs (six). Additionally, five proteins, named V-type proton ATPase subunit B2 isoform X1 (spot 18), V-type proton ATPase catalytic subunit A (spot 132), tubulin alpha-3 (spots 37, 64 and 137), superoxide dismutase (spot 131) and an early-responsive to dehydration protein (spot 30), were annotated to be involved in the cellular processes, which are important members in transport and catabolism pathways. GO annotation results (biological process term) demonstrated that most proteins are involved in the single-organism process (32 proteins), cellular process (31 proteins), response to stimulus (30 proteins), developmental process (21 proteins), multicellular organismal process (19 proteins), cellular component organization or biogenesis (15 proteins) and metabolic process (Table S1).

### 2.3. Determinnation of Isoforms for the Glycosylated Proteins in 2-DE Gels

It is noteworthy that many spots in the 2-DE gels were identified as the product from the same gene, and these glycoproteins are termed as different protein isoforms or protein species for 2-DE gel-based proteomics. In this study, we found that 23 proteins were identified from 65 protein spots, and at least two isoforms were observed for these proteins (Table S1). Among them, REF contains 12 isoforms and these isoforms are the products from two REF genes. Seven spots (spots 14, 55, 83, 91, 125, 128 and 138) were identified as pro-hevein, suggesting that at least seven proteins are found to be the different isoforms of hevein in 2-DE gels. Four spots were identified as two kinds of SRPP, and three spots (spots 101, 111 and 116) were HMGS isoforms.

**Figure 3:**
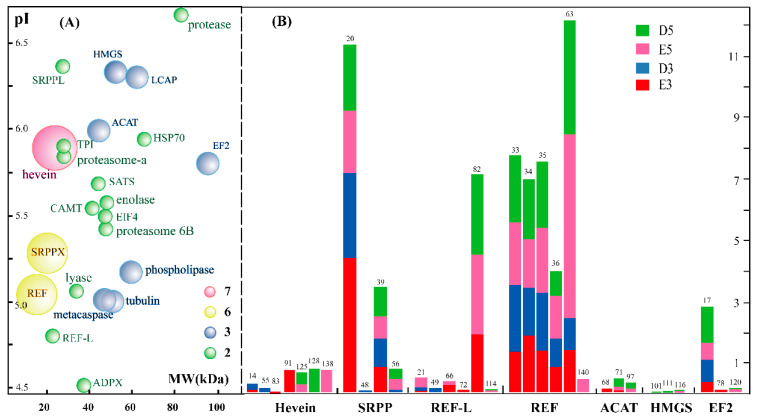
Bubble diagram of all the protein isoforms and comparison of the changed abundance of 2-DE gels upon ethylene application. The glycosylated proteins with at least two isoforms are highlighted as 23 bubbles from 65 protein spots. The bubbles with different sizes and colors stand for different number of protein isoforms identified from different spots (**A**). The accumulation patterns of 7 glycoprotein proteins containing 32 isoforms in the 2DE gels for total rubber latex from the rubber tree treated with water and ethylene for three (D3, E3) and five days (D5, E5) are provided (**B**). The number in the top of the histogram stands for the protein spot number in the 2-DE gels.

Bubble distribution diagram analysis showed that most of these protein isoforms have a pH value varying from 5.0 to 6.0, and their molecular weight ranges from 20 to 60 kDa (Figure 3A). A total of 65 protein spots were identified as protein isoforms, and these isoforms were classified into 23 bubbles, suggesting that these isoforms were the products of 23 genes in the rubber tree genome. Among these bubbles, each of the 13 green bubbles contains two isoforms: they were identified as CAMT, EIF4 (eukaryotic initiation factor 4A-14), SRPP, TPI (triosephosphate isomerase), enolase 1, adenosylmethionine synthase, protease, proteasome, etc. The seven blue bubbles, each of which contains three protein isoforms, in the diagram were known as ACAT (acetyl-CoA acetyltransferase), EF2, HMGS, LCAP (leucine aminopeptidase-1), non-specific phospholipase C3, metacaspase-4 and tubulin alpha-3 chain. Two kinds of REF family members were exhibited as the yellow bubbles, with six isoforms for each. The largest purple bubble is pro-hevein, identified from seven different protein spots in the 2-DE gels. We also noticed that these protein isoforms had different abundance values (Vol%) in the same gel and presented disparate changing patterns after treating with water and ethylene for different days (Figure 3B).

### 2.4. Characterization and Identification of Differentially Accumulated Glycoproteins in the Rubber Latex upon Ethylene Stimulation

**Figure 4:**
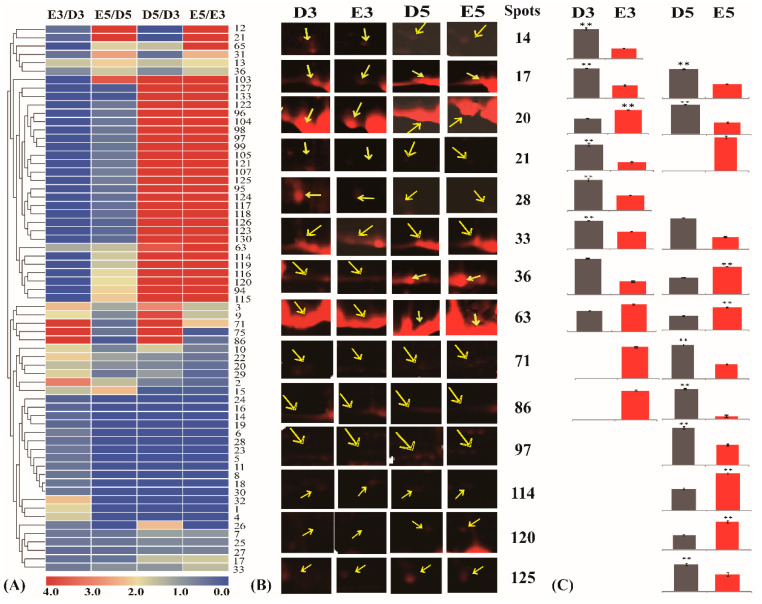
Changing patterns of differentially accumulated glycoproteins (DAGPs) after application of ethylene. The 65 glycosylated proteins with at least 1.5-fold changes were exposed to hierarchical clustering (**A**) to show their changed accumulation patterns after being treated with ethylene or water for three (E3/D3) or five (E5/D5) days. The changed ratios of these proteins after treating with water or ethylene for different times (D5/D3; E5/E3) are also presented. The up- or down-regulated proteins are indicated in red or green, respectively. The intensity of the colors increased with the increased accumulation, as shown in the bar at the bottom. The spot numbers are indicated in the heat plot, which includes five based areas from the top to bottom. The accumulation profiles (**B**) of 14 typical DAGPs in the 2-DE gels and their relatively changed ratios (**C**) are highlighted. The exact position of each protein spot was correspondingly presented in the four 2-DE gel areas.

Based on the abundance of each protein spot in the 2-DE gels, the changed patterns of the identified 144 protein spots were determined, and the abundance of the 65 protein spots presented more than 1.5-fold changes after supplying with exogenous ethylene for three (E3) and/or five (E5) days (Table S2). The ratios of different treatments demonstrated that half of the differentially accumulated glycoproteins (DAGPs) (33 proteins) were sharply increased with the time elongation of the ethylene (E5/E3) or water (D5/D3) treatment. In this study, our interest is focused on the identification of the ethylene-responsive glycoproteins (ERGPs). After three days of ethylene treatment, the abundance of 12 proteins was increased, while 21 proteins decreased. When elongating the treatment time, more ERGPs were identified, including 14 up-regulated and 27 down-regulated glycoproteins (Figure 4A).

Among these ERGPs, adenosylhomocysteinase (spot 2), universal stress protein A-like protein (spot 13) and eukaryotic initiation factor 4A-14 (spot 15) were up-regulated at both two time points. Meanwhile, four down-regulated proteins were detected with ethylene stimulation: they are S-adenosylmethionine synthase 1 (spot 7), elongation factor 2 (spot 17), enolase 2 (spot 25) and PITH domain-containing protein (spot 27). Besides, nucleoredoxin 1 (spot 29) was found to be up-regulated at E3, but down-regulated at E5, while another protein (spot 31) presented an opposite trend. Furthermore, eight proteins, including three REFs and one SRPP, were detected at only one time point. We also noticed that a large portion of identified numbers (48) proteins could only be detected in the gel at three or five days, and all of them were either an ethylene-induced protein spot (IPS) or a disappeared protein spot (DPS) (Table S2). We also selected 14 typical DAGPs and highlighted their detail positions in the 2-DE gels (Figure 4B), displaying their relatively changed ratios (Figure 4C) after different treatments. All these results indicated that different protein spots presented different changed patterns in the four treatments, and many protein isoforms were induced or disappeared with the time elongation of the ethylene treatment.

### 2.5. Functinal Analysis of Differential Glycoproteins in the Rubber Latex upon Ethylene Stimulation

**Figure 5:**
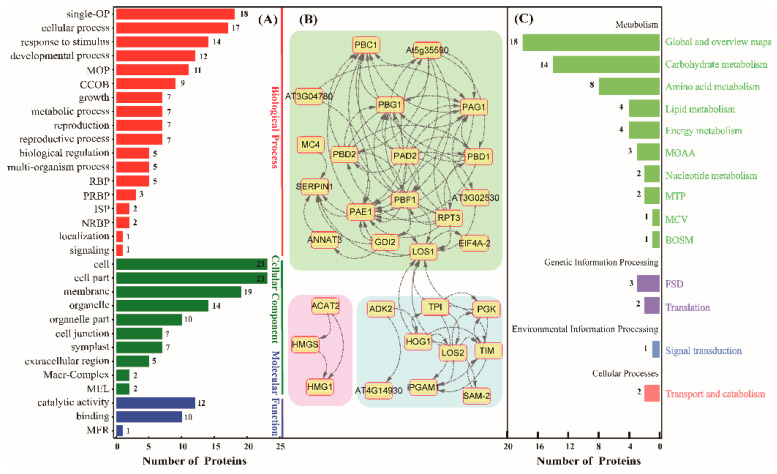
Functional analysis of DAGPs upon ethylene stimulation. The identified 65 DAGPs were exposed to Gene Ontology (GO) (**A**), protein–protein interaction (**B**) and The Kyoto Encyclopedia of Genes and Genomes (KEGG) (**C**) analyses to determine their biological pathways in rubber latex. The abbreviations for these pathways and their detail information are provided in the end section and Table S2.

In order to determine related functions of these DAGPs, we used the AgBase software to perform gene ontology (GO) analysis. These proteins were divided into three GO categories based on biological process, cellular component and molecular function (Figure 5A). These GO categories contain 31 subgroups. Among them, 18 subgroups were included in the biological process, among which 18 proteins are annotated to be involved in the single-organism process (single-OP), followed by cellular process (17 proteins) and response to stimulus with 14 proteins. Furthermore, proteins involved in the developmental process (12 proteins), multicellular organismal process (11 proteins) and cellular component organization or biogenesis (9 proteins) were determined. For cellular component terms, 23 proteins were annotated to be related to the cell part components, which is consistent with the fact that rubber latex is a kind of specific cytoplasm of laticifers. Another 19 proteins were annotated to be the component of the plasma membrane, followed by 14 proteins referring to the organelle. In the molecular function category, 12 proteins showed catalytic activity and 10 proteins possessed strong binding ability (Table S2).

Before submitting the identified 65 glycosylated proteins to the STRING database to obtain their protein–protein interaction networks, the rubber tree-related protein amino acid sequences were used as queries to obtain their orthologous protein sequences of the *Arabidopsis thaliana* protein sequence, with a high confidence value (0.7) in this study. Excluding the disconnected nodes of the network, 30 proteins showed strong interaction relationships with other proteins. According to the associated degrees between different proteins, the network of the 30 proteins was divided into three sub-clusters (Figure 5B). Cluster 1 is the largest interconnection network containing 18 proteins, including LOS1, PAD2, PAG1, PBD2, PBF1 and PBG1, among others. Among them, LOS1, as a ribosomal protein S5/elongation factor G which catalyzes the GTP-dependent ribosomal translocation step during translation elongation, was simultaneously identified from spots 17 and 120 (Table S2). PBF1 was identified as a proteasome subunit from spot 19, and it is a member of the N-terminal nucleophile amino hydrolase superfamily. Cluster 2 includes nine proteins. Among them, LOS2, HOG1 and TIM are the three central nodes. LOS2, identified as the enolase 2 in the rubber latex, is a multifunctional enzyme that acts as a metabolism-related enolase and positively regulates the transcription of cold-responsive genes. HOG1 is known as an adenosylhomocysteinase (spot 2), and it was up-regulated after ethylene treatment at both of the time points in the rubber latex. HOG1 is a competitive inhibitor of S- adenosyl-L-methionine-dependent methyl transferase, which may play a key role in controlling the methylation process by regulating the intracellular concentration of adenosylhomocysteine. Besides, TIM is a chloroplastic triosephosphate isomerase, and it presents a narrower expression range limited to roots. Cluster 3 contains three unique proteins: they are ACAT2, HMGS and HMG1. They are three important members involved in the melavonate (MVA) pathway of NR synthesis. Among them, HMGS condenses acetyl-CoA with acetoacetyl-CoA to form HMG-CoA, which is the substrate of HMG-CoA reductase. HMG1 encodes a 3-hydroxy-3-methylglutaryl coenzyme A reductase, which is involved in melavonate biosynthesis and performs the first committed step in isoprenoid biosynthesis (Table S2).

Furthermore, to understand the high-level functions and utilities of the biological system from molecular-level information, the 65 DAGPs were annotated using the online software KOBAS and Blast KOALA (https://www.kegg.jp/blastkoala), and these proteins were found to be involved in 10 main metabolism pathways (Figure 5C). Among them, 18 proteins are involved in global and overview maps, and 14 proteins are known as the main members of the carbohydrate metabolic process. There are also many members involved in amino acid metabolism (eight proteins), lipid metabolism (four proteins) and energy metabolism (four proteins). At the same time, several proteins were observed to take part in genetic information processing, environmental information processing and transport and catabolism (Table S2).

### 2.6. Functional Verification of the HbHMGS2 Gene

**Figure 6:**
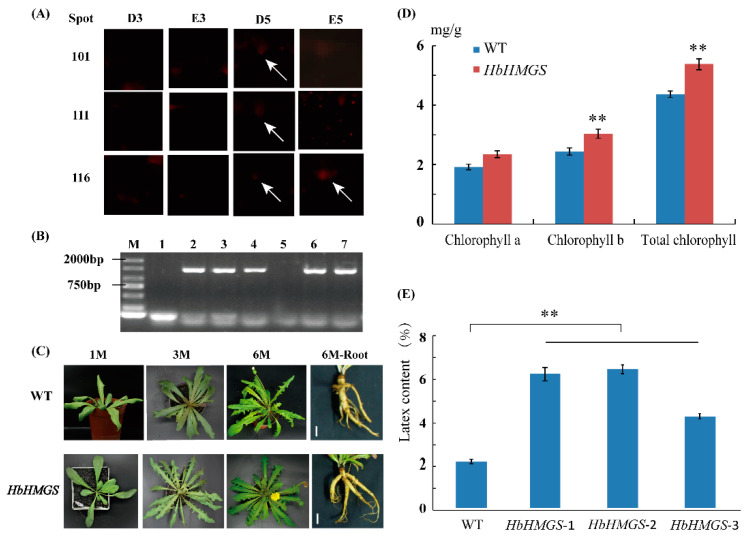
Functional analysis of *HbHMGS2* in the rubber grass *Taraxacum Kok-saghyz*. The protein spots identified as HMGS2 in the 2-DE gels are highlighted, the white arrows indicate the detail location of the corresponding spots that identified as HMGS2 (**A**). The target gene in different transgenic rubber grass lines was determined (**B**) and phenotypes of the transgenic and wild type (WT) plants grown for 1 (1M), 3 (3M) and 6 (6M) months in a greenhouse are presented (**C**). The photosynthetic pigment content in the 6M-old leaves of the transgenic rubber grass and WT plants was determined, and the results proved that the over-expressing of *HbHMGS* can significantly improve the chlorophyll content in the leaves of rubber grass (**D**). The natural rubber content in the 6M roots of wild type and transgenic plants was determined from three independent lines (**E**). Double asterisks indicate the significant difference among different samples.

Based on the preliminary mass spectrometry identification results and functional analysis, we noticed that HMGS2 (ref|XP_021666594.1) in the MVA pathway in the NRB pathway is significantly glycosylated, and the changed patterns in the 2-DE gels after ethylene and water treatments for three days (E3, D3) and five days (E5, D5) were highlighted (Figure 2 and Figure 6A). Statistical results showed that HMGS2 could hardly be detected in the 2-DE gels of the rubber latex treated for three days, however, three protein spots (spots 101, 111 and 116) had been identified as three HMGS protein species in the 2-DE gels of the latex samples for five days (D5 and E5). It is noteworthy that only spot 116 was sharply increased in the rubber latex by ethylene treatment for five days (E5), and the other two spots could only be observed in the D5 latex (Table S1). Our results demonstrated that the abundance for spot 116 was increased 1.9-fold after ethylene treatment for five days (E5) in the 2-DE gel (Table S2). These results indicated that some protein species of HMGS might be the important players for ethylene stimulation of NR production. Therefore, in order to determine the detail functions of HMGS2, we obtained its gene accession number in the rubber tree genome (ref|XP_021666594.1) and over-expressed the gene *HbHMGS2* in a model rubber-producing grass *Taraxacum Kok-saghyz* (TKS) (Figure 6B), and compared their phenotypes in the transgenic TKS plants growing for one (1M), three (3M) and six (6M) months. The results demonstrated that these transgenic grasses and the wild type plants are similar to each other (Figure 6C). However, the main root in the transgenic grasses is bigger than the wild type, and photosynthetic pigment contents, including chlorophyll b and total chlorophyll, are significantly increased over that in the wild type plants (Figure 6D). Furthermore, the NR content in the 6M roots of transgenic plants and wild type was measured. The results demonstrated that the NR content was sharply enhanced and reached about a 3-fold increase in the transgenic TKS roots over the wild type (Figure 6E).

**Figure 7:**
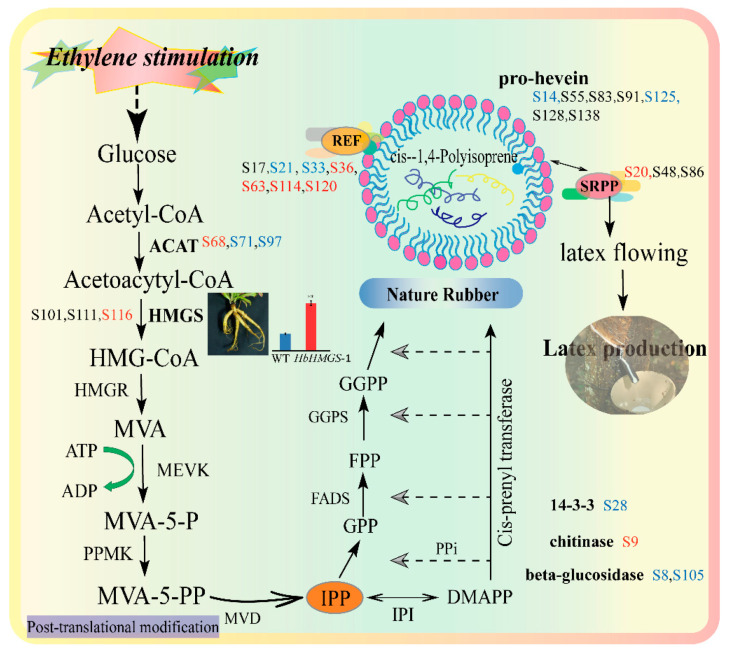
Schematic diagram of the melavonate (MVA) pathway regulated by natural rubber biosynthesis. The identified eight glycosylation proteins in the natural rubber (NR) biosynthesis pathway are highlighted. Among them, seven proteins were identified from different spots (S-) in the 2-DE gels for rubber latex collected from the water- and ethylene-treated rubber trees on the third and fifth day. The red number refers to the up-regulated protein spots, and the blue number stands for the down-regulated protein spots after ethylene stimulation (Table S2).

Based on the analysis of the above proteomics data, a schematic diagram of the rubber latex glycosylation proteins involved in NRB was put forward (Figure 7). In the positively identified 98 unique glycosylated proteins, eight kinds of proteins were found to be related to NRB and/or NR metabolism. They are ACAT (spots 68, 71 and 97), HMGS, REF, SRPP, 14-3-3 protein, pro-hevein, chitinase and beta-glucosidase (Table S1). Among them, ACAT, HMGS, REF and SRPP are four important components in the MVA pathway for NR biosynthesis (Figure 7).

It is noteworthy that ACAT was identified from three independent spots named S68, S71 and S97, and only spot 68 was induced by ethylene in D3 latex, while the other two spots showed a decreased abundance after ethylene stimulation. Several isoforms of REF and SRPP had been determined as glycosylated proteins. Seven protein species from different spots (S17, S21, S33, S36, S63, S114 and S120) were identified as REF. Among them, four REF species (S36, S63, S114 and S120) were increased, but two members (S21 and S33) were decreased after ethylene stimulation for three and/or five days. For SRPP, only one spot (S20) was sharply induced by ethylene for three and five days, the other two spots (S48 and S86) changed not significantly upon ethylene application. Except for chitinase (spot S9), the other three kinds of proteins (including 14-3-3 protein, pro-hevein and beta-glucosidase), which are known to take part in the regulation of NR production, showed a decreased accumulation pattern upon ethylene stimulation (Figure 7). These results indicated that glycosylated proteins might have an important influence on the regulation of NR biosynthesis.

## 3. Discussion

### 3.1. The First Visual Profiling of Glycoproteomics Based on 2-DE Gel Determined Lots of Proteins Involved in Carbohydrate Metabolism in Rubber Latex

Ethylene has been widely applied in agriculture and horticulture as a plant hormone. Ethylene stimulation is considered of great importance for rubber exploitation systems since its effects are favorable to the maintenance of high production levels, and this technology was tested as early as the 1970s, and now it has been a widely used technology in NR production [33]. Surprisingly, the expression levels of several known genes related to rubber biosynthesis did not significantly increase after ethylene stimulation [7], such as farnesyl diphosphate synthase (FADS) [34], cis-isoprene transferase (CPT) [35] and hydroxymethylglutaryl coenzyme A reductase (HMGR) [36]. These results proved that many NRB-related genes may not be induced or even be inhibited by exogenous ethylene stimulation. Therefore, several researchers suggested that ethylene has little direct effect on NRB and the increased latex yield produced by ethylene stimulation might be attributed to the prolongation of latex flow [4,37]. However, at the protein level, some new evidence supports the accelerative effect of ethylene stimulation on NR production [38,39]. Our recently published proteomics results revealed that a large amount of ethylene-responsive latex proteins (ERLPs) are determined in total rubber latex [7] and small rubber particles [8], and many isoforms of NRB-related proteins are sharply induced upon ethylene stimulation. These results indicated that the regulation of rubber latex production by ethylene stimulation might occur not solely at the gene level but also at the protein level, and post-translational modifications (PTMs) of proteins might play crucial roles in controlling the final function of enzymes involved in rubber biosynthesis [7,8]. Among these PTMs, phosphorylation of REF and SRPP isoforms might be crucial for NRB, and small rubber particles may act as a complex natural rubber biosynthetic machine in rubber latex [8].

Protein glycosylation, which is similar to protein phosphorylation [7], is also one important PTM in plants. Glycosylated proteins have been reported to be involved in regulating plant growth and development at the cell level. Arabinogalactan proteins, which are widespread in the plant kingdom, are a class of hydroxyproline-rich glycoproteins that can act on different development aspects of plants, including cell division and expansion, leaf development and reproduction [40]. Hundreds of glycosylated proteins have been identified from the mature stems trapped on Concanavalin A [41] and etiolated hypocotyls [42] in model plant *Arabidopsis thaliana* by 2-DE gel-based glycoproteomics methods, and respectively identified 102 and 126 glycosylated proteins. Among them, many glycosylated proteins are localized in the plant cell wall [41,42]. Meanwhile, cell wall glycoproteomics has been performed to explain how the cell wall peroxidases in response to the changed external environment conditions in the ascorbate-deficient mutant *A. thaliana*, and reveals that the cell wall glycoprotein group is affected by the lack of ascorbic acid, which finally affects the expression of cell wall proteins involved in the pathogen response [43].

It is known that rubber latex contains many macromolecular substances, such as proteins, sugars and alkaloids, and the NRB is a complex process [7,44]. Many proteins involved in the NRB process have been identified by proteomics-based technologies from different latex components [2,7,8,10,11,12,13,15,16,45]. These proteomics results revealed that rubber latex has a high protein content, and different kinds of PTMs have been determined in rubber latex to efficiently regulate NRB [7,8]. Among them, glycosylation is one of the most important kinds of protein PTMs, which plays crucial roles in plant growth and development processes, as well as NRB and metabolism. Although some pioneering research on latex glycoproteins have done, a systematic study on the latex glycoproteins upon ethylene is still limited. In this study, the first visual profiling of glycoproteomics was performed by a combination of 2-DE and mass spectrometry technologies to identify the glycosylated proteins in rubber latex after ethylene treatment for three and five days. Finally, 144 species were identified as glycosylated proteins from rubber latex and they were encoded by 98 genes in the rubber tree genome. Our proteomics results demonstrated that these glycosylated rubber latex proteins are mainly involved in carbohydrate metabolism, energy metabolism, degradation function and cellular processes, and might be important for rubber metabolism (Table S1).

### 3.2. Glycoproteomics of Rubber Latex Revealed Many Ethylene-Responsive Glycoproteins are Important for Nature Rubber Biosynthesis

Among the identified 98 gene products, 65 protein species were found to be ERGPs. KEGG pathway analysis revealed that 14 of these ERGPs are involved in carbohydrate metabolism: they are lactoylglutathione lyase, phosphoglycerate mutase, phosphoglycerate kinase, enolase, phosphate uridylyltransferase, fructokinase, aldehyde dehydrogenase, phospholipase C3 and glutamine synthetase. Lactoylglutathione lyase, also known as glyoxalase, is an enzyme that catalyzes the isomerization of hemithioacetal adducts. It was identified from spots 98 and 126 (Table S2) of rubber latex in this glycoproteomics study, and this enzyme was also detected from the 2-DE gel of milky sap from *Chelidonium majus* [45] and total latex of the rubber tree [7]. Its expression in rubber latex serum was obviously changed when triggered by ethephon treatment to maintain and regulate the plant redox homeostasis process and defense system in the rubber tree [46].

Phosphoglycerate mutase is known to exert a certain physiological impact in plant cells. It was identified as a metabolic protein from lettuce latex sap [47], rubber latex [13] and the washed solutions from rubber particles [48]. In this proteomics study, we found it was glycosylated (spot 3) in the 2-DE gels (Figure 2) and increased by 2.3-fold after ethylene stimulation for three days (Table S2). Phosphoglycerate kinase, which catalyzes the formation of ATP from ADP and 1,3-diphosphoglycerate for cell metabolism, was determined as an ERGP from spot 12 (Figure 2) in this proteomics study. It was also identified from the latex of *C. majus* in different phases of plant development [45], lettuce latex [47], rubber tree latex [13,49], different washed solutions from rubber particles [8,48] and different rubber latices after ethylene application, in which, phosphoglycerate mutase was determined as a phosphorylation protein [7]. These recently published results indicate that phosphoglycerate mutase and phosphoglycerate kinase are two important components in ethylene stimulation of rubber latex metabolism.

Enolase, typically localized in the cytosol, is widely known as one of the glycolytic enzymes and catalyzes the conversion of 2-phosphoglycerate and phosphoenolpyruvate in glycolysis. This enzyme is ubiquitous in rubber-producing plants and many living organisms [7], and it has also been identified by several proteomics researches from the latex of rubber tree as a new allergen named Hev b 9 [50,51,52], an ethylene-responsive protein [7,15,46], or a high-abundance protein in rubber particles [13,48]. It was identified by mass spectrometry from the latex of *Opium poppy* [49] and the milky sap of *C. majus* [45]. Phosphate uridylyltransferase was determined as an ERGP in rubber latex in this study (Table S2), and it was also identified from the washed solutions of rubber particles [53] and lettuce latex in a proteomics study [47].

Fructokinase is a kinase that catalyzes the transfer of phosphate groups to fructose and can phosphorylate the sugar at the C-1 position, and thus is considered as one of the rate-limiting enzymes of glycolysis [54]. Recent rubber latex proteomics data revealed that fructokinase can be induced by ethylene treatment [55], but in this study, we found this enzyme can be glycosylated and its abundance was decreased after ethylene stimulation for five days (Table S2). Aldehyde dehydrogenase is a kind of oxidizing enzyme that is involved in detoxification of both exogenous and endogenous aldehyde substrates through NAD(P)^+^-dependent oxidation. In a proteomics study, it was identified from *Colletotrichum gloeosporioides*, which is a hemibiotrophic fungi that could cause anthracnose in *Hevea brasiliensis* [56].

Phospholipase C is an enzyme that hydrolyzes plasma membrane phospholipids at the third position of the glycerol backbone. Its gene expression was up-regulated in the latex of a tapping panel dryness [57] and a high-yielding rubber tree [54]. Proteomics analyses revealed that this protein is highly abundant in the washed solutions of rubber particles [48] and was significantly induced in rubber latex after ethylene application [55]. Another enzyme, glutamine synthetase, plays an important role in metabolizing nitrogen by catalyzing the reaction of condensation of glutamate and ammonia to form glutamine, and the glutamine–glutamate synthase cycle might be the major pathway for the amino acid and protein synthesis required for natural latex regeneration [4]. Previous proteomics studies demonstrated that glutamine synthetase is a high-abundance protein in the latex of lettuce [58], *O. poppy* [49], the washed solutions from rubber particles [48] and rubber latex serum [59]. The activity and mRNA level of glutamine synthetase are observed to be increased after ethylene stimulation in latex cells [60]. Comparative proteomics analyses proved that accumulation of this enzyme is significantly up-regulated in rubber latex upon ethylene treatment [7,15]. These published results and our glycoproteomics of rubber latex in this study revealed that ethylene-responsive glycoproteins are important for NR production.

### 3.3. Glycosylation of Different Isforms of NRB-Ralated Proteins Mgiht Play Different Roles and HbHMGS2 Gene Is Crucial for Nature Rubber Production

Several proteomics studies have been conducted and more NRB-related enzymes were identified by mass spectrometry from natural rubber latex and rubber particles. These NRB-related proteins are AACT, HMGS, MEVK, FADS, GGPS, CPT, REF, SRPP, etc., and most of them were identified as membrane-attached proteins from purified rubber particles [7,8,10,11,12,13,15,16]. A pioneering proteomics study of rubber particles identified 186 proteins, including REF, SRPP and cis-prenyl transferase [11]. Comparative proteomics of large and small rubber particles revealed 22 gene products, including SRPP, REF, HMGS, HSP70 and phospholipase D, are differentially accumulated [10]. NRB, beginning with isopentenyl pyrophosphate (IPP) synthesis in the MVA pathway, is a typical isoprenoid metabolic process. In the early steps, acetyl-CoA C-acetyltransferase (ACAT) is important for generating acetoacytyl-CoA, where it catalyzes acetyl-CoA to form acetoacetyl-CoA, which is the first step in the MVA pathway [61,62]. Then, HMGS and HMGR activate the supply of mevalonate substrates [7,62]. In this proteomics study, eight kinds of NRB-related proteins in the MVA pathway were positively identified from rubber latex, and some isoforms of these glycosylation proteins were detected to change significantly after ethylene stimulation for three and/or five days (Table S2). Among them, three ACAT isoforms were detected from spots 68, 71 and 97, respectively (Figure 7). These protein isoforms may generate by alternative splicing and different kinds of post-translational modifications [55]. Three ACAT genes were determined in the rubber tree genome [63]. Our previous proteomics results of total rubber latex revealed that both the gene and protein accumulation of ACAT are depressed upon ethylene stimulation, but one protein isoform is induced [7]. In another proteomics study on small rubber particles, one isoform was identified as ACAT from SRPs, and its abundance was induced upon ethylene treatment [8], which is consistent with our previously published latex proteomics results [7] and the new observation in this study (Figure 7).

During the rubber elongation process, REF and SRPP are two key members [62], which are closely combined with the membrane of rubber particles [43]. SRPP, a rubber biosynthesis-related protein that is expressed mainly in small rubber particles [64], has been reported to play more important roles than REF for NBR [10,65]. Comparative localization results of SRPP and REF reveal that the rubber biosynthesis capability in rubber laticifer is mostly concentrated in small rubber particles by SRPP [61,66]. SRPP can recruit CPT to the endoplasmic reticulum to interact with CTP1-REF bridging protein [67]. Interaction network analysis of rubber particles demonstrates that CTP1, REF and CTP1-REF bridging protein (HRBP) may play crucial roles in NRB [12], and they are associated with the endoplasmic reticulum [67]. In a previously published proteomics study, we have detected 18 REF/SRPP gene family members in the rubber tree, and their genes exhibit distinct expression patterns in different tissues in a single 205-kb genome site [16]. In 2-DE gels, we also have determined 28 protein isoforms from five REF/SRPP members, and multiple protein isoforms have been identified [16]. Our proteomics data revealed that the gene expression and protein accumulation for most of these REF/SRPP members are decreased or not changed after ethylene treatment. However, individual isoform members display different changed patterns, and some family members or protein isoforms are sharply increased by ethylene stimulation in rubber latex [7,16] and small rubber particles [8]. Among them, 14 REF isoforms are significantly changed after ethylene treatments, including 10 sharply induced ones. For SRPP, five protein isoforms are increased upon ethylene stimulation [7]. However, in this glycoproteomics of rubber latex, three glycosylated SRPP isoforms were respectively identified from spots 20, 48 and 86 in the 2-DE gels, and only one member (spot 20) was detected as the induced protein species after ethylene treatment for three days (Figure 7). Compared with SRPP, many more REF isoforms were detected to be the induced glycosylation proteins. Among the identified seven REF isoforms, four members (spots 36, 63, 114 and 120) were determined as ethylene-induced proteins (Table S2). These results demonstrated more REF isoforms have been glycosylated in rubber latex and glycosylation modification of REF might play more important roles than SRPP after ethylene stimulation for different times.

Beta-glucosidase is a key enzyme component present in the cellulase enzyme complex and completes the final step during cellulose hydrolysis, which is essential for complete hydrolysis of cellulose into glucose. This reaction is always under control as it gets inhibited by its product glucose, thus is a major bottleneck in the efficient biomass conversion by cellulase. Proteomics analysis of the vegetative vacuole from *A. thaliana* results in the identification of three identified beta-glucosidases containing endoplasmic reticulum retention signals at their respective C termini, which is responsible for the vacuolar localization of target proteins under certain conditions [68]. In this proteomics study, we noticed two isoforms of this enzyme were also detected as glycosylated proteins, and their accumulation was significantly reduced after ethylene application (Figure 7). It is known as a major protein from proteomics analyses of the latex from *H. brasiliensis* [13,48], *C. majus* [45] and *O. poppy* [49], and it is an effect factor on the inhibition of lectin-like protein-induced rubber particle aggregation [69].

Chitinase and hevein, as well as glucosidase, are key activators of lutoid-mediated rubber particle aggregation and sequential latex coagulation in rubber latex [2,65]. Hevein, or its precursor pro-hevein, is highly abundant in lutoids protein [70]. Both hevein and pro-hevein showed a strong chitin binding ability [65]. Immuno-blotting analysis of hevein revealed both mature hevein and a 20-kDa protein recognized by an N domain-specific antibody [70]. Our comparative proteomics of primary and secondary lutoids has revealed that the decreased accumulation of glucosidase and hevein in ethylene-treated latex may inhibit rubber particle aggregation, thus maintaining the latex flow and resulting in an enhanced rubber latex yield [2]. In this proteomics study, we further proved that several isoforms of pro-hevein and beta-glucosidase can be glycosylated and much of their abundance is sharply decreased after ethylene application (Table S2), thus helping to inhibit rubber particle aggregation in the rubber latex production process.

In an early step of the MVA pathway, HMGS activates the supply of mevalonate substrates and demonstrates a positive response to IPP substrate. Therefore, it is considered as a key enzyme for NRB in the rubber tree [7,39,62]. In the recently published rubber tree genome, two gene family members, named *HMGS1* and *HMGS2,* are characterized [63]. It was reported that HMGS gene expression and enzyme activity are significantly enhanced upon the addition of ethylene [39], and the *HbHMGS1* promoter is found to play important roles in regulating ethylene-mediated gene expression [71]. Over-expression of *BjHMGS1* can up-regulate the genes in sterol biosynthesis and enhance the total sterol production and stress tolerance ability in Arabidopsis [72]. At the enzyme level, the rubber biosynthetic pathway is coordinately regulated by both the activities of HbHMGS and HbHMGR [71], and the isoenzyme derived from the HbHMGS1 transcript is likely involved in the biosynthesis of rubber in laticiferous cells [39]. Therefore, HMGS may play a significant role in controlling isoprenoid and rubber biosynthesis in the rubber tree.

However, our recently published proteomics data showed a different changed pattern in that the gene expression and protein accumulation levels of HMGS are down-regulated or not significantly improved upon treatment with exogenous ethylene [7]. Proteomics analysis of rubber particles revealed that HMGS is expressed more predominantly in small rubber particles than large rubber particles [10], which is consistent with the fact that small rubber particles have higher activity of rubber biosynthesis than the large ones. In this glycoproteomics study, three protein isoforms (spots 101, 111 and 116) of HMGS2 (ref|XP_021666594.1) were identified from the 2-DE gels, but only one member (spot 116) was significantly increased after ethylene treatment for five days (Table S2). Our transgenic results proved that the natural rubber content in the mature roots of the over-expressed *HMGS2* gene rubber-producing grass TKS is sharply improved (Figure 6), and these new data showed that the gene *HbHMGS2* is really a key member for NBR in both the *Hevea* rubber tree and rubber grass TKS. The combination of the recently published results with our new proteomics data demonstrated that glycosylation modification of some protein isoforms is important for NRB and HMGS2 might positively correlate with the natural rubber product in the rubber tree and rubber grass.

In conclusion, in this study, comparative proteomics of rubber latex treated with ethylene for three and five days was performed, and finally resulted in the identification of 65 differentially accumulated proteins from the 144 glycosylated proteins. Both GO functional annotation and KEGG pathway analysis results demonstrated that these DAGPs are mainly involved in the functions and pathways related to the biosynthesis of rubber latex, including cell parts, and membrane components. Our results also indicated that glycosylation of HMGS2 protein might play important roles in the biosynthesis of rubber latex. The gene function verification results showed that the latex content of *HbHMGS2* transgenic plants was significantly higher than that of wild type plants, indicating that the *HbHMGS2* gene is a key member in the regulation of the ethylene-stimulated NBR by post-translational glycosylation of some HMGS2 isoforms. This glycoproteomics study of rubber latex may give some new insights on the regulation mechanism of rubber latex biosynthesis under ethylene stimulation and provide more theoretical support for the further usage of ethylene as a stimulant in natural rubber production.

## 4. Materials and Methods

### 4.1. Plant Material and Ethylene Treatments

Total latex protein samples were obtained from 90 newly tapped mature rubber plants (8-year-old *H. Brasiliensis* Mull. Arg., clone RY 7-33-97) which were grown at an experimental farm of the Chinese Academy of Tropical Agricultural Sciences in Danzhou City, Hainan Province, China. The rubber trees never treated with ethylene were selected and randomly divided into four groups. The tree cuts were respectively treated with ethephon (3%, *v*/*v*) and double-distilled water (ddH_2_O, the control) as described [7], and the latex samples were respectively collected from each rubber tree at two time points: three days after treatment (D3, E3) and the fifth (D5, E5) day after ethylene or ddH_2_O treatments. For each treatment, the mixed latex samples were obtained from the corresponding five rubber trees. The latex droplets were collected in an iced glass beaker, then immediately frozen in liquid nitrogen and stored for further use at −80 °C.

### 4.2. Protein Extraction and Electrophoresis

The extraction of the total latex protein for the rubber was performed using the BPP method as described previously [7,8]. Then, the protein concentration of the total latex was determined following the Bradford method by a spectrophotometer (Shimadzu UV-160, Kyoto, Japan) and bovine serum albumin (BSA) was used as a standard. An amount of 20 μL of protein lysate containing 30 μg of total protein for each sample was loaded with 10 μL of 3 × loading buffer for the one-dimensional discontinuous SDS-PAGE electrophoresis, and the electrophoresis conditions were set as: 16 °C, 6 W, for 60 min, and then 16 °C, 8 W, for 4 h. The Image Scanner was used for scanning after Coomassie blue staining, then the image was analyzed with the Image Master 2D Platinum software (GE Healthcare, Uppsala, Sweden).

In the two-dimensional electrophoresis system, 455 μL sample containing about 1000 μg protein was loaded. The first isoelectric focusing was performed using an IEF-100 focusing instrument from Hoefer, a linear IPG gel strip (pH 4–7, 24 cm in length). Then, the gel was equilibrated with an equilibration solution containing 1% DTT (50 mmol/L Tris-HCl, PH 8.8, 6 mol/L urea, 30% glycerol, 2% SDS, 0.02% bromophenol blue) and 4% iodoacetamide liquid for 15 min. Following this, the remaining equilibration buffer was washed with ddH_2_O, and second-direction vertical plate gel electrophoresis was performed in an Etan Daltsix electrophoresis apparatus (GE Healthcare), with the program set to: 1.5 W/gel, 1 h; 8 W/gel, for 6 h, and three biological replicates were conducted for each sample. Finally, the gels were stained with Coomassie blue and the results were observed as mentioned above.

### 4.3. Glycoproteomics Analysis

Glycosylated proteome staining was carried out with Pro-Q^®^ Glycoprotein Blot Stain Kit (Molecular Probes, Eugene, WI, USA). Firstly, 500 mL stationary liquid (50% methanol, 5% acetic acid) was used to fix the gel with soft shaking at room temperature overnight; then, 500 mL solution containing 3% glacial acetic acid was used as the rinsing solution, and was gently shaken for 20 min twice. Then, we gently shook 500 mL oxidizing solution (periodic acid in 3% acetic acid) to immerse and oxidize for 1 h, followed by rinsing with 500 mL rinsing solution for 15 min, repeated three times. After staining with 250 mL of Pro-Q Emerald 300 for 2.5 h and 15 min of rinse (repeated once for gel imaging), the result could be observed with a 300 nm UV transmission.

### 4.4. Protein Identification via Mass Spectrometry

After the target protein spots were cut out, the in-gel digestion was performed as described [8]. After enzymatic digestion, these peptides were collected and identified by the AB 5800 MALDI-TOF/TOF mass spectrometry (MS) instrument (AB SCIEX, Foster City, CA, USA). The ProteinPilot Software (Version 4.5) and a Mascot Algorithm (version 2.3) were used to search against the *Hevea* genome scaffolds (BioProject ID: PRJNA80191, www.ncbi.nlm.nih.gov/nuccore/448814761) and the draft genome (GenBank: AJJZ01000000) with 46,718 sequences and 17,435,757 residues [63]. The parameters were set as: precursor tolerance 300 ppm, 0.3 Da tolerance as MS/MS fragment, trypsin as the enzyme, carbamidomethylation as the fixed modification (C) and oxidation (M) as the variable modification. Detailed information on all identified protein spot mass searches can be found in Supplemental Figure S1 and Table S1.

### 4.5. GO and KEGG Annotations for the Identified Glycosylated Proteins

In order to clarify the specific functions of these identified glycosylated proteins, we performed Gene Ontology (GO) function and Kyoto Encyclopedia of Genes and Genomes (KEGG) pathway annotation for the identified glycosylated proteins. The related protein sequences were submitted to AgBase (Version 2.0) (https://agbase.arizona.edu) for the GO function annotation, and the annotated results were visualized by Omicshare analysis (https://www.omicshare.com). Meanwhile, the KEGG pathway was annotated with the KOBAS 3.0 database (http://kobas.cbi.pku.edu.cn/kobas3). The detailed information is shown in Supplementary Table S2.

### 4.6. Protein-Protein Interaction (PPI) Analysis

In order to further identify the correlation between these proteins, a protein–protein interaction analysis was performed with the web tool STRING 11.0 (http://stringdb.org). Before submitting the data to the STRING database, the amino acid sequences of the identified proteins were aligned with the *Arabidopsis thaliana* database to obtain the homologous protein sequences. Finally, the PPI network was visualized by the Cytoscape 3.7.2 software. All the details can be found in Supplementary Table S2.

### 4.7. Phenotypic Analysis of the Transgenic Plants and Determination of Photosynthetic Pigment and Latex Content

In this study, the rubber grass *Taraxacum Kok-saghyz* (TKS) was selected as the model plant as a transgenic receptor material for further function analysis of the target protein HMGS as described [73]. The phenotypes of the wild type rubber grass and the *HbHMGS* over-expressed transgenic plants at the age of 1 month, 3 months and 6 months were compared. The contents of photosynthetic pigment and latex content were measured when the plant was 6 months old. The photosynthetic pigment content was measured with the method of 90% acetone extraction as described [74], and then infrared spectroscopy was used to measure the content of the natural rubber in the 6M roots of the rubber grasses as described [75].

### 4.8. Statistical Analysis

The data of the protein expression levels and the fold changes in the 2-DE gels are the average of three biological replicates. Student’s *t*-test and one-way analysis of variance (ANOVA) followed by Tukey’s multiple comparison test (*p* < 0.05) were performed using SPSS18.0. The mean differences were significant by *t*-test at *p < 0.05*, whilst the least significant difference at the 5% level was considered statistically significant among different treatments. The following asterisks indicate the results of significance testing: * *p* < 0.05 and ** *p* < 0.01. Different colors in the graphs indicate differences. Data represent mean values and error bars are means ± standard deviation (SD).

## Supplementary Materials

The following are available online at https://www.mdpi.com/1422-0067/21/15/5282/s1. Figure S1: Detail information for MS identification of proteins in the 2-DE gel of the glycosylation spots; Table S1: All proteins identified from 2-DE gels and the potential functions; Table S2: Determination and functional analysis of the DAGPs in rubber latex upon ethylene stimulation.

## Abbreviations

ACAT	acetyl-CoA acetyltransferase
DMAPP	elongation factor 2-like protein
EF2	Three letter acronym
FPP	farnesyl pyrophosphate
FPS	farnesyl pyrophosphate synthase
GGPP	geranylgeranyl pyrophosphate
GPP	geranyl pyrophosphate
GPS	geranyl pyrophosphate synthase
Hevein	pro-hevein
HMG-CoA	hydroxymethylglutaryl-CoA
HMGR	HMG-CoA reductase
HMGS	hydroxymethylglutaryl-CoA synthase
IPP	isopentenyl pyrophosphate
IPI	IPP isomerase
MEVK	mevalonate kinase
MVA	mevalonate acid
MVA-5-P	mevalonate-5-phosphate
MVA-5-PP	mevalonate pyrophosphate
MVD	mevalonate pyrophosphate decarboxylase
PPMK	MVA-5-P kinase
SRPP	small rubber particle protein
REF	rubber elongation factor
REF-L	rubber elongation factor-like protein
14-3-3	14-3-3 protein
