# Supplementary material for: Identification and Characterization of Glycoproteins and Their Responsive Patterns upon Ethylene Stimulation in the Rubber Latex
Source: Int J Mol Sci. 2020 Jul 25;21(15):5282. doi: 10.3390/ijms21155282 (PMC7432319; doi:10.3390/ijms21155282)
Supplement: Supplementary file 1 [file ijms-21-05282-s001.zip › ijms-879232-SI-to proofreading/Figure S1 MS identification of all the glycosylated proteins in 2-DE gel.docx]

**Detail information for MS identification of proteins in the 2-DE gel of the glycosylation spots**

**Spot No.:1**

**Protein name: aldehyde dehydrogenase family 7 member B4-like [Hevea brasiliensis]**

**Peptide sequences:**

R.KEYEFLTEIGLSER.N(108);

K.EYEFLTEIGLSER.N(88);

K.ILPEGIGEVQEIIDMCDFAVGLSR.Q(109);

K.VGLMVQQTVNQR.Y(47);

R.SVLFAAVGTAGQR.C(71);

K.FQTLQEAIEINNSVPQGLSSSIFTR.K(131)

**Accession No.: ref|XP_021680058.1|**

**Mascot score: 557 Sequence coverage %: 16%**

**Calculated Mr: 57876 Calculated pI: 5.65**

**PFF Searched Score:**

**
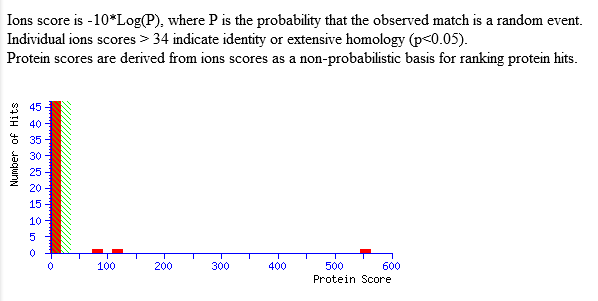
**

**Matched peptide sequences: shown in Bold Red:**

**
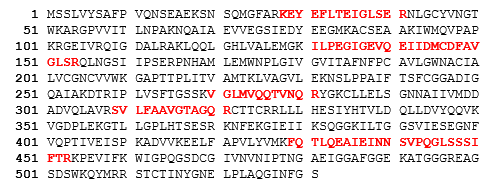
**

**Spot No.:2**

**Protein name: aldehyde dehydrogenase family 7 member B4-like [Hevea brasiliensis]**

**Peptide sequences:** R.LEIELAEVEMPGLMACR.A(114);R.WCSCNIFSTQDHAAAAIAR.D(56);K.GETLQEYWWCTER.A(64);K.TGQLPDPSSTDNAEFQIVLTIIR.D(35);K.SKFDNLYGCR.H(36);R.HSLPDGLMR.A(23);K.DIIMVDHMR.K(30);R.WVFPETNSGIIVLAEGR.L(87);K.EQADYISVPIEGPYKPPHYR.Y(44)

**Accession No.: ref|XP_021689860.1|**

**Mascot score: 496 Sequence coverage %: 28%**

**Calculated Mr: 53864 Calculated pI: 5.77**

**PFF Searched Score:**

**
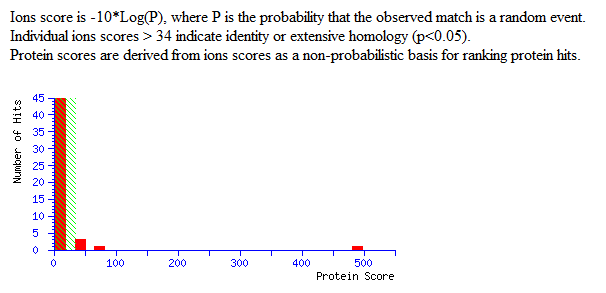
**

**Matched peptide sequences: shown in Bold Red:**

**
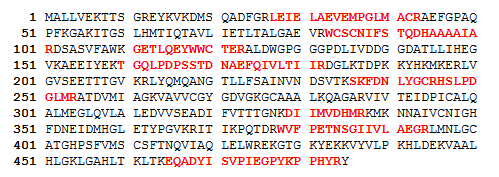
**

**Spot No.:3**

**Protein name: 2,3-bisphosphoglycerate-independent phosphoglycerate mutase-like isoform X1 [Hevea brasiliensis]**

**Peptide sequences:**

R.AHGTAVGLPTEDDMGNSEVGHNALGAGR.I(91);K.YIQESFASGTLHLIGLLSDGGVHSR.L(88);R.GWDAQVLGEAPHK.F(50);K.ANDQYLPPFVIVDENGKPVGPVVDGDAVVTFNFR.A(116);K.ALEYENFDKFER.V(69);K.LPSHYLVSPPEIER.T(110);K.SGNIQILTSHTLQPVPIAIGGPGLAPGVR.F(117)

**Accession No.: ref|XP_021684329.1|**

**Mascot score: 645 Sequence coverage %: 27%**

**Calculated Mr: 61257 Calculated pI: 5.81**

**PFF Searched Score:**

**
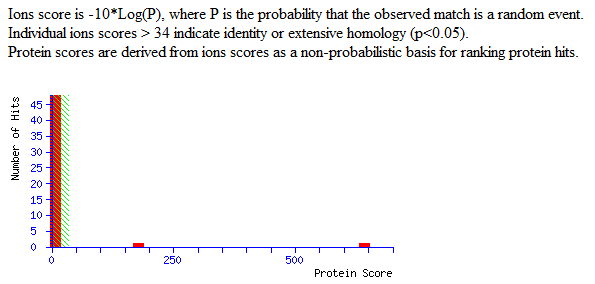
**

**Matched peptide sequences: shown in Bold Red:**

**
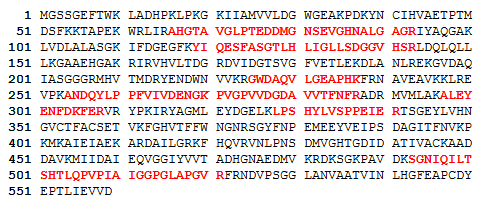
**

**Spot No.:4**

**Protein name: DEAD-box ATP-dependent RNA helicase 38 [Hevea brasiliensis]**

**Peptide sequences:**

K.TTCFVLGMLSR.I(71);K.YTGISSECAVPMDSGNNDR.S(99);R.SRPPIFAQVVIGTPGTIK.R(78);K.DGFQDDSLR.I(68);R.LNAQCQVLLFSATFDETVK.N(57);K.ALVDLGYEVTTIHGALTHEDR.D(106);K.ALVDLGYEVTTIHGALTHEDRDK.I(117);K.EFKDGLTQVLISTDVLAR.G(68);K.DGLTQVLISTDVLAR.G(124);K.YETPSEPHYEVYLHR.I(89);R.KGAVFNFVMTDR.D(76);K.GAVFNFVMTDR.D(51)

**Accession No.: ref|XP_021677409.1|**

**Mascot score: 1010 Sequence coverage %: 28%**

**Calculated Mr:57121 Calculated pI:5.54**

**PFF Searched Score:**

**
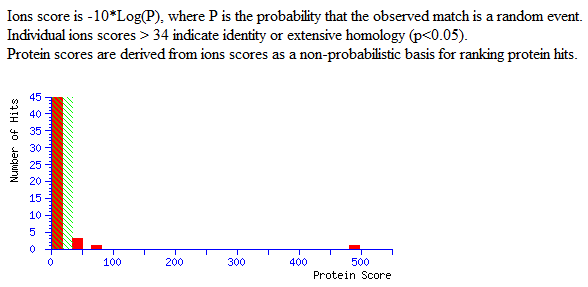
**

**Matched peptide sequences: shown in Bold Red:**

**
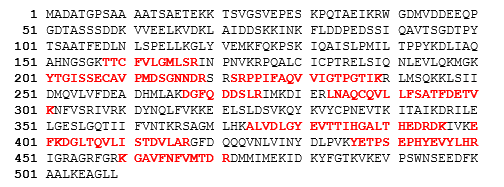
**

**Spot No.:5**

**Protein name: glutamine synthetase cytosolic isozyme 2 isoform X2 [Hevea brasiliensis]**

**Peptide sequences:**

R.GNNILVMCDAYTPAGEPIPTNKR.H(56);K.IFSHPDVVAEEPWYGIEQEYTLLQR.D(118);R.HDGGYEVIKK.A(38);R.HKEHIAAYGEGNER.R(112);K.EHIAAYGEGNER.R(59);R.HETADINTFLWGVANR.G(137)

**Accession No.: ref | XP_021654887.1 |**

**Mascot score: 516 Sequence coverage %: 24%**

**Calculated Mr: 39304 Calculated pI: 6.32**

**PFF Searched Score:**

**
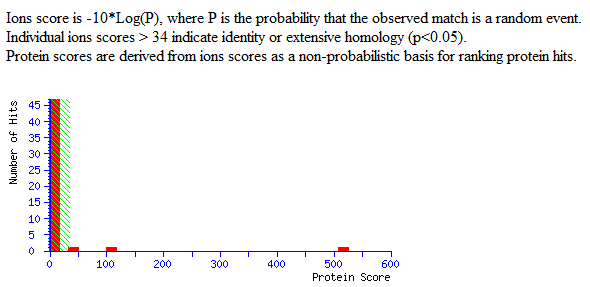
**

**Matched peptide sequences: shown in Bold Red:**

**
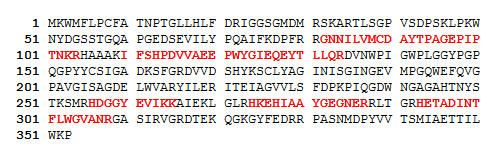
**

**Spot No.: 6**

**Protein name: 14 kDa zinc-binding protein [Hevea brasiliensis]**

**Peptide sequences:**

K.AAALTAVPSDSPTIFDK.I(29);K.EIPCNVVYEDDKVLAFK.D(9);K.DISPQAPTHILIIPK.V(34);K.QEGLEDGFR.I(46);R.IVINDGPNGCQSVYHLHIHLLGGR.Q(25)

**Accession No.: ref | XP_021639926.1 |**

**Mascot score:144 Sequence coverage %: 56%**

**Calculated Mr: 16097 Calculated pI: 6.29**

**PFF Searched Score:**

**
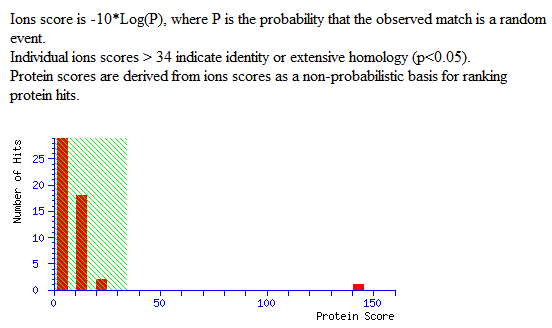
**

**Matched peptide sequences: shown in Bold Red:**

**
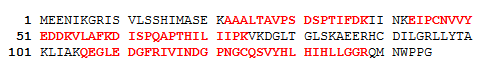
**

**Spot No.: 7**

**Protein name: S-adenosylmethionine synthase 1-like [Hevea brasiliensis]**

**Peptide sequences:**

K.VLVNIEQQSPDIAQGVHGHFTK.R (108)

K.TQVTVEYYNDNGAMVPVR.V(76); R.VHTVLISTQHDETVTNDEIAADLK.E (105); K.TIFHLNPSGR.F(73); R.FVIGGPHGDAGLTGR.K (133); K.TAAYGHFGR.D (73)

**Accession No.: ref|XP_021664714.1|**

**Mascot score: 568 Sequence coverage %: 24%**

**Calculated Mr: 43654 Calculated pI:5.68**

**PFF Searched Score:**


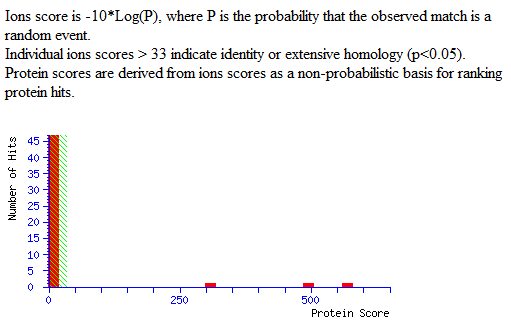


**Matched peptide sequences: shown in Bold Red:**

**
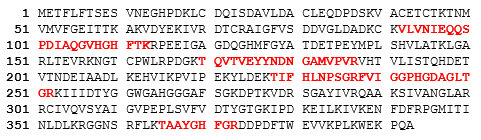
**

**Spot No.:8**

**Protein name: beta-glucosidase 42 isoform X1 [Hevea brasiliensis]**

**Peptide sequences:**

R.SDFPPNFLFGVATSAYQIEGGCR.E(158);

K.YFAIYADTCFASFGDR.V(97);

R.EPFLASHHQILAHATAVSIYR.S(26);

K.FSEEDKELLR.N(67);

R.NSLDFIGLNHYSSR.F(111);

K.WEDGEPIGER.A(80);

R.AASEWLYVCPWGLR.K(88);K.VLNYIVQR.Y(33);

K.SSAYWFMR.F(39)

**Accession No.: ref|XP_021636834.1|**

**Mascot score: 697 Sequence coverage %:25 %**

**Calculated Mr: 56484 Calculated pI: 5.49**

**PFF Searched Score:**

**
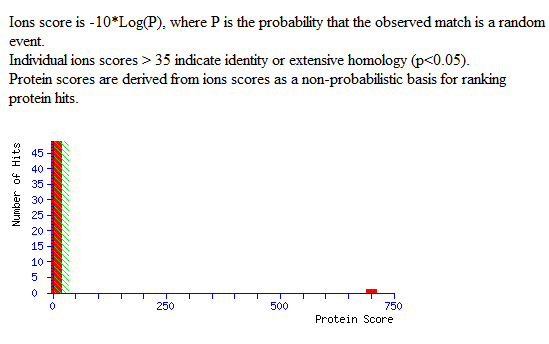
**

**Matched peptide sequences: shown in Bold Red:**

**
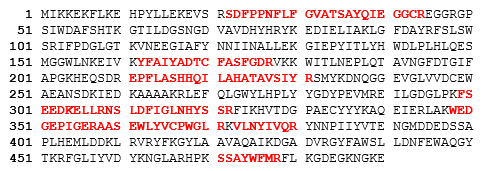
**

**Spot No.:9**

**Protein name: endochitinase-like [Hevea brasiliensis]**

**Peptide sequences:**

K.STFEEFLK.H(13);

R.GPIQLTWNYNYGQCGR.A(58)

**Accession No.: ref|XP_021664500.1|**

**Mascot score: 71 Sequence coverage %:8 %**

**Calculated Mr:33444 Calculated pI:4.9**

**PFF Searched Score:**

**
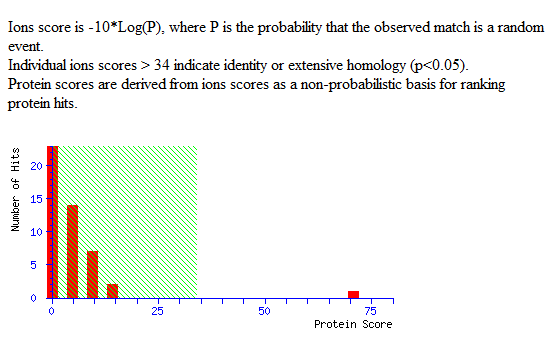
**

**Matched peptide sequences: shown in Bold Red:**

**
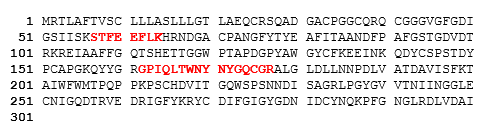
**

**Spot No.:10**

**Protein name: carbonic anhydrase 2-like [Hevea brasiliensis]**

**Peptide sequences:**

K.FLVFACSDSR.V(44);

R.VSPSHVLDFQPGEAFMFR.N(58);

R.NIANLVPAFNQLR.Y(72);

K.EAVNLSLVNIQSYPYVR.A(112)

**Accession No.: ref|XP_021669847.1|**

**Mascot score: 266 Sequence coverage %: 23%**

**Calculated Mr: 28588 Calculated pI: 6.07**

**PFF Searched Score:
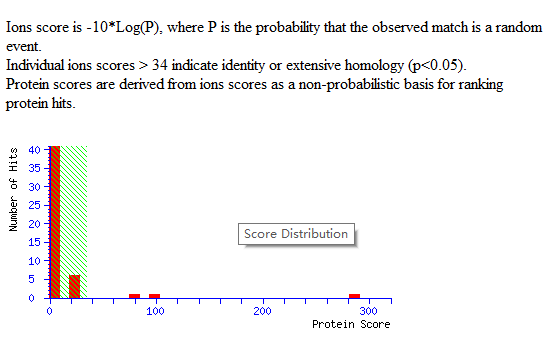
**

**Matched peptide sequences: shown in Bold Red:**

**
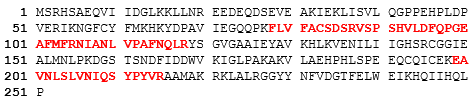
**

**Spot No.: 11**

**Protein name: ran-binding protein 1 homolog b-like [Hevea brasiliensis]**

**Peptide sequences:**

R.FDKEGNQWK.E(51);

K.ICANHLVVPSINVQEHHGNDK.S(12)

**Accession No.: ref|XP_021677540.1|**

**Mascot score: 64 Sequence coverage %: 12%**

**Calculated Mr:25725**

**Calculated pI:4.77**

**PFF Searched Score:
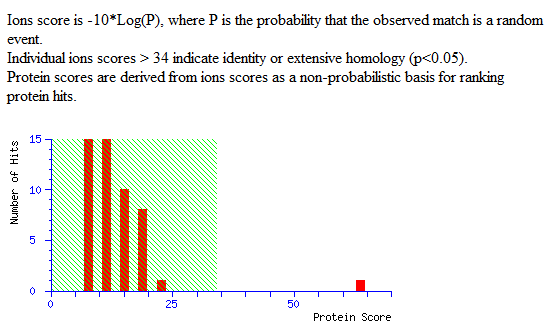
**

**Matched peptide sequences: shown in Bold Red:**

**
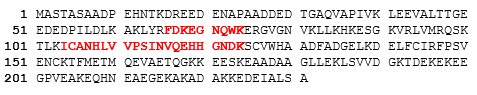
**

**Spot No.:12**

**Protein name: phosphoglycerate kinase [Hevea brasiliensis]**

**Peptide sequences:**

R.VDLNVPLDDNFNITDDTR.I;（127）

K.VILCTHLGRPK.G;（44）

K.YSLKPLVSR.L;（63）

K.LVAELSEGGVLLLENVR.F;（167）

K.LASLADVYVNDAFGTAHR.A;（147）

K.ELDYLVGAVANPK.K;（59）

K.AQGHSVGSSLVEEDKLDLATSLIEK.A（41）

**Accession No.: ref|XP_021651095.1|**

**Mascot score: 648**  **Sequence coverage %: 27%**

**Calculated Mr: 42600** **Calculated pI: 5.90**

**PFF Searched Score:**


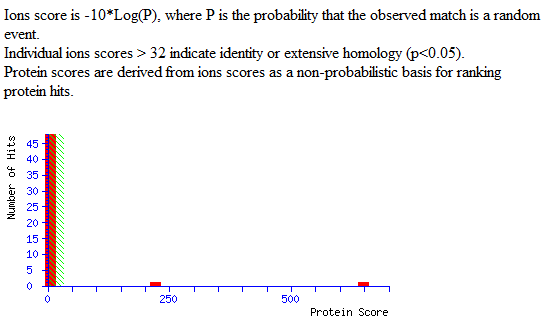


**Matched peptide sequences: shown in Bold Red:**


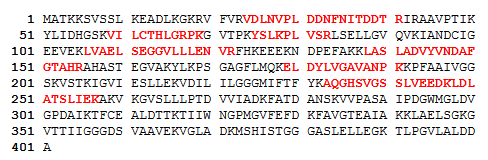


**Spot No.:13**

**Protein name: universal stress protein A-like protein [Hevea brasiliensis]**

**Peptide sequences:**

K.SICANHGIVAEILTEIGDPK.E(38);

K.HNVQLLVLGSHSR.G(123);

R.AFLGSVSNYCIHNAK.C(69)

**Accession No.: ref|XP_021671265.1|**

**Mascot score: 229 Sequence coverage %: 30%**

**Calculated Mr: 17427 Calculated pI: 6.05**

**PFF Searched Score:
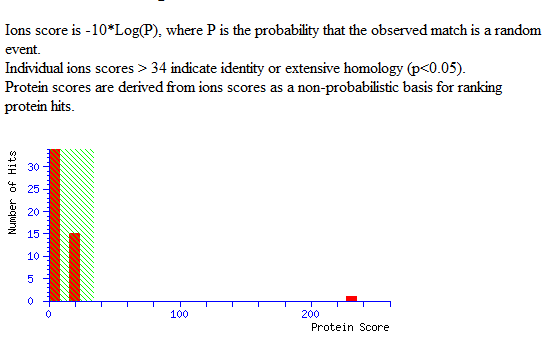
**

**Matched peptide sequences: shown in Bold Red:**

**
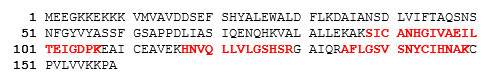
**

**Spot No.: 14**

**Protein name: pro-hevein [Hevea brasiliensis]**

**Peptide sequences:**

K.YGWTAFCGPVGAHGQPSCGK.C(79);

R.IVDQCSNGGLDLDVNVFR.Q(149)

**Accession No.: ref|XP_021650927.1|**

**Mascot score: 192 Sequence coverage %: 18%**

**Calculated Mr:23154 Calculated pI:5.89**

**PFF Searched Score:
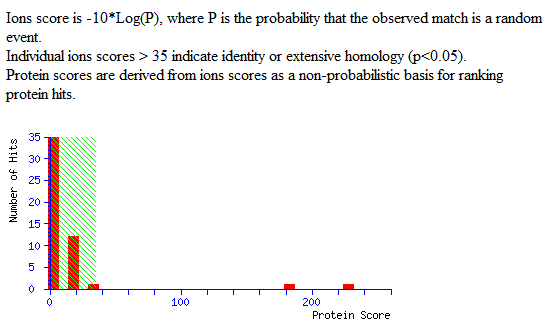
**

**Matched peptide sequences: shown in Bold Red:**

**
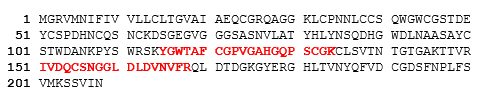
**

**Spot No.:15**

**Protein name: eukaryotic initiation factor 4A-14 [Hevea brasiliensis]**

**Peptide sequences:** K.MSELLSNEGQEFFTSYDEVYDSFDAMGLQENLLR.G(80);

R.GIYAYGFEKPSAIQQR.G(100);

K.TATFCSGILQQLDYGLVQCQALVLAPTR.E(106);

R.ILQAGVHVVVGTPGR.V(103);

R.QSLRPDYIR.M(43);

R.MFVLDEADEMLSR.G(80);

K.VQIGVFSATMPPEALEITR.K(140);

K.LETLCDLYETLAITQSVIFVNTR.R(145);

R.GIDVQQVSLVINYDLPTQPENYLHR.I(91);

R.KGVAINFVTR.D(57)

**Accession No.: ref|XP_021649957.1|**

**Mascot score: 943 Sequence coverage %: 46%**

**Calculated Mr: 47168 Calculated pI:5.49**

**PFF Searched Score:
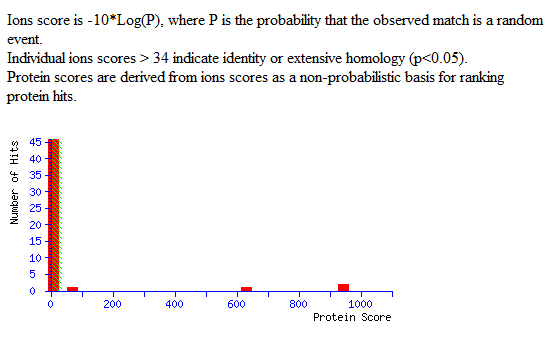
**

**Matched peptide sequences: shown in Bold Red:**

**
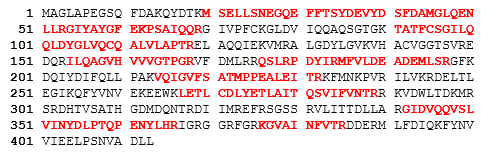
**

**Spot No.:16**

**Protein name: annexin D3-like [Hevea brasiliensis]**

**Peptide sequences:**

R.DTYQQLYNESLIDCLHSELSGDYR.K(73);

K.AVILWTYDPPER.D(34);

K.ELQVIVEIACANTPHHLQAVR.Q(85);

K.QLDHDDIVYVLSTR.N(118);

K.IRGEYFNIYK.T(37)

**Accession No.: ref|XP_021682791.1|**

**Mascot score: 327 Sequence coverage %: 25%**

**Calculated Mr:36631 Calculated pI:7.03**

**PFF Searched Score:
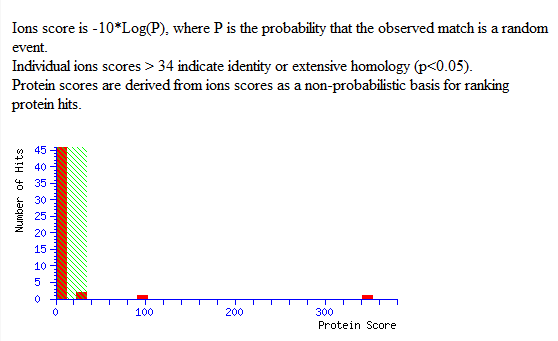
**

**Matched peptide sequences: shown in Bold Red:**

**
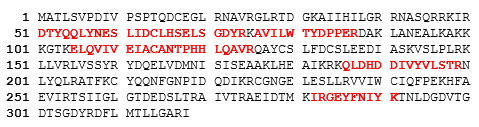
**

**Spot No.:17**

**Protein name: elongation factor 2-like [Hevea brasiliensis]**

**Peptide sequences:**

R.NGNEYLINLIDSPGHVDFSSEVTAALR.I(47);

R.ITDGALVVVDCVEGVCVQTETVLR.Q(99);

R.CFLELQVDGEEAYQTFQR.V(77);

K.YRVENLYEGPLDDPYATAIR.N(23);

R.VENLYEGPLDDPYATAIR.N(23);

R.GHVFEELQRPGTPLYNIK.A(32);

K.AYLPVVESFGFSGTLR.A(52)

**Accession No.: ref|XP_021676573.1|**

**Mascot score: 355 Sequence coverage %: 14%**

**Calculated Mr:94900 Calculated pI:5.80**

**PFF Searched Score:
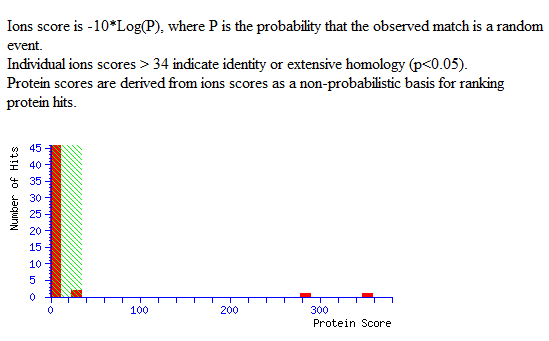
**

**Matched peptide sequences: shown in Bold Red:**

**
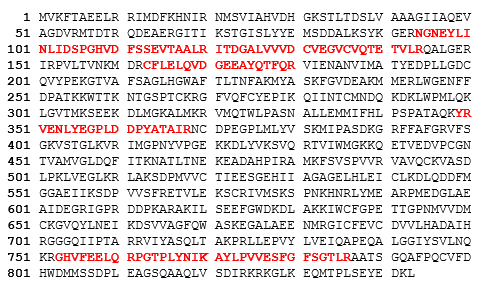
**

**Spot No.:18**

**Protein name: V-type proton ATPase subunit B2 isoform X1 [Hevea brasiliensis]**

**Peptide sequences:**

K.YQEIVNIR.L(33);

R.GYPGYMYTDLATIYER.A((Ions;

R.QIYPPINVLPSLSR.L(70);

K.FVAQGAYDTR.N(9);

K.TLDQYYSR.D(12)

**Accession No.: ref|XP_021683594.1|**

**Mascot score: 131 Sequence coverage %: 11%**

**Calculated Mr:55266 Calculated pI:4.92**

**PFF Searched Score:
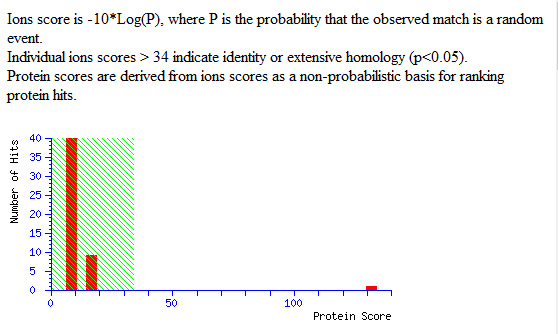
**

**Matched peptide sequences: shown in Bold Red:**

**
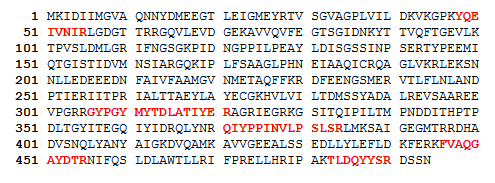
**

**Spot No.:19**

**Protein name: proteasome subunit beta type-1 isoform X1 [Hevea brasiliensis]**

**Peptide sequences:**

R.MSTGYSILTR.E(60);

R.HLIYQHQHNK.Q(72);

R.FFPYYAFNVLGGLDSEGK.G(84);

K.GCVYTYDAVGSYER.V(114);

R.VGYSAQGSGSTLIMPFLDNQLK.S(97);

K.SPSPLLLPAQDAVTPLSELEAVDLVK.T(96);

R.DIYTGDKLEIVILNADGIR.H(166);

R.HEYMELR.K(49)

**Accession No.: ref|XP_021668571.1|**

**Mascot score: 569 Sequence coverage %:56 %**

**Calculated Mr:24879 Calculated pI:6.20**

**PFF Searched Score:
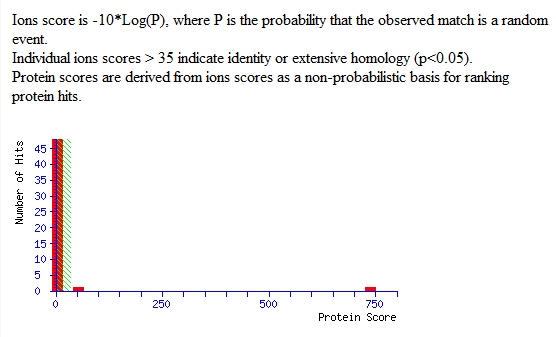
**

**Matched peptide sequences: shown in Bold Red:**

**
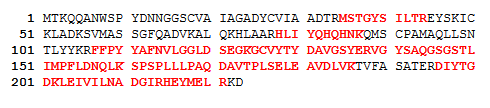
**

**Spot No.:20**

**Protein name: small rubber particle protein isoform X1 [Hevea brasiliensis]**

**Peptide sequences:** K.DISGPLKPGVDTIENVVK.T(82);

K.TVVTPVYYIPLEAVK.F(84);

K.FVDKTVDVSVTSLDGVVPPVIK.Q(27);

K.TVDVSVTSLDGVVPPVIK.Q(22);

K.QVSAQTYSVAQDAPR.I(138);

R.IVLDVASSVFNTGVQEGAK.A(152);

K.ALYANLEPK.A(53);

K.AEQYAVITWR.A(88);

R.ALNKLPLVPQVANVVVPTAVYFSEK.Y(55);

K.LPLVPQVANVVVPTAVYFSEK.Y(28);

K.LPLVPQVANVVVPTAVYFSEKYNDVVR.G(170);

R.VSSYLPLLPTEK.I(20)

**Accession No.: ref|XP_021653597.1|**

**Mascot score: 919 Sequence coverage %: 74%**

**Calculated Mr:22331 Calculated pI:4.80**

**PFF Searched Score:
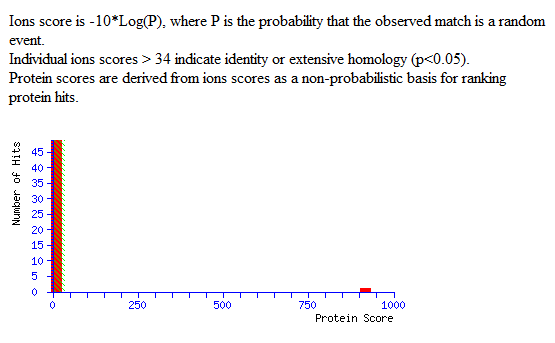
**

**Matched peptide sequences: shown in Bold Red:**

**
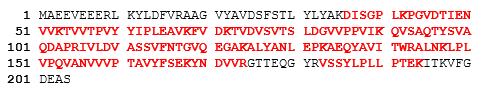
**

**Spot No.:21**

**Protein name: rubber elongation factor protein-like [Hevea brasiliensis]**

**Peptide sequences: K.YLDFVQAATVYAR.A(114);K.SVVRPVYNK.F(30);R.RVDAYVTVLDR.I(47)**

**Accession No.: ref|XP_021653600.1|**

**Mascot score: 191 Sequence coverage %: 18%**

**Calculated Mr:19612 Calculated pI:5.28**

**PFF Searched Score:
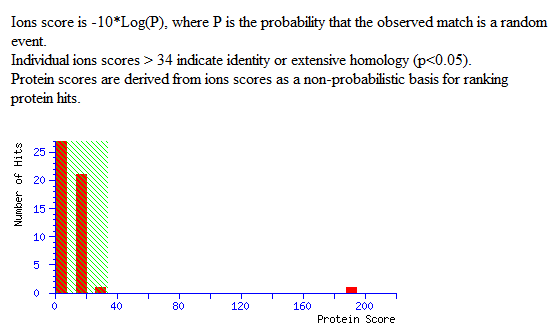
**

**Matched peptide sequences: shown in Bold Red:**

**
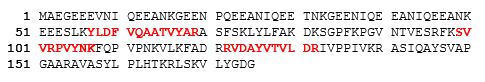
**

**Spot No.:22**

**Protein name: eukaryotic initiation factor 4A-14 [Hevea brasiliensis]**

**Peptide sequences: K.MSELLSNEGQEFFTSYDEVYDSFDAMGLQENLLR.G(67);R.GIYAYGFEKPSAIQQR.G(117);K.TATFCSGILQQLDYGLVQCQALVLAPTR.E(157);R.ILQAGVHVVVGTPGR.V(87);R.QSLRPDYIR.M(43);R.MFVLDEADEMLSR.G(108);R.GFKDQIYDIFQLLPAK.V(81);K.VQIGVFSATMPPEALEITR.K(149);K.QFYVNVEKEEWK.L(41);K.LETLCDLYETLAITQSVIFVNTR.R(154);R.GIDVQQVSLVINYDLPTQPENYLHR.I(118);R.KGVAINFVTR.D(55)**

**Accession No.: ref|XP_021649957.1|**

**Mascot score: 1140 Sequence coverage %: 53%**

**Calculated Mr:47168 Calculated pI:5.49**

**PFF Searched Score:
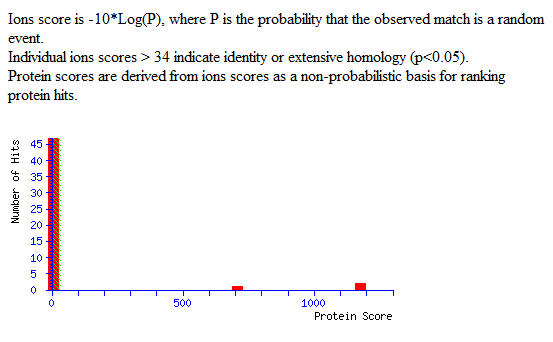
**

**Matched peptide sequences: shown in Bold Red:**

**
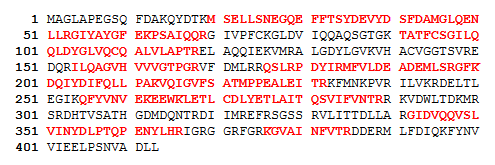
**

**Spot No.:23**

**Protein name: peroxiredoxin-2B-like [Hevea brasiliensis]**

**Peptide sequences: M.APIAAGDTVPEGTLAYFDDQDQLQQVSIHSLAAGK.K(78);K.HVPGFIER.A(57);K.FLADGSATYTHALGLELDLNEK.G(125);R.RFALLVDDLK.V(76);R.FALLVDDLK.V(62)**

**Accession No.: ref|XP_021675296.1|**

**Mascot score: 388 Sequence coverage %: 46%**

**Calculated Mr:17542 Calculated pI:5.55**

**PFF Searched Score:
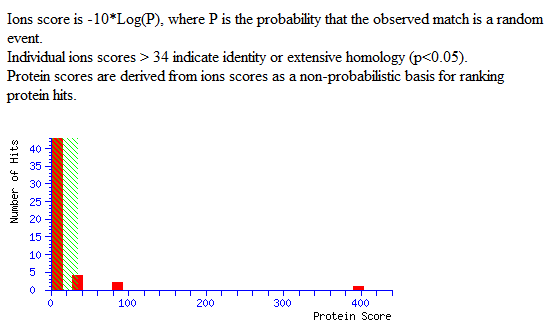
**

**Matched peptide sequences: shown in Bold Red:**

**
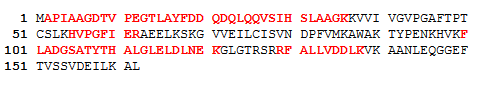
**

**Spot No.:24**

**Protein name: UDP-arabinopyranose mutase 3 isoform X1 [Hevea brasiliensis**

**Peptide sequences: R.NLDFLEMWR.P(51);K.VPEGFDYELHNR.N(70);K.NLLSPSTPFFFNTLYDPYR.N(29);R.EGVPTAVSHGLWLNIPDYDAPTQLVKPLER.N(28);K.GTLFPMCGMNLAFNR.E(48);R.ELIGPAMYFGLMGDGQPIGR.Y(53)**

**Accession No.: ref|XP_021674884.1|**

**Mascot score: 281 Sequence coverage %: 28%**

**Calculated Mr:41793 Calculated pI:5.76**

**PFF Searched Score:
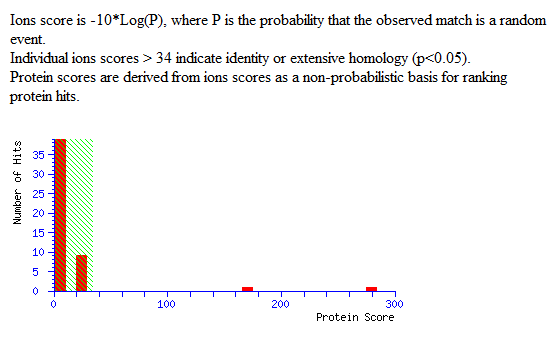
**

**Matched peptide sequences: shown in Bold Red:**

**
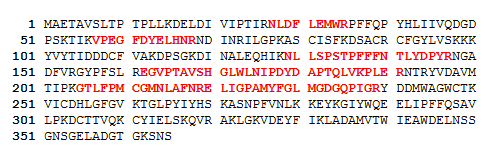
**

**Spot No.:25**

**Protein name: enolase 2 [Hevea brasiliensis]**

**Peptide sequences: R.AAVPSGASTGIYEALELR.D(127);K.HIANLAGNK.K(50);K.LAMQEFMILPVGASSFK.E((Ions;K.MGAEVYHHLK.S(30);K.MGAEVYHHLK.S((Ions;K.YGQDATNVGDEGGFAPNIQENK.E(95);K.VVIGMDVAASEFYGSDK.T(66);K.VQIVGDDLLVTNPK.R(99);K.VNQIGSVTESIEAVK.M(148);R.AGWGVMASHR.S(17);R.AGWGVMASHR.S((Ions;R.SGETEDTFIADLSVGLATGQIK.T(93);R.IEEELGAEAVYAGANFR.T(171)**

**Accession No.: ref|XP_021644856.1|**

**Mascot score: 897 Sequence coverage %: 38%**

**Calculated Mr:47993 Calculated pI:5.99**

**PFF Searched Score:
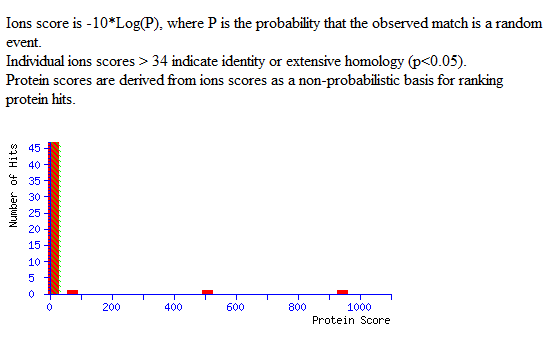
**

**Matched peptide sequences: shown in Bold Red:**

**
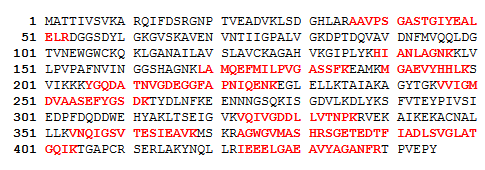
**

**Spot No.:26**

**Protein name: probable carboxylesterase 15 [Hevea brasiliensis]**

**Peptide sequences: K.FMADPVPAHQDFIDGVATR.D(112);R.LPAACDDGFSALLWLR.S(115);R.SSEPWLNDYADFNR.V(112);R.VFLIGDSSGGNLVHEVAAR.A(141);R.LAGGIPVHPGFVR.S(65)**

**Accession No.: ref|XP_021639445.1|**

**Mascot score: 552 Sequence coverage %: 24%**

**Calculated Mr:37612 Calculated pI:5.02**

**PFF Searched Score:
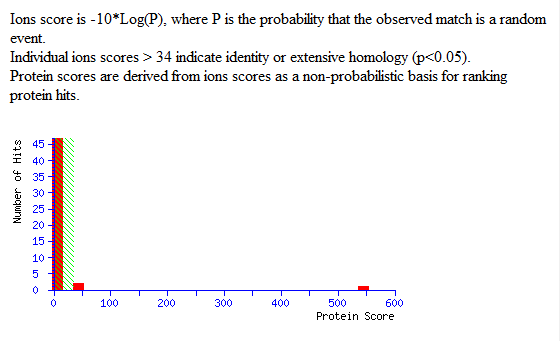
Matched peptide sequences: shown in Bold Red:**

**
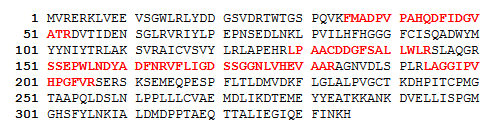
**

**Spot No.:27**

**Protein name: PITH domain-containing protein At3g04780 [Hevea brasiliensis]**

**Peptide sequences: R.SQVDLVDFVDWSGVECLNQNSSHSLPNALK.Q(82);K.QGYREDDGLNLESDADEQLLIYIPFTQVVK.L(21);K.LYSVVVQGPEEEGPK.T(79);R.SLTIFIEDNQSGSDITK.V(93)**

**Accession No.: ref|XP_021677274.1|**

**Mascot score: 278 Sequence coverage %: 52%**

**Calculated Mr:19557 Calculated pI:4.77**

**PFF Searched Score:
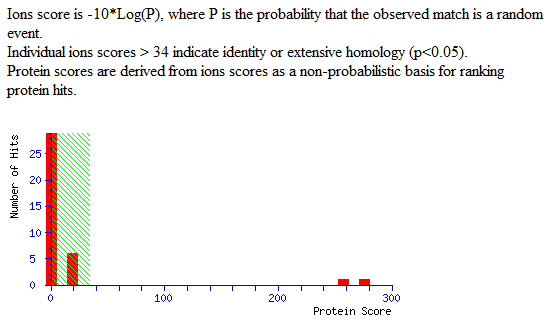
**

**Matched peptide sequences: shown in Bold Red:**

**
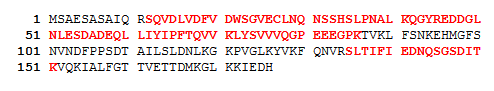
**

**Spot No.:28**

**Protein name: 14-3-3-like protein B [Hevea brasiliensis]**

**Peptide sequences: K.LVLGSTPSGELTVEER.N(16);K.AAQDIALADLAPTHPIR.L(58);K.DSTLIMQLLR.D(23)**

**Accession No.: ref|XP_021638817.1|**

**Mascot score: 98 Sequence coverage %: 16%**

**Calculated Mr:28505 Calculated pI: 4.71**

**PFF Searched Score:
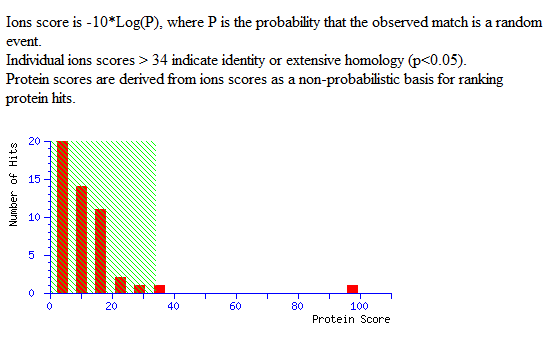
**

**Matched peptide sequences: shown in Bold Red:**

**
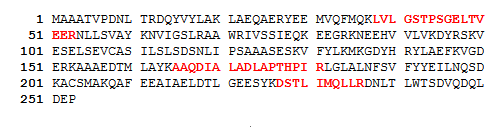
**

**Spot No.:29**

**Protein name: probable nucleoredoxin 1 [Hevea brasiliensis]**

**Peptide sequences: R.HFTPNLVEVYEK.L(63);K.GDFEVVFISSDR.D(31);K.GDFEVVFISSDRDDESFNGYFSK.M(86);K.MPWLAIPFSDQEIR.K((Ions;R.DYGAEGYPFTPER.L(87);R.LDYFRQEEENAK.K(21);R.ALPTLVIIGQDGK.T(14);K.TLNPNVAELIEDHGIEAYPFTPEK.L(13);R.LEAQTLESVLVHGDKDFVIEK.S(66);K.LIEAYHEIK.A(44);R.EHLAAYGADAYPFTEDHLK.Q(134);K.LKHELHSQHELTR.T(86);K.HELHSQHELTR.T(56);R.QCDFDLHPK.C(45)**

**Accession No.: ref|XP_021649126.1|**

**Mascot score: 737 Sequence coverage %: 32%**

**Calculated Mr:65399 Calculated pI:5.62**

**PFF Searched Score:
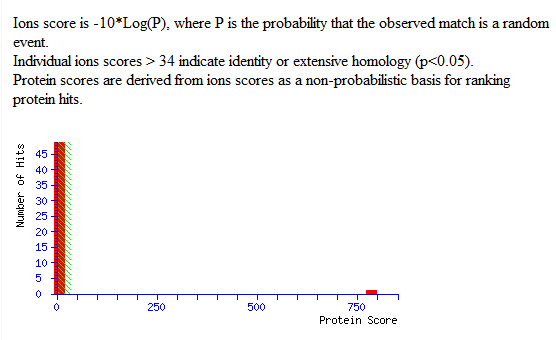
**

**Matched peptide sequences: shown in Bold Red:**

**
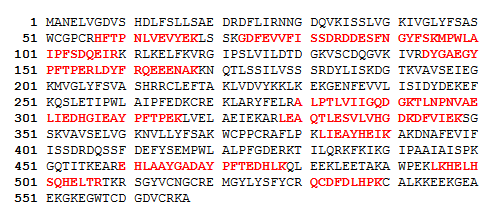
**

**Spot No.:30**

**Protein name: protein EARLY-RESPONSIVE TO DEHYDRATION 7, chloroplastic-like [Hevea brasiliensis]**

**Peptide sequences: K.LDESHYFFNLR.V(94);K.GQEALLKEFDNILESYSSFTVQEVNER.G(126);K.EFDNILESYSSFTVQEVNER.G(171);K.ELEECSAAYWTVLAPNVEDYSGSVSR.M(154);K.GILWCGDVTVDR.L(79)**

**Accession No.: ref|XP_021658906.1|**

**Mascot score: 627 Sequence coverage %: 17%**

**Calculated Mr:48761 Calculated pI:5.11**

**PFF Searched Score:
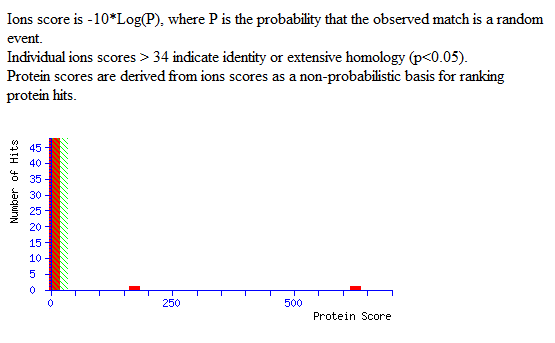
**

**Matched peptide sequences: shown in Bold Red:**

**
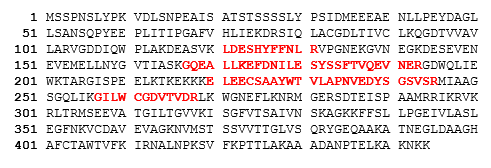
**

**Spot No.:31**

**Protein name: universal stress protein A-like protein [Hevea brasiliensis]**

**Peptide sequences: R.IMIAVNESTIK.G(34);K.GAFDWTLQK.I(51);R.GLHLLEYFVNR.C(85);R.CHQIGVACEAWIK.S(99);K.EVICHEVK.R(20);K.EVICHEVKR.V(53);K.RVLPDFLVVGCR.G(16);R.VLPDFLVVGCR.G(92);R.VFVGTVSEFCQK.H(78)**

**Accession No.: ref|XP_021636308.1|**

**Mascot score: 531 Sequence coverage %: 43%**

**Calculated Mr:20167 Calculated pI:5.87**

**PFF Searched Score:
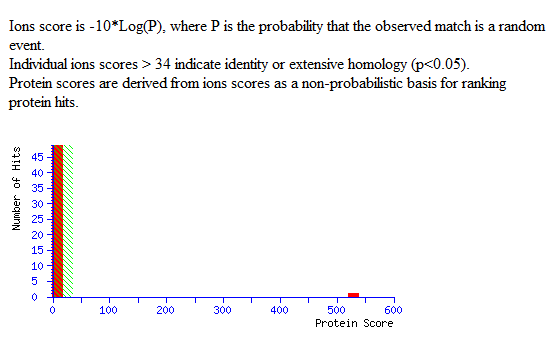
**

**Matched peptide sequences: shown in Bold Red:**

**
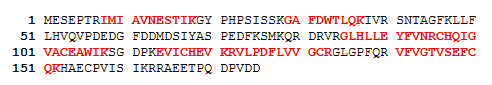
**

**Spot No.:32**

**Protein name: T-complex protein 1 subunit zeta 1 [Hevea brasiliensis]**

**Peptide sequences:**

**R.YIDEGMHPR.V; R.YIDEGMHPR.V((Ions; R.HKFDVDTR.L; R.LVEGLVLDHGSR.H;K.SEINAGFFYSNAEQR.E; K.GIDPPSLDLLAR.A; K.GPNDHTIAQIK.D; K.NTIEDEAVVLGAGAFELAAR.K**

**Accession No.: ref|XP_021643906.1|**

**Mascot score: 324 Sequence coverage %:16%**

**Calculated Mr:59367 Calculated pI:5.87**

**PFF Searched Score:**


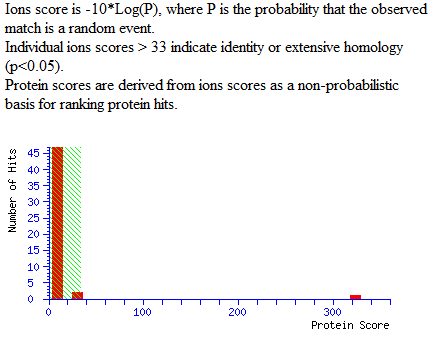


**Matched peptide sequences: shown in Bold Red:**


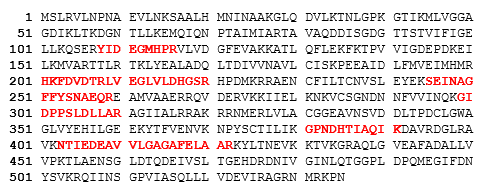


**Spot No.:33**

**Protein name:** **rubber elongation factor protein [Hevea brasiliensis]**

**Peptide sequences: K.DKSGPLQPGVDIIEGPVK.N(129);K.NVAVPLYNR.F(39);K.FVDSTVVASVTIIDR.S(143);K.DASIQVVSAIR.A(103);R.SLASSLPGQTK.I(38)**

**Accession No.: ref|XP_021653602.1|**

**Mascot score: 451 Sequence coverage %:46 %**

**Calculated Mr:14713 Calculated pI:5.04**

**PFF Searched Score:**


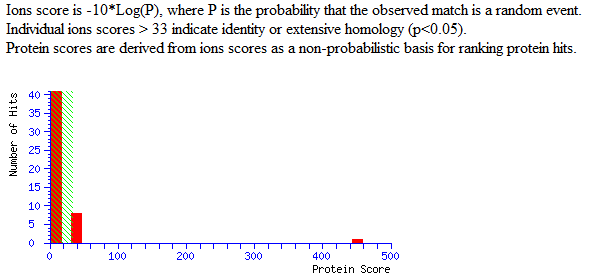


**Matched peptide sequences: shown in Bold Red:**


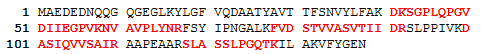


**Spot No.:34**

**Protein name: rubber elongation factor protein [Hevea brasiliensis]**

**Peptide sequences: K.DKSGPLQPGVDIIEGPVK.N(128);K.NVAVPLYNR.F(75);K.FVDSTVVASVTIIDR.S(142);K.DASIQVVSAIR.A(102);R.SLASSLPGQTK.I(38)**

**Accession No.: ref|XP_021653602.1|**

**Mascot score: 372 Sequence coverage %: 38%**

**Calculated Mr:14713 Calculated pI:5.04**

**PFF Searched Score:
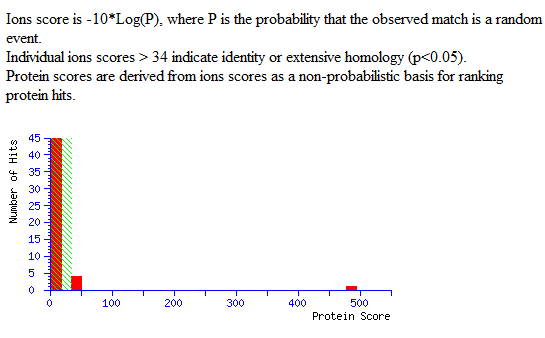
Matched peptide sequences: shown in Bold Red:**

**
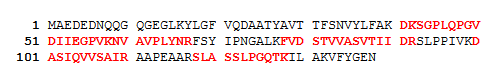
**

**Spot No.:35**

**Protein name:** **rubber elongation factor protein [Hevea brasiliensis]**

**Peptide sequences: K.DKSGPLQPGVDIIEGPVK.N(132);K.NVAVPLYNR.F(38);K.FVDSTVVASVTIIDR.S(146);K.DASIQVVSAIR.A(114);R.SLASSLPGQTK.I(3)**

**Accession No.:** **ref|XP_021653602.1|**

**Mascot score: 433 Sequence coverage %: 46%**

**Calculated Mr:14713 Calculated pI:5.04**

**PFF Searched Score:**


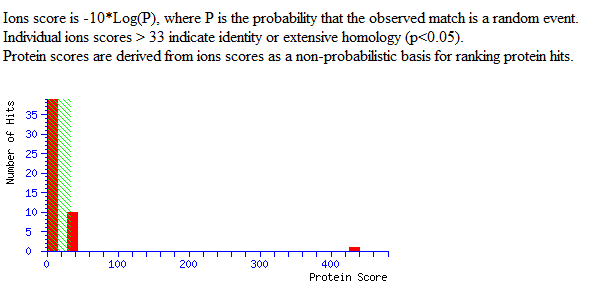


**Matched peptide sequences: shown in Bold Red:**


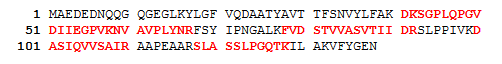


**Spot No.:36**

**Protein name:** **rubber elongation factor protein [Hevea brasiliensis]**

**Peptide sequences: K.YLGFVQDAATYAVTTFSNVYLFAK.D(89);K.DKSGPLQPGVDIIEGPVK.N(147);K.NVAVPLYNR.F(41);K.FVDSTVVASVTIIDR.S(119);K.DASIQVVSAIR.A(110);R.SLASSLPGQTK.I(53)**

**Accession No.:** **ref|XP_021653602.1|**

**Mascot score: 560 Sequence coverage %: 63%**

**Calculated Mr:14713 Calculated pI:5.04**

**PFF Searched Score:**


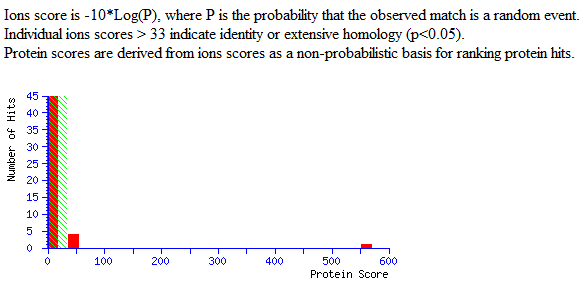


**Matched peptide sequences: shown in Bold Red:**


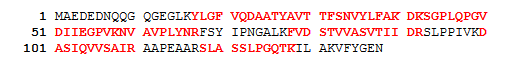


**Spot No.:37**

**Protein name:tubulin alpha-3 chain [Hevea brasiliensis]**

**Peptide sequences:**

**R.AVFVDLEPTVIDEVR.T(104);R.QLFHPEQLISGK.E(42);K.LADNCTGLQGFLVFNAVGGGTGSGLGSLLLER.L(81);R.LISQIISSLTTSLR.F(51);R.FDGAINVDVTEFQTNLVPYPR.I(98);R.TVQFVDWCPTGFK.C(47);K.CGINYQPPTVVPGGDLAR.V(106);R.AFVHWYVGEGMEEGEFSEAR.E(80)**

**Accession No.:ref|XP_021644460.1|**

**Mascot score:607 Sequence coverage %:32%**

**Calculated Mr:50244 Calculated pI:5.00**

**PFF Searched Score:**

**
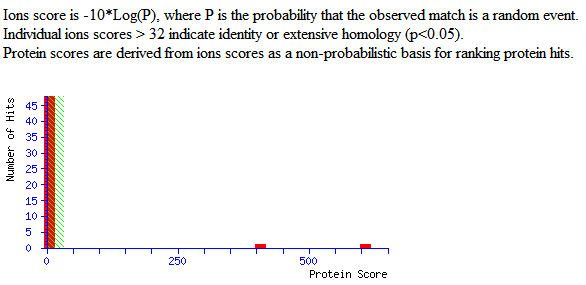
**

**Matched peptide sequences: shown in Bold Red:**

**
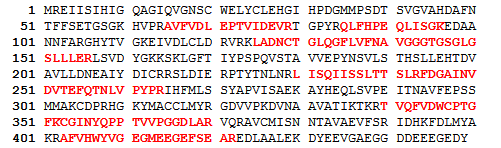
**

**Spot No.: 38**

**Protein name:** **cysteine synthase isoform X1 [Hevea brasiliensis]**

**Peptide sequences: K.TPLVYLNHVVDGCVAR.I(81);K.LIITMPASMSLER.R(46);K.TPNAYVLQQFENPSNPK.V(85);K.LYGVEPVESAVLSGGKPGPHK.I(86);K.EGLLVGISSGAAAAAAIR.I(122);K.LIVVVFPSFGER.Y(74);R.YLSSVLFESVK.R(85)**

**Accession No.:** **ref|XP_021677329.1|**

**Mascot score: 581 Sequence coverage %:30%**

**Calculated Mr:37572 Calculated pI:6.28**

**PFF Searched Score:**


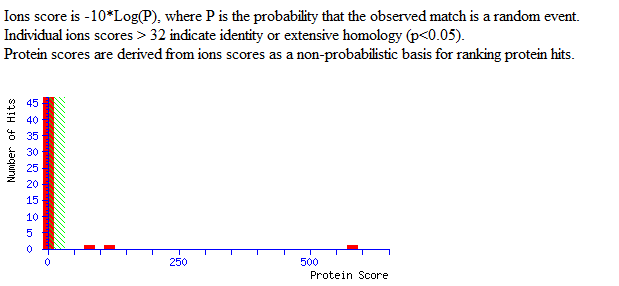


**Matched peptide sequences: shown in Bold Red:**


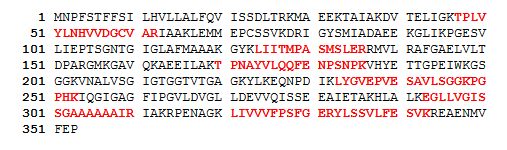


**Spot No.:39**

**Protein name: REF/SRPP-like protein At3g05500[Hevea brasiliensis]**

**Peptide sequences: K.AGPLKPGVETVEGTVK.S(58);K.AEQCAVTAWR.R(60);R.VSSYLPLVPTER.I(73)**

**Accession No.: ref|XP_021662186.1|**

**Mascot score: 191 Sequence coverage %:15%**

**Calculated Mr:27100 Calculated pI:6.36**

**PFF Searched Score:**


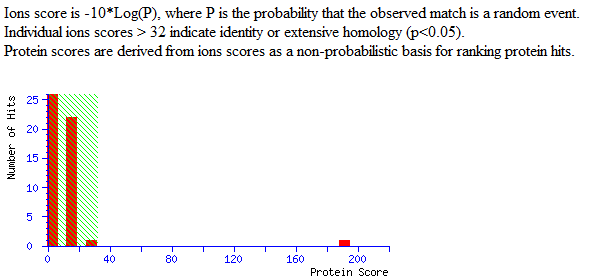


**Matched peptide sequences: shown in Bold Red:**


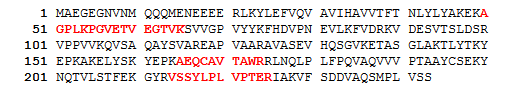


**Spot No.:40**

**Protein name:** **leucine aminopeptidase 1-like isoform X2 [Hevea brasiliensis]**

**Peptide sequences: K.KLDAHLGGLLSEASSEEDFTGK.A(64);K.LNTASAIASGTVLGIYEDNR.Y(79);K.LNTASAIASGTVLGIYEDNRYK.S(64);R.LTLADALVYACNQGVEK.I(46);K.VQWMHIDLAGPVWNEK.K(76)**

**Accession No.:** **ref|XP_021677329.1|**

**Mascot score: 328 Sequence coverage %:13%**

**Calculated Mr:61619 Calculated pI:6.44**

**PFF Searched Score:**


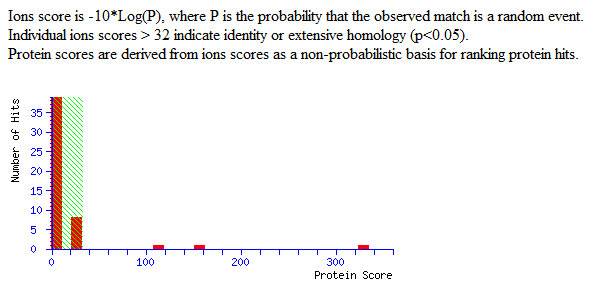


**Matched peptide sequences: shown in Bold Red:**


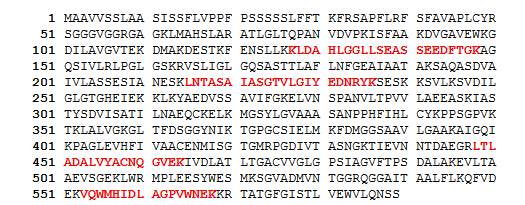


**Spot No.:41**

**Protein name:** **chlorophyllase-1, chloroplastic-like [Hevea brasiliensis]**

**Peptide sequences: K.EIEFAAEVGNWLLSGLQSVLPEK.V(125);R.GGNIAFALALGYSK.T(87);K.ISALVGLDPVGR.V(105)**

**Accession No.:** **ref|XP_021690613.1|**

**Mascot score: 316 Sequence coverage %:14%**

**Calculated Mr:37785 Calculated pI:6.30**

**PFF Searched Score:**


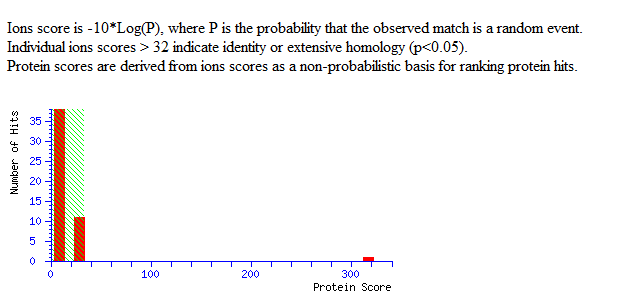


**Matched peptide sequences: shown in Bold Red:**


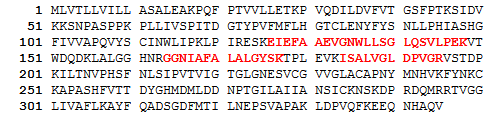


**Spot No.:42**

**Protein name:** **nucleoside diphosphate kinase 1-like [Hevea brasiliensis]**

**Peptide sequences: K.LMSVERPFAEK.H(12);K.LMSVERPFAEK.H((Ions;K.IIGATKPSESDPGTIR.G(105);R.NVIHGSDSVENAK.K(87);R.NVIHGSDSVENAKK.E(60)**

**Accession No.:** **ref|XP_021678142.1|**

**Mascot score: 271 Sequence coverage %:27%**

**Calculated Mr:16463 Calculated pI:5.96**

**PFF Searched Score:**


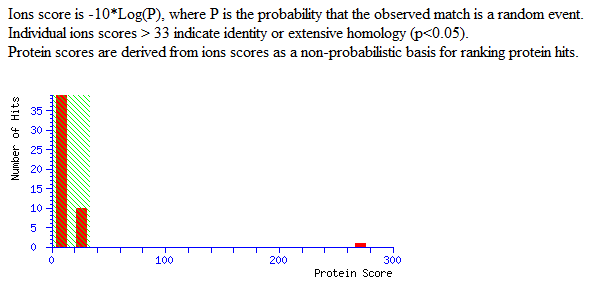


**Matched peptide sequences: shown in Bold Red:**


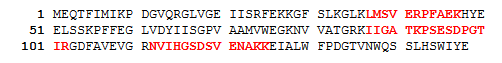


**Spot No.:43**

**Protein name:** **phosphoglycerate kinase, cytosolic-like [Hevea brasiliensis]**

**Peptide sequences: R.VDLNVPLDDNFNITDDTR.I(135);K.VILCTHLGRPK.G(40);K.YSLKPLVPR.L(22);K.LVAELSDGGVLLLENVR.F(97);K.YLKPSVAGFLMQK.E((Ions;K.ELDYLVGAVANPK.K(60);K.AQGYSVGSSLVEEDKLDLATSLVEK.A(66);K.GVSLLLPTDVVIADK.F(28);K.VVPASAIPDGWMGLDVGPDAIK.T((Ions**

**Accession No.:** **ref|XP_021681983.1|**

**Mascot score: 489 Sequence coverage %:35%**

**Calculated Mr:42438 Calculated pI:5.67**

**PFF Searched Score:**


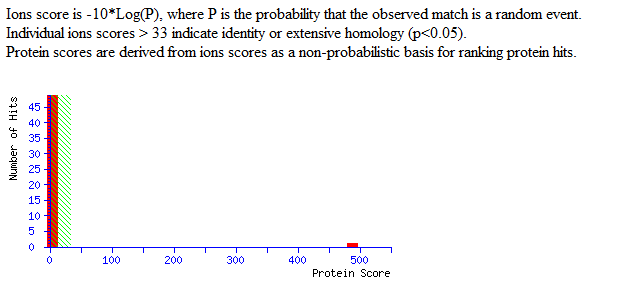


**Matched peptide sequences: shown in Bold Red:**


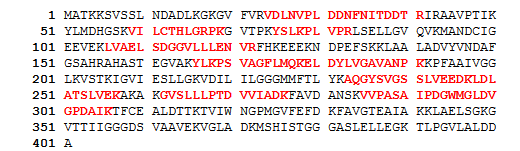


**Spot No.:44**

**Protein name: endo-1,3;1,4-beta-D-glucanase-like [Hevea brasiliensis]**

**Peptide sequences:**

**K.LAIILISDVYGYEAPNLR.K(110);K.QYEEVLAAKPEVDR.H(100);K.VAHGWTVR.Y(49)**

**Accession No.:ref|XP_021688919.1|**

**Mascot score:259 Sequence coverage %:16%**

**Calculated Mr:26106 Calculated pI:5.81**

**PFF Searched Score:**

**
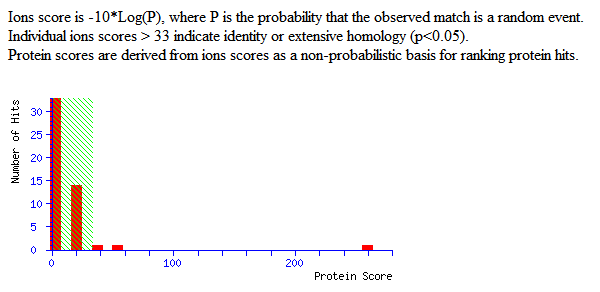
**

**Matched peptide sequences: shown in Bold Red:**

**
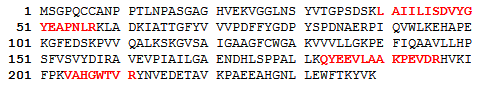
**

**Spot No.:45**

**Protein name:** **subtilisin-like protease SBT1.4 [Hevea brasiliensis]**

**Peptide sequences: R.EFPADVVLGDGR.I(85);K.LSQFPTATIVFHGTVIGTSPPAPK.V(34);R.VEFNIISGTSMSCPHVSGIAALLR.K(21);K.AYPNWSPAAIK.S(53);R.IAVFVGGTATLDVCDR.K(96);R.IAVFVGGTATLDVCDRK.L(15)**

**Accession No.:** **ref|XP_021638895.1|**

**Mascot score: 304 Sequence coverage %:11%**

**Calculated Mr:82577 Calculated pI:6.66**

**PFF Searched Score:**


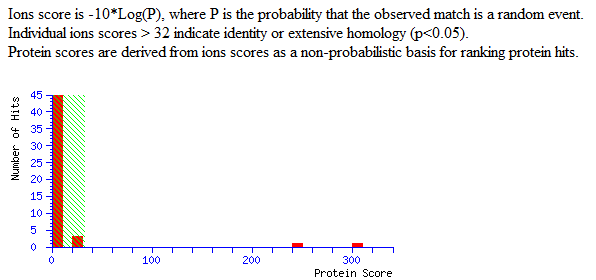


**Matched peptide sequences: shown in Bold Red:**


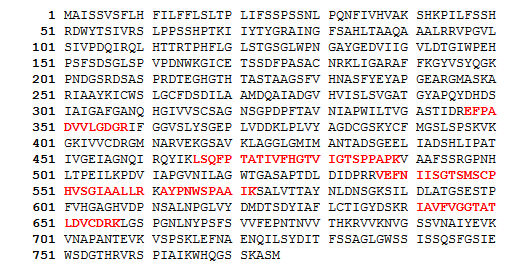


**Spot No.:46**

**Protein name:** **glutamine synthetase cytosolic isozyme 2-like [Hevea brasiliensis]**

**Peptide sequences: K.WNYDGSSTGQAPGEDSEVILYPQAIFR.D(106);R.ITEIAGVVLSFDPK.P(27);R.HKEHIAAYGEGNER.R(44);K.EHIAAYGEGNER.R(44);R.HETADINTFLWGVANR.G(73)**

**Accession No.:** **ref|XP_021690409.1|**

**Mascot score: 294 Sequence coverage %:19%**

**Calculated Mr:39399 Calculated pI:5.64**

**PFF Searched Score:**


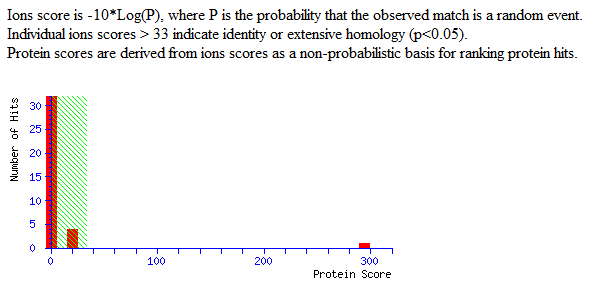


**Matched peptide sequences: shown in Bold Red:**


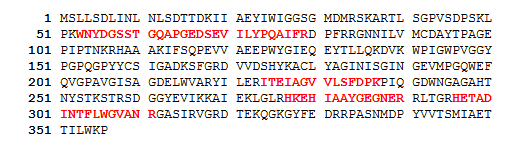


**Spot No.:47**

**Protein name:** **phosphomevalonate kinase, peroxisomal-like isoform X1 [Hevea brasiliensis]**

**Peptide sequences: K.LLLQGLDITILGTNDFYSYR.N(113);K.VGSGFDVSSAVYGSHR.Y(148);K.WIEQATEPSR.E(51);K.AWSSLNVLALLVR.E(41)**

**Accession No.:** **ref|XP_021651365.1|**

**Mascot score: 351 Sequence coverage %:11%**

**Calculated Mr:54477 Calculated pI:6.05**

**PFF Searched Score:**


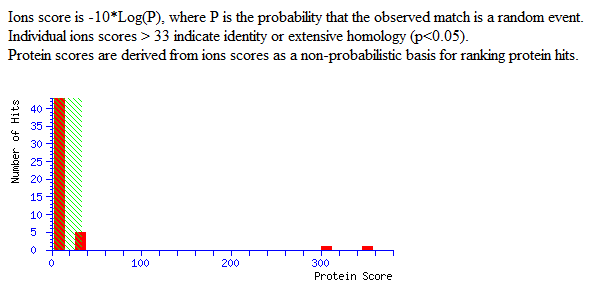


**Matched peptide sequences: shown in Bold Red:**


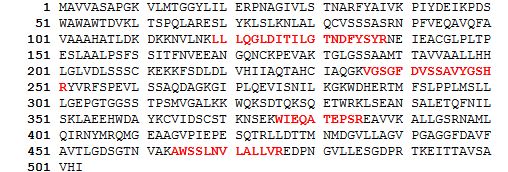


**Spot No.:48**

**Protein name:** **small rubber particle protein isoform X1 [Hevea brasiliensis]**

**Peptide sequences: K.DISGPLKPGVDTIENVVK.T(115);K.TVVTPVYYIPLEAVK.F(68);K.FVDKTVDVSVTSLDGVVPPVIK.Q(18);K.QVSAQTYSVAQDAPR.I(142);R.IVLDVASSVFNTGVQEGAK.A(112);K.ALYANLEPK.A(49);K.AEQYAVITWR.A(88);R.ALNKLPLVPQVANVVVPTAVYFSEK.Y(84);K.LPLVPQVANVVVPTAVYFSEK.Y(16);K.LPLVPQVANVVVPTAVYFSEKYNDVVR.G(17);R.VSSYLPLLPTEK.I(29)**

**Accession No.:** **ref|XP_021653597.1|**

**Mascot score: 739 Sequence coverage %:74%**

**Calculated Mr:22331 Calculated pI:4.80**

**PFF Searched Score:**


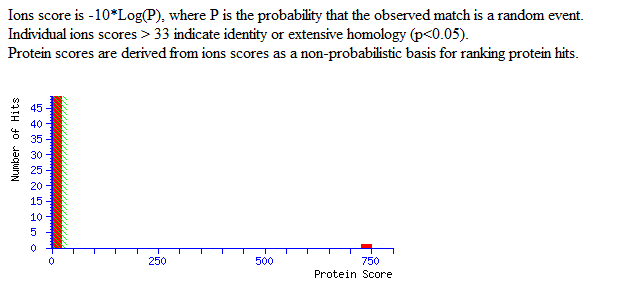


**Matched peptide sequences: shown in Bold Red:**


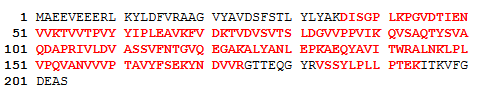


**Spot No.:49**

**Protein name:** **rubber elongation factor protein-like [Hevea brasiliensis]**

**Peptide sequences: K.SGPFKPGVNTVESR.F(109);R.RVDAYVTVLDR.I(80);R.VDAYVTVLDR.I(82)**

**Accession No.:** **ref|XP_021653600.1|**

**Mascot score: 270 Sequence coverage %:14%**

**Calculated Mr:19612 Calculated pI:5.28**

**PFF Searched Score:**


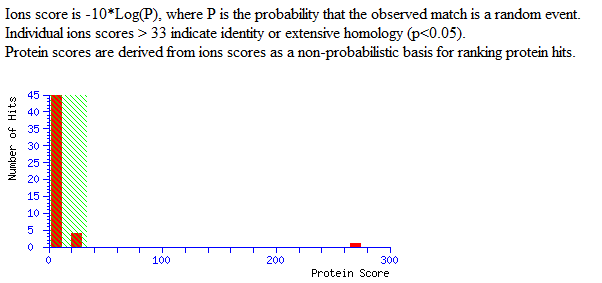


**Matched peptide sequences: shown in Bold Red:**


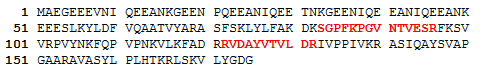


**Spot No.:50**

**Protein name:** **ubiquitin-conjugating enzyme E2 variant 1C isoform X2 [Hevea brasiliensis]**

**Peptide sequences: K.GIGDGTVSYGMDDGDDIYMR.S(126);R.SWTGTIIGPHNTVHEGR.I(99);K.KFGLLANWQR.E(44);K.FGLLANWQR.E(44);R.EYTMEDILTQLK.K(43);R.EYTMEDILTQLKK.E(56)**

**Accession No.:** **ref|XP_021636663.1|**

**Mascot score:412 Sequence coverage %:41%**

**Calculated Mr:16696 Calculated pI:6.20**

**PFF Searched Score:**


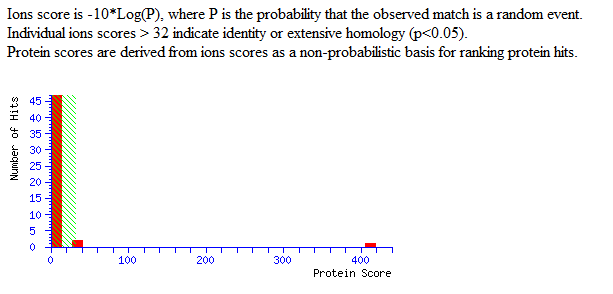


**Matched peptide sequences: shown in Bold Red:**


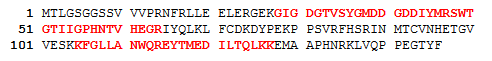


**Spot No.:51**

**Protein name:** **enolase 1 [Hevea brasiliensis]**

**Peptide sequences: R.AAVPSGASTGIYEALELR.D(140);K.SFVAEYPIVSIEDPFDQDDWAHYAK.L(43);R.AGWGVMASHR.S(34);R.SGETEDTFIADLSVGLATGQIK.T(60);R.IEEELGSEAVYAGANFR.K(144)**

**Accession No.:** **ref|XP_021678789.1|**

**Mascot score:419 Sequence coverage %:20%**

**Calculated Mr:48029 Calculated pI:5.57**

**PFF Searched Score:**

**Matched peptide sequences: shown in Bold Red:**

**Spot No.:52**

**Protein name:** **desiccation-related protein At2g46140 isoform X1 [Hevea brasiliensis]**

**Peptide sequences: K.NPNPIPIPLIDINYLIESDGR.K(138);K.NPNPIPIPLIDINYLIESDGRK.L(73);K.IPVTLIYDDIR.S(67);R.STYNDIKPGSIIPYR.I(93);R.IKVDLIVDVPVFGR.L(60);K.TGEIPIPYKPDIDLEK.I(54)**

**Accession No.:** **ref|XP_021642239.1|**

**Mascot score:486 Sequence coverage %:24%**

**Calculated Mr:35055 Calculated pI:4.71**

**PFF Searched Score:**

**Matched peptide sequences: shown in Bold Red:**

**Spot No.:53**

**Protein name:** **pleckstrin homology domain-containing protein 1-like [Hevea brasiliensis]**

**Peptide sequences:**

**R.AWTGQDPNPDDYSNIQFWSNPER.S(109)**

**Accession No.:** **ref|XP_021659098.1|**

**Mascot score:109 Sequence coverage %:16%**

**Calculated Mr:16920 Calculated pI:5.87**

**PFF Searched Score:**

**Matched peptide sequences: shown in Bold Red:**

**Spot No.:54**

**Protein name:** **ankyrin repeat domain-containing protein 2A-like isoform X1 [Hevea brasiliensis]**

**Peptide sequences: K.ELAEQISKDPAFNQMAEQLHK.T(52);R.YWNDKEVLQK.L(76);K.NALASGANKDEEDSEGR.T(41);R.TALHFACGYGEVK.C(92);K.NTALHYAAGYGR.K(99)**

**Accession No.:** **ref|XP_021646829.1|**

**Mascot score:360 Sequence coverage %:20%**

**Calculated Mr:37639 Calculated pI:4.50**

**PFF Searched Score:**

**Matched peptide sequences: shown in Bold Red:**

**Spot No.:55**

**Protein name:** **pro-hevein [Hevea brasiliensis]**

**Peptide sequences: K.YGWTAFCGPVGAHGQPSCGK.C(64);R.IVDQCSNGGLDLDVNVFR.Q(97)**

**Accession No.:** **ref|XP_021650927.1|**

**Mascot score:161 Sequence coverage %:18%**

**Calculated Mr:23154 Calculated pI:5.89**

**PFF Searched Score:**

**Matched peptide sequences: shown in Bold Red:**

**Spot No.:56**

**Protein name:** **REF/SRPP-like protein At3g05500 [Hevea brasiliensis]**

**Peptide sequences: K.FHDVPNEVLK.F(12);R.AVASEVHQSGVK.E(33);R.VSSYLPLVPTER.I(49)**

**Accession No.:** **ref|XP_021662186.1|**

**Mascot score:94 Sequence coverage %:13%**

**Calculated Mr:27100 Calculated pI:6.36**

**PFF Searched Score:**

**Matched peptide sequences: shown in Bold Red:**

**Spot No.:57**

**Protein name:** **caffeic acid 3-O-methyltransferase-like [Hevea brasiliensis]**

**Peptide sequences: K.SALELNVIDIISTAGNSGASLSAPEIAQR.I(216);K.NPEAPVLLDR.M(57);K.NGSGSAAPLLLLHHDEVFMK.S(32);K.SWYHLNEAILEGGTPFNR.A(119);R.AYGMNQFEYPGTDQR.F(87);R.AYGMNQFEYPGTDQR.F((Ions;K.WVLHDWNDDLCLK.L(72);R.TRQEFEALASK.S(67)**

**Accession No.:** **ref|XP_021638873.1|**

**Mascot score:659 Sequence coverage %:31%**

**Calculated Mr:40913 Calculated pI:5.54**

**PFF Searched Score:**

**Matched peptide sequences: shown in Bold Red:**

**Spot No.:58**

**Protein name:** **26S proteasome regulatory subunit 6B homolog [Hevea brasiliensis]**

**Peptide sequences: R.EAVELPLTHHELYK.Q(4);K.AVANHTTAAFIR.V(49);K.ENAPAIIFIDEVDAIATAR.F(5);R.ADTLDPALLRPGR.L(7);K.KPDTDFEFYK.-(20)**

**Accession No.:** **ref|XP_021666129.1|**

**Mascot score:85 Sequence coverage %:16%**

**Calculated Mr:47304 Calculated pI:5.42**

**PFF Searched Score:**

**Matched peptide sequences: shown in Bold Red:**

**Spot No.:59**

**Protein name:** **NADPH-dependent aldehyde reductase 1,chloroplastic [Hevea brasiliensis]**

**Peptide sequences: R.FPPQTQDR.Q(1);K.EYLMHPLPEFLNPQYKPSNK.L(12);K.EYLMHPLPEFLNPQYKPSNK.L((Ions;K.VALVTGGDSGIGR.A(59);K.GQEDMDTDHTLK.I(25);K.DPIAIPTDVGFEENCR.S(116);R.TNIFGQFFMTR.Y(38);R.TNIFGQFFMTR.Y((Ions;R.VNAVAPGPVWTPLQPASLPAEK.I(51);K.IAALGSEVPMDR.A(23);K.IAALGSEVPMDR.A((Ions**

**Accession No.:** **ref|XP_021670828.1|**

**Mascot score:373 Sequence coverage %:38%**

**Calculated Mr:32309 Calculated pI:5.29**

**PFF Searched Score:**

**Matched peptide sequences: shown in Bold Red:**

**Spot No.:60**

**Protein name:** **nucleoside diphosphate kinase B [Hevea brasiliensis]**

**Peptide sequences: R.GLVGEIIGR.F(72);R.KIIGATNPADSAPGTIR.G(63);K.IIGATNPADSAPGTIR.G(89);R.GDYAIDIGR.N(71);R.NVIHGSDSVESAR.K(88)**

**Accession No.:** **ref|XP_021679143.1|**

**Mascot score:383 Sequence coverage %:32%**

**Calculated Mr:16338 Calculated pI:6.85**

**PFF Searched Score:**

**Matched peptide sequences: shown in Bold Red:**

**Spot No.:61**

**Protein name:** **triosephosphate isomerase,cytosolic[Heveabrasiliensis]**

**Peptide sequences: R.KFFVGGNWK.C(28);K.FFVGGNWK.C(73);K.DLLRPDFQVAAQNCWVR.K(90);R.SLLNESNEFVGDK.V(86);K.VIACIGETLEQR.E(91);K.VATPAQAQEVHLELR.K(130);K.VATPAQAQEVHLELRK.W(8);R.KWLHDNVCAEVAASTR.I(56);K.WLHDNVCAEVAASTR.I(128)**

**Accession No.:** **ref|XP_021646026.1|**

**Mascot score:688 Sequence coverage %:32%**

**Calculated Mr:27571 Calculated pI:5.90**

**PFF Searched Score:**

**Matched peptide sequences: shown in Bold Red:**

**Spot No.:62**

**Protein name:** **uncharacterized protein LOC110659188 [Hevea brasiliensis]**

**Peptide sequences: K.FQELDRDGDGK.L(16);K.EQPVIVAHSENTFDGSGIR.R(114);K.MSQEYLR.V(17)**

**Accession No.:** **ref|XP_021672744.1|**

**Mascot score:146 Sequence coverage %:10%**

**Calculated Mr:40238 Calculated pI:4.52**

**PFF Searched Score:**

**Matched peptide sequences: shown in Bold Red:**

**Spot No.:63**

**Protein name:** **rubber elongation factor protein [Hevea brasiliensis]**

**Peptide sequences: K.DKSGPLQPGVDIIEGPVK.N(142);K.NVAVPLYNR.F(58);K.FVDSTVVASVTIIDR.S(139);K.DASIQVVSAIR.A(110);R.SLASSLPGQTK.I(30)**

**Accession No.:** **ref|XP_021653602.1|**

**Mascot score:479 Sequence coverage %:46%**

**Calculated Mr:14713 Calculated pI:5.04**

**PFF Searched Score:**

**Matched peptide sequences: shown in Bold Red:**

**Spot No.:64**

**Protein name:** **tubulin alpha-3 chain [Hevea brasiliensis]**

**Peptide sequences: R.AVFVDLEPTVIDEVR.T(89);R.QLFHPEQLISGK.E(64);R.FDGAINVDVTEFQTNLVPYPR.I(100);K.CGINYQPPTVVPGGDLAR.V(90);R.AFVHWYVGEGMEEGEFSEAR.E(109)**

**Accession No.:** **ref|XP_021644460.1|**

**Mascot score:451 Sequence coverage %:19%**

**Calculated Mr:50244 Calculated pI:5.00**

**PFF Searched Score:**

**Matched peptide sequences: shown in Bold Red:**

**Spot No.:65**

**Protein name:** **proteasome subunit alpha type-6-like [Hevea brasiliensis]**

**Peptide sequences: R.HITIFSPEGR.L(65);K.LLDHTSVTHLFPITK.Y(83);K.YLGLLATGMTADAR.T(106);K.SQIYTQHAYMR.P(21);R.PLGVVAMVLGIDDEFGPR.L(95);K.CDPAGHYFGHK.A(70);K.ATEIEVGVVR.K(54);R.KENPDFR.M(29);R.MLSTEEIDEHLTAISER.D(82);R.MLSTEEIDEHLTAISERD.-(112)**

**Accession No.:** **ref|XP_021638924.1|**

**Mascot score:717 Sequence coverage %:46%**

**Calculated Mr:27662 Calculated pI:5.84**

**PFF Searched Score:**

**Matched peptide sequences: shown in Bold Red:**

**Spot No.:66**

**Protein name:** **rubber elongation factor protein-like [Hevea brasiliensis]**

**Peptide sequences: K.YLDFVQAATVYAR.A(104);K.SGPFKPGVNTVESR.F(100);K.SVVRPVYNK.F(35);R.RVDAYVTVLDR.I(77);R.VDAYVTVLDR.I(68)**

**Accession No.:** **ref|XP_021653600.1|**

**Mascot score:384 Sequence coverage %:26%**

**Calculated Mr:19612 Calculated pI:5.28**

**PFF Searched Score:**

**Matched peptide sequences: shown in Bold Red:**

**Spot No.:67**

**Protein name:** **probable mannitol dehydrogenase [Hevea brasiliensis]**

**Peptide sequences: K.QHLQPQESFGWAAR.D(107);K.VLYCGICHSDLHGIK.N(107);K.NDWGISIYPLLPGHEIVGIATEVGSNVEK.F(44);K.VGVGCMVGSCGSCNDCTNNLENYCPK.I(30);K.IIATYNGIDDDGAITYGGYSDFIIVDQHFVIR.Y(145);R.YPESLPLDAGAPLLCAGITVYSPLK.Y(67);K.LGVVGLGGLGHMAVK.F(74);K.EEATQHLGADSFLLSNDKNEMK.A(81);K.AAMGTLDGVIDTVSVFHAVLPIIGLLK.S(24);K.LVLLGLPDKPLELPVFPLLLGR.K(144);K.HNITADVEVISIDYVNK.A(153)**

**Accession No.:** **ref|XP_021672276.1|**

**Mascot score:974 Sequence coverage %:62%**

**Calculated Mr:42794 Calculated pI:6.27**

**PFF Searched Score:**

**Matched peptide sequences: shown in Bold Red:**

**Spot No.:68**

**Protein name:** **acetyl-CoA acetyltransferase, cytosolic 1 [Hevea brasiliensis]**

**Peptide sequences: R.ANVDPSLVQEVFFGNVLSANLGQAPAR.Q(57);R.EDQDNYAIHSFER.G(116);R.GIAAQDSGAFAWEIVPVEVSGGR.G(155);K.VNVHGGAVSLGHPLGCSGAR.I(63)**

**Accession No.:** **ref|XP_021656809.1|**

**Mascot score:391 Sequence coverage %:20%**

**Calculated Mr:41646 Calculated pI:6.01**

**PFF Searched Score:**

**Matched peptide sequences: shown in Bold Red:**

**Spot No.:69**

**Protein name:** **peroxiredoxin-2B-like [Hevea brasiliensis]**

**Peptide sequences: M.APVAVGDTLPDGTLAHFDEQDQLQQVSIHSLAAGTK.V(15);K.HVPGFIER.A(51);K.GVAEILCISVNDPFVMK.A(71);K.FLADGSATYTHALGLELDLNDK.G(92);R.RFALLVDDLK.V(66);R.FALLVDDLK.V(45);K.AANLEQGGEFTVSSVDEILK.A(123)**

**Accession No.:** **ref|XP_021641382.1|**

**Mascot score:461 Sequence coverage %:69%**

**Calculated Mr:17523 Calculated pI:5.49**

**PFF Searched Score:**

**Matched peptide sequences: shown in Bold Red:**

**Spot No.:70**

**Protein name:** **elongation factor 1-delta 1-like isoform X1 [Hevea brasiliensis]**

**Peptide sequences: K.APSSEYVNVSR.W(56);R.WYYHIDALLR.I(83);R.SIQMEGLLWGASK.L(33)**

**Accession No.:** **ref|XP_021690229.1|**

**Mascot score:172 Sequence coverage %:14%**

**Calculated Mr:25408 Calculated pI:4.47**

**PFF Searched Score:**

**Matched peptide sequences: shown in Bold Red:**

**Spot No.:71**

**Protein name:** **acetyl-CoA acetyltransferase, cytosolic 1 [Hevea brasiliensis]**

**Peptide sequences: R.EDQDNYAIHSFER.G(135);R.GIAAQDSGAFAWEIVPVEVSGGR.G(217);K.AVSNAGLDASQVDYYEINEAFAVVALANQK.L(143);K.VNVHGGAVSLGHPLGCSGAR.I(158);R.ILVTLLGVLR.Q(67)**

**Accession No.:** **ref|XP_021656809.1|**

**Mascot score:720 Sequence coverage %:23%**

**Calculated Mr:41646 Calculated pI:6.01**

**PFF Searched Score:**

**Matched peptide sequences: shown in Bold Red:**

**Spot No.:72**

**Protein name: rubber elongation factor protein-like**

**[Hevea brasiliensis]**

**Peptide sequences: K.SGPFKPGVNTVESR.F(109);R.RVDAYVTVLDR.I(80);R.VDAYVTVLDR.I(82)**

**Accession No.:** **ref|XP_021653600.1|**

**Mascot score:270 Sequence coverage %:14%**

**Calculated Mr:19612 Calculated pI:5.28**

**PFF Searched Score:**

**Matched peptide sequences: shown in Bold Red:**

**Spot No.: 73**

**Protein name: ubiquitin-conjugating enzyme E2 variant 1C isoform X2 [Hevea brasiliensis]**

**Peptide sequences: K.GIGDGTVSYGMDDGDDIYMR.S(126);R.SWTGTIIGPHNTVHEGR.I(99);K.KFGLLANWQR.E(44);K.FGLLANWQR.E(44);R.EYTMEDILTQLK.K(43);R.EYTMEDILTQLKK.E(56)**

**Accession No.:** **ref|XP_021636663.1|**

**Mascot score:412 Sequence coverage %:41%**

**Calculated Mr:16696 Calculated pI:6.20**

**PFF Searched Score:**

**Matched peptide sequences: shown in Bold Red:**

**Spot No.: 74**

**Protein name: carbonic anhydrase 2-like [Hevea brasiliensis]**

**Peptide sequences: K.LISEMPEHHLDPVQR.I(73);K.FLVFACSDSR.V(90);R.VSPSHVLDFQPGEAFMFR.N(75);R.NIANLVPAFNQLR.Y(127);R.YSGVGAVIEYAVK.Y(78);K.EAVNLSLVNIQSYPYVR.A(102)**

**Accession No.:** **ref|XP_021651451.1|**

**Mascot score:544 Sequence coverage %:33%**

**Calculated Mr:28840 Calculated pI:6.00**

**PFF Searched Score:**

**Matched peptide sequences: shown in Bold Red:**

**Spot No.: 75**

**Protein name: S-adenosylmethionine synthase 1-like**

**[Hevea brasiliensis]**

**Peptide sequences: K.VLVNIEQQSPDIAQGVHGHFTK.R(108);K.TQVTVEYYNDNGAMVPVR.V(76);R.VHTVLISTQHDETVTNDEIAADLK.E(105);K.TIFHLNPSGR.F(73);R.FVIGGPHGDAGLTGR.K(133);K.TAAYGHFGR.D(73)**

**Accession No.:** **ref|XP_021664714.1|**

**Mascot score:568 Sequence coverage %:24%**

**Calculated Mr:43654 Calculated pI:5.68**

**PFF Searched Score:**

**Matched peptide sequences: shown in Bold Red:**

**Spot No.: 76**

**Protein name: proteasome subunit alpha type-6-like**

**[Hevea brasiliensis]**

**Peptide sequences: R.HITIFSPEGR.L(65);K.LLDHTSVTHLFPITK.Y(83);K.YLGLLATGMTADAR.T(106);K.SQIYTQHAYMR.P(21);R.PLGVVAMVLGIDDEFGPR.L(95);K.CDPAGHYFGHK.A(70);K.ATEIEVGVVR.K(54);R.KENPDFR.M(29);R.MLSTEEIDEHLTAISER.D(82);R.MLSTEEIDEHLTAISERD.-(112)**

**Accession No.:** **ref|XP_021638924.1|**

**Mascot score:717 Sequence coverage %:46%**

**Calculated Mr:27662 Calculated pI:5.84**

**PFF Searched Score:**

**Matched peptide sequences: shown in Bold Red:**

**Spot No.: 77**

**Protein name: isoflavone reductase-like protein [Hevea brasiliensis]**

**Peptide sequences: K.ILIVGGTGYFGK.F(88);R.AGHPTFALAR.E(76);K.NLGVTILPGDLYDHESLVK.A(99);K.QVDVVISTVGALQVADQTK.I(81);K.IIAAINEAGNIKR.F(51);K.RFFPSEFGNDVDHVHAVEPAK.S(21);R.FFPSEFGNDVDHVHAVEPAK.S(100);R.AIEAAGIPYTYVPSNFFASLIIR.I(89);K.AVFNKEDDIATYTIK.A(115)**

**Accession No.:** **ref|XP_021644235.1|**

**Mascot score:720 Sequence coverage %:43%**

**Calculated Mr:33240 Calculated pI:5.50**

**PFF Searched Score:**

**Matched peptide sequences: shown in Bold Red:**

**Spot No.: 78**

**Protein name:** **elongation factor 2-like [Hevea brasiliensis]**

**Peptide sequences: R.NGNEYLINLIDSPGHVDFSSEVTAALR.I(47);R.ITDGALVVVDCVEGVCVQTETVLR.Q(64);R.CFLELQVDGEEAYQTFQR.V(63);K.AYLPVVESFGFSGTLR.A(40)**

**Accession No.:** **ref|XP_021676573.1|**

**Mascot score:215 Sequence coverage %:10%**

**Calculated Mr:94900 Calculated pI:5.80**

**PFF Searched Score:**

**Matched peptide sequences: shown in Bold Red:**

**Spot No.: 79**

**Protein name:** **universal stress protein PHOS32 isoform X1 [Hevea brasiliensis]**

**Peptide sequences: R.EVTLPSLIPVVPKPELER.E(66);R.GRDILIAIDHGPNSK.H(15)；K.HAFDWALIHLCR.L(74);R.GIVQSVLQGSVSEYCFHHCK.A**

**(73)**

**Accession No.:** **ref|XP_021659054.1|**

**Mascot score:228 Sequence coverage %:32%**

**Calculated Mr:22590 Calculated pI:5.91**

**PFF Searched Score:**

**Matched peptide sequences: shown in Bold Red:**

**Spot No.: 80**

**Protein name:** **triosephosphate isomerase, cytosolic**

**[Hevea brasiliensis]**

**Peptide sequences: K.FFVGGNWK.C(54);K.IVTTLNEAEVPSHDVVEVVVSPPFVFIPPVK.D(7);K.DLLRPDFQVAAQNCWVR.K(94);R.KGGAFTGEVSAEMLVNLSVPWVILGHSER.R(48);K.GGAFTGEVSAEMLVNLSVPWVILGHSER.R(21);R.SLLNESNEFVGDK.V(78);K.VIACIGETLEQR.E(101);K.VATPAQAQEVHLELR.K(133);K.VATPAQAQEVHLELRK.W(22);R.KWLHDNVCAEVAASTR.I(45);K.WLHDNVCAEVAASTR.I(128)**

**Accession No.:** **ref|XP_021646026.1|**

**Mascot score:729 Sequence coverage %:55%**

**Calculated Mr:27571 Calculated pI:5.90**

**PFF Searched Score:**

**Matched peptide sequences: shown in Bold Red:**

**Spot No.: 81**

**Protein name:** **protein-L-isoaspartate O-methyltransferase**

**1-like isoform X1 [Hevea brasiliensis]**

**Peptide sequences: R.QGWPEFAPYDAIHVGAAAPEIPHALVDQLKPGGR.M(94);R.MVIPVGNIFQDLK.V(35)**

**Accession No.:** **ref|XP_021681501.1|**

**Mascot score:129 Sequence coverage %:14%**

**Calculated Mr:35222 Calculated pI:9.28**

**PFF Searched Score:**

**Matched peptide sequences: shown in Bold Red:**

**Spot No.: 82**

**Protein name:** **rubber elongation factor protein-like**

**[Hevea brasiliensis]**

**Peptide sequences: K.YLDFVQAATVYAR.A(100);K.SGPFKPGVNTVESR.F(110);K.SVVRPVYNK.F(40);R.RVDAYVTVLDR.I(41);R.VDAYVTVLDR.I(41);R.ASIQAYSVAPGAAR.A(36)**

**Accession No.:** **ref|XP_021653600.1|**

**Mascot score:369 Sequence coverage %:34%**

**Calculated Mr:19612 Calculated pI:5.28**

**PFF Searched Score:**

**Matched peptide sequences: shown in Bold Red:**

**Spot No.: 83**

**Protein name:** **pro-hevein [Hevea brasiliensis]**

**Peptide sequences: K.YGWTAFCGPVGAHGQPSCGK.C(58);R.IVDQCSNGGLDLDVNVFR.Q(97);R.QLDTDGKGYER.G(35)**

**Accession No.:** **ref|XP_021650927.1|**

**Mascot score:190 Sequence coverage %:23%**

**Calculated Mr:23154 Calculated pI:5.89**

**PFF Searched Score:**

**Matched peptide sequences: shown in Bold Red:**

**Spot No.: 84**

**Protein name:** **translationally-controlled tumor protein homolog [Hevea brasiliensis]**

**Peptide sequences: K.VVDIVDTFR.L(39)**

**Accession No.:** **ref|XP_021634988.1|**

**Mascot score:39 Sequence coverage %:5%**

**Calculated Mr:19181 Calculated pI:4.43**

**PFF Searched Score:**

**Matched peptide sequences: shown in Bold Red:**

**Spot No.:85**

**Protein name:** **caffeic acid 3-O-methyltransferase-like**

**[Hevea brasiliensis]**

**Peptide sequences: K.SALELNVIDIISTAGNSGASLSAPEIAQR.I(216);K.NPEAPVLLDR.M(57);K.NGSGSAAPLLLLHHDEVFMK.S(32);K.SWYHLNEAILEGGTPFNR.A(119);R.AYGMNQFEYPGTDQR.F(87);R.AYGMNQFEYPGTDQR.F((Ions;K.WVLHDWNDDLCLK.L(72);R.TRQEFEALASK.S(67)**

**Accession No.:** **ref|XP_021638873.1|**

**Mascot score:659 Sequence coverage %:31%**

**Calculated Mr:40913 Calculated pI:5.54**

**PFF Searched Score:**

**Matched peptide sequences: shown in Bold Red:**

**Spot No.: 86**

**Protein name:** **small rubber particle protein-like isoform X1**

**[Hevea brasiliensis]**

**Peptide sequences: K.YLEFVQATTDNAVTALSNIYLYAK.D(126);K.DNSGPLKPGVETIEGVAK.T(152);R.AVDASFTTLQNIVPSVLK.Q(114);K.QLPTQACDTSVK.E(63)**

**Accession No.:** **ref|XP_** **021653603.1|**

**Mascot score:455 Sequence coverage %:61%**

**Calculated Mr:12769 Calculated pI:4.61**

**PFF Searched Score:**

**Matched peptide sequences: shown in Bold Red:**

**Spot No.: 87**

**Protein name:** **serine-threonine kinase**

**receptor-associated protein-like isoform X1 [Hevea brasiliensis]**

**Peptide sequences: K.GHHGPVHCVR.F(33);R.FSPGGESYASGSEDGTIR.I(85);R.IWQTGPANLEENDSLSR.N(77)**

**Accession No.:** **ref|XP_** **021659989.1|**

**Mascot score:195 Sequence coverage %:12%**

**Calculated Mr:38465 Calculated pI:5.74**

**PFF Searched Score:**

**Matched peptide sequences: shown in Bold Red:**

**Spot No.: 88**

**Protein name:** **patatin-like protein 2 [Hevea brasiliensis]**

**Peptide sequences: K.ITVLSIDGGGIR.G(82);K.LQDLDGPDAR.I(67);K.DIKDFYLENCPK.I(94);K.LLLPVIFSSDDAK.C(54);K.NIHTFELIDGGVAATNPTLLALTHIR.N(32);K.SLDCEDYYLR.I(70);R.IQDDTLTGEESSGHIATEENLQR.L(215)**

**Accession No.:** **ref|XP_** **021668042.1|**

**Mascot score:613 Sequence coverage %:24%**

**Calculated Mr:49142 Calculated pI:5.13**

**PFF Searched Score:**

**Matched peptide sequences: shown in Bold Red:**

**Spot No.: 89**

**Protein name:** **subtilisin-like protease SBT1.4 [Hevea brasiliensis]**

**Peptide sequences: R.EFPADVVLGDGR.I(92);R.IFGGVSLYSGEPLVDDKLPLVYAGDCGSK.Y(74);K.LSQFPTATIVFHGTVIGTSPPAPK.V(101);R.RVEFNIISGTSMSCPHVSGIAALLR.K(6);R.VEFNIISGTSMSCPHVSGIAALLR.K(28);K.AYPNWSPAAIK.S(36);R.IAVFVGGTATLDVCDR.K(133);R.IAVFVGGTATLDVCDRK.L(50);R.KLGSPGNLNYPSFSVVFEPNTNVVTHK.R(25);K.LGSPGNLNYPSFSVVFEPNTNVVTHK.R(93)**

**Accession No.:** **ref|XP_** **021638895.1|**

**Mascot score:637 Sequence coverage %:18%**

**Calculated Mr:82577 Calculated pI:6.66**

**PFF Searched Score:**

**Matched peptide sequences: shown in Bold Red:**

**Spot No.: 90**

**Protein name:** **proteasome subunit beta type-2-A-like**

**[Hevea brasiliensis]**

**Peptide sequences: K.NVALYQFR.N(56);R.NGIPLTTAAAANFTR.G(98);K.GAFGYGSYFSLSMMDR.H(66);R.LVVAPPNFLIK.I(58)**

**Accession No.:** **ref|XP_** **021689157.1|**

**Mascot score: 278 Sequence coverage %: 24%**

**Calculated Mr: 22489 Calculated pI: 6.06**

**PFF Searched Score:**

**Matched peptide sequences: shown in Bold Red:**

**Spot No.: 91**

**Protein name:** **pro-hevein [Hevea brasiliensis]**

**Peptide sequences: R.SKYGWTAFCGPVGAHGQPSCGK.C(4);K.YGWTAFCGPVGAHGQPSCGK.C(48);R.QLDTDGKGYER.G(87)**

**Accession No.:** **ref|XP_** **021650927.1|**

**Mascot score: 139 Sequence coverage %: 15%**

**Calculated Mr: 23154 Calculated pI: 5.89**

**PFF Searched Score:**

**Matched peptide sequences: shown in Bold Red:**

**Spot No.:92**

**Protein name: hsp70-Hsp90 organizing protein 3-like [Hevea brasiliensis]**

**Peptide sequences: K.GNAAFSAGDYAGAVR.H(101);R.HFTDAISLAPSNHVLYSNR.S(60);R.LGAAHLGLNQIQDAIAAYK.K(60);K.AMELDDEDISYLTNR.A(102);K.ALETYQEGLKHDPQNQELLDGVR.R(34);K.HDPQNQELLDGVR.R(17);K.LVNAGIVQIR.-(47)**

**Accession No.: ref|XP_021641666.1|**

**Mascot score:421 Sequence coverage %: 17**

**Calculated Mr:65314 Calculated pI:5.94**

**PFF Searched Score:**

**Matched peptide sequences: shown in Bold Red:**

**Spot No.: 93**

**Protein name: hsp70-Hsp90 organizing protein 3-like [Hevea brasiliensis]**

**Peptide sequences:**

**K.GNAAFSAGDYAGAVR.H(82);R.HFTDAISLAPSNHVLYSNR.S(83);R.LGAAHLGLNQIQDAIAAYK.K(15);R.APNAGEDMEVPEAEAPPPQPER.K(50);K.AMELDDEDISYLTNR.A(105);K.LVNAGIVQIR.-(45)**

**Accession No.:ref|XP_021641666.1|**

**Mascot score:379 Sequence coverage %: 17%**

**Calculated Mr:65314 Calculated pI:5.94**

**PFF Searched Score:**

**Matched peptide sequences: shown in Bold Red:**

**Spot No.: 94**

**Protein name: serpin-ZX-like [Hevea brasiliensis]**

**Peptide sequences: K.AVEVTNEVNAWAER.E(105);K.VGSESGFLEYHLPQQQVEVDDFR.I(128);K.SFIEVNEEGTEAAASSAGVVR.L(214);K.LNFVADHPFLFLIR.E(98)**

**Accession No.: ref|XP_021642770.1|**

**Mascot score: 546 Sequence coverage %: 18%**

**Calculated Mr:42827 Calculated pI:5.51**

**PFF Searched Score:**

**Matched peptide sequences: shown in Bold Red:**

**Spot No.: 95**

**Protein name: metacaspase-4-like [Hevea brasiliensis]**

**Peptide sequences: R.SAEPGDLLFVHYSGHGTR.L(139);R.LPAETGEDDDTGFDECIVPCDMNLITDDDFR.E(146);R.EFVDQVPHGCR.I(71);K.QTIQDAFESR.G(65);R.GVHLPSDLHHHHGHR.D(87);R.GVHLPSDLHHHHGHRDEEDFDNR.V(53);K.LRPTLFDMFGDDASPK.V(95);R.HGDGESGGGGFLGMVGSLAQEFLK.H(118);K.SSEAYGALSNAIQTIIAETDGAVTNQELVLK.A(81)**

**Accession No.: ref|XP_021692568.1|**

**Mascot score: 855 Sequence coverage %: 39%**

**Calculated Mr:46443 Calculated pI: 5.01**

**PFF Searched Score:**

**Matched peptide sequences: shown in Bold Red:**

**Spot No.: 96**

**Protein name: metacaspase-4-like [Hevea brasiliensis]**

**Peptide sequences: R.SAEPGDLLFVHYSGHGTR.L(121);R.LPAETGEDDDTGFDECIVPCDMNLITDDDFR.E(105);R.EFVDQVPHGCR.I(68);K.QTIQDAFESR.G(92);R.GVHLPSDLHHHHGHR.D(77);R.DEEDFDNRVVEEDYGDSGYVK.S(18);R.VVEEDYGDSGYVK.S(83);K.LRPTLFDMFGDDASPK.V(90);R.HGDGESGGGGFLGMVGSLAQEFLK.H(132)**

**Accession No.: ref|XP_021692568.1|**

**Mascot score: 786 Sequence coverage %:35%**

**Calculated Mr: 46443; Calculated pI: 5.01**

**PFF Searched Score:**

**Matched peptide sequences: shown in Bold Red:**

**Spot No.: 97**

**Protein name: acetyl-CoA acetyltransferase, cytosolic 1 [Hevea brasiliensis]**

**Peptide sequences: K.DGLWDVFNDVGMGSCAEICADNHSITR.E(28);R.EDQDNYAIHSFER.G(112);R.GIAAQDSGAFAWEIVPVEVSGGR.G(116);K.VNVHGGAVSLGHPLGCSGAR.I(41);R.ILVTLLGVLR.Q(44)**

**Accession No.: ref|XP_021656809.1|**

**Mascot score: 340 Sequence coverage %:23 %**

**Calculated Mr: 41646; Calculated pI: 6.01**

**PFF Searched Score:**

**Matched peptide sequences: shown in Bold Red:**

**Spot No.: 98**

**Protein name: putative lactoylglutathione lyase isoform X1 [Hevea brasiliensis]**

**Peptide sequences: K.FYTECFGMK.L(20);K.GGIISREPGPVK.G(44);K.DPDGYIFELIQR.G(87);R.GPTPEPLCQVMLR.V(8);R.GPTPEPLCQVMLR.V((Ions;K.GNAYAQVAIGTDDVYK.S(16);K.ITSFLDPDGWK.T(20)Accession No.: ref|XP_021646132.1|**

**Mascot score: 207 Sequence coverage %: 24%**

**Calculated Mr:33815 Calculated pI: 5.06**

**PFF Searched Score:**

**Matched peptide sequences: shown in Bold Red:**

**Spot No.: 99**

**Protein name: UPF0664 stress-induced protein C29B12.11c isoform X1 [Hevea brasiliensis]**

**Peptide sequences: K.GIIYLSNVR.M(52);K.EGGCGTFIPLFFNLISSVR.Q(106);R.QHNQQSIPR.T(60);R.TGPQMNPLVAAQTPVDEMMR.H(120);R.HAYVDPNDPTR.I(43);R.IFLQQPTPESELR.R(100);R.TYQLSPAER.S(52)**

**Accession No.: ref|XP_021679886.1|**

**Mascot score: 534 Sequence coverage %: 44%**

**Calculated Mr: 23052 Calculated pI: 6.51**

**PFF Searched Score:**

**Matched peptide sequences: shown in Bold Red:**

**Spot No.: 100**

**Protein name:enolase 1 [Hevea brasiliensis]**

**Peptide sequences:**

**R.AAVPSGASTGIYEALELR.D(146);K.SFVAEYPIVSIEDPFDQDDWAHYAK.L(120);R.AGWGVMASHR.S(34);R.SGETEDTFIADLSVGLATGQIK.T(116);R.IEEELGSEAVYAGANFR.K(159)**

**Accession No.:ref|XP_021678789.1|**

**Mascot score:575 Sequence coverage %: 20%**

**Calculated Mr:48029 Calculated pI:5.57**

**PFF Searched Score:**

**Matched peptide sequences: shown in Bold Red:**

**Spot No.: 101**

**Protein name: hydroxymethylglutaryl-CoA synthase-like [Hevea brasiliensis]**

**Peptide sequences:**

**K.NVGILAVDIYFPPTFVQQEALEAHDGASK.G(55);R.YGLVVCTDSAVYAEGPAR.P(106);R.GSHMSHAYDFYKPNLASEYPVVDGK.L(30);K.LSQTCYLMALDSCYK.H(53);K.QFSISDAEYFVFHSPYNK.L(82);R.LVFNDFVR.N(64);K.LAPFSNLSGDESYQNR.D(117);K.DCSLLASGTYYLTEVDSLYR.R(131)**

**Accession No.:ref|XP_021666594.1|**

**Mascot score:637 Sequence coverage %:32%**

**Calculated Mr:51638 Calculated pI: 6.33**

**PFF Searched Score:**

**Matched peptide sequences: shown in Bold Red:**

**Spot No.: 102**

**Protein name:ankyrin repeat domain-containing protein 2A-like isoform X1 [Hevea brasiliensis]**

**Peptide sequences:**

**R.YWNDKEVLQK.L(52);K.NALASGANKDEEDSEGR.T(70);R.TALHFACGYGEVK.C(87);K.NTALHYAAGYGR.K(77)**

**Accession No.:ref|XP_021646829.1|**

**Mascot score:286 Sequence coverage %:14%**

**Calculated Mr:37639 Calculated pI:4.50**

**PFF Searched Score:**

**Matched peptide sequences: shown in Bold Red:**

**Spot No.: 103**

**Protein name: non-specific phospholipase C3-like [Hevea brasiliensis]**

**Peptide sequences: K.TVVVLVQENR.S(43);R.SFDHMLGWLK.T(52);K.ELAMNFAICDR.W(57);R.WFASIPSLTQPNR.L(73);K.TIFESMDESGFTFGIYYQHPPSTLFYR.N(37);K.HCEEGKLPNYVVIEPR.Y(118);K.LPNYVVIEPR.Y(82);R.YFDLLNLPANDDHPSHNVSEGQK.F(78);R.DAWAGTFESVLTR.T(123);R.TDCPVTLPEPVR.L(59)**

**Accession No.: ref|XP_021673245.1|**

**Mascot score: 720 Sequence coverage %:26 %**

**Calculated Mr: 58852; Calculated pI: 5.17**

**PFF Searched Score:**

**Matched peptide sequences: shown in Bold Red:**

**Spot No.: 104**

**Protein name: UTP--glucose-1-phosphate uridylyltransferase [Hevea brasiliensis]**

**Peptide sequences: K.YGCNVPLLLMNSFNTHDDTQK.I(66);K.SNVEIHTFNQSQYPR.L(114);K.EYVFIANSDNLGAIVDLK.I(108);K.VQLLEIAQVPDKHVNEFK.S(73);K.VLQLETAAGAAIR.F(106)**

**Accession No.: ref|XP_021643510.1|**

**Mascot score: 466 Sequence coverage %: 18 %**

**Calculated Mr: 51500 Calculated pI: 6.30**

**PFF Searched Score:**

**Matched peptide sequences: shown in Bold Red:**

**Spot No.: 105**

**Protein name: beta-glucosidase 42 isoform X1 [Hevea brasiliensis]**

**Peptide sequences: R.SDFPPNFLFGVATSAYQIEGGCR.E(145);K.YFAIYADTCFASFGDR.V(74);R.NSLDFIGLNHYSSR.F(98);K.WEDGEPIGER.A(71);R.AASEWLYVCPWGLR.K(74);K.SSAYWFMR.F(41)**

**Accession No.: ref|XP_021636834.1|**

**Mascot score: 503 Sequence coverage %: 17%**

**Calculated Mr: 56484 Calculated pI: 5.49**

**PFF Searched Score:**

**Matched peptide sequences: shown in Bold Red:**

**Spot No.: 106**

**Protein name: peptidyl-prolyl cis-trans isomerase FKBP62-like [Hevea brasiliensis]**

**Peptide sequences:**

**K.EGEGWDTPENGDEVEVHYTGTLLDGTQFDSSR.D(90)；K.KGHFCPALSR.A(26)；K.GHFCPALSR.A(46)；K.VLLTVKPQYGFGEK.G(52)；K.EGEGYERPNDGAVVK.L(45)；K.GHDNEDEVFEFKTDEEQVIEGLDR.A(133)；K.YIEYDSSFGEEEKK.Q(69)；R.RAQAYIQLADLDVAEFDVK.K (9)**

**Accession No.:ref|XP_021660834.1|**

**Mascot score:471 Sequence coverage %:22%**

**Calculated Mr:62805 Calculated pI:5.36**

**PFF Searched Score:**

**Matched peptide sequences: shown in Bold Red:**

**Spot No.: 107**

**Protein name: leucine aminopeptidase 1-like [Hevea brasiliensis]**

**Peptide sequences: R.VGLVGLGQSASTTLAFR.N(134);K.LNTASAIASGTVLGIYEDNR.Y(154);K.LNTASAIASGTVLGIYEDNRYK.S(116);R.GVEVHFIVAACENMISGTGMRPGDIVTASNGK.T(2);K.VQWMHIDLAGPVWNEK.K(93)**

**Accession No.: ref|XP_021672059.1|**

**Mascot score: 499 Sequence coverage %: 14%**

**Calculated Mr:61645 Calculated pI:6.30**

**PFF Searched Score:**

**Matched peptide sequences: shown in Bold Red:**

**Spot No.:108**

**Protein name: heat shock cognate protein 80 [Hevea brasiliensis]**

**Peptide sequences:**

**K.HNDDEQYVWESQAGGSFTVTR.D(175)；K.ITLFLKEDQLEYLEER.R(95)；K.HFSVEGQLEFK.A(64)；K.RAPFDLFDTR.K(51)；R.APFDLFDTR.K(51)；K.GIVDSEDLPLNISR.E(85)；K.KTMEINPENPIMDELR.K(29)**

**Accession No.:ref|XP_021660158.1|**

**Mascot score:548 Sequence coverage %:12%**

**Calculated Mr:80388 Calculated pI:4.97**

**PFF Searched Score:**

**Matched peptide sequences: shown in Bold Red:**

**Spot No.: 109**

**Protein name: proteasome subunit alpha type-4-like [Hevea brasiliensis]**

**Peptide sequences:**

**R.TTIFSPEGR.L(55)；K.EGVVLVGEK.K(25)；K.IDDHVACAVAGIMSDANILINMAR.V（132)；R.YTYAYQEPMPVEQLVQSLCDTK.Q(52)； K.AAAIGANNQAAQSMLK.Q(20)；K.DDITREEAVQLALK.V (10)；K.TMDSTSLTSDKLELAEVFVLPSGEVK.Y(80)**

**Accession No.:ref|XP_021690224.1|**

**Mascot score:374 Sequence coverage %:48%**

**Calculated Mr:27519 Calculated pI:5.96**

**PFF Searched Score:**

**Matched peptide sequences: shown in Bold Red:**

**Spot No.: 110**

**Protein name: metacaspase-4-like [Hevea brasiliensis]**

**Peptide sequences:**

**R.SAEPGDLLFVHYSGHGTR.L(24);R.EFVDQVPHGCR.I(45);K.QTIQDAFESR.G(33);R.VVEEDYGDSGYVK.S(10);K.LRPTLFDMFGDDASPK.V（25）**

**Accession No.:ref|XP_021692568.1|**

**Mascot score:137 Sequence coverage %:16%**

**Calculated Mr:46443 Calculated pI:5.01**

**PFF Searched Score:**

**Matched peptide sequences: shown in Bold Red:**

**Spot No.:111**

**Protein name: hydroxymethylglutaryl-CoA synthase-like [Hevea brasiliensis]**

**Peptide sequences:**

**K.NVGILAVDIYFPPTFVQQEALEAHDGASK.G(83);R.YGLVVCTDSAVYAEGPAR.P(80);R.GSHMSHAYDFYKPNLASEYPVVDGK.L(32);K.QFSISDAEYFVFHSPYNK.L(81);R.LVFNDFVR.N(60);K.LAPFSNLSGDESYQNR.D(134);K.VSQQVAKPLYDAK.V(71);K.ARHELPPEK.F(31)**

**Accession No.:ref|XP_021666594.1|**

**Mascot score:572 Sequence coverage %:29%**

**Calculated Mr:51638 Calculated pI:6.33**

**PFF Searched Score:**

**Matched peptide sequences: shown in Bold Red:**

**Spot No.:112**

**Protein name:leucine aminopeptidase 1-like [Hevea brasiliensis]**

**Peptide sequences:**

**K.RVGLVGLGQSASTTLAFR.N(9);R.VGLVGLGQSASTTLAFR.N(165);K.LNTASAIASGTVLGIYEDNR.Y(113);K.LNTASAIASGTVLGIYEDNRYK.S(83);K.YAEDVSSAVIFGR.E(132);K.MGSYLGVAAASANPPHFIHLCYKPPSGPVK.A(41);K.GLTFDSGGYNIK.T(78);R.GVEVHFIVAACENMISGTGMRPGDIVTASNGK.T(3);R.MPLEESYWESMK.S(29);K.VQWMHIDLAGPVWNEK.K(68)**

**Accession No.:ref|XP_021672059.1|**

**Mascot score:721 Sequence coverage %:26%**

**Calculated Mr:61645 Calculated pI:630**

**PFF Searched Score:**

**Matched peptide sequences: shown in Bold Red:**

**Spot No.: 113**

**Protein name:fructose-bisphosphate aldolase 3, chloroplastic-like [Hevea brasiliensis]**

**Peptide sequences:**

**R.LASIGLDNTEVNR.Q(34);K.FVDCLRDENIVPGIK.V(56);K.GLVPLPGSNNESWCQGLDGLASR.S(139);R.TVVSIPCGPSALAVR.E(56);R.YAAISQDNGLVPIVEPEILLDGDHGIER.T(73);K.TWQGRPENVEAAQK.A(46)**

**Accession No.:ref|XP_021636981.1|**

**Mascot score:404 Sequence coverage %:27%**

**Calculated Mr:43250 Calculated pI:8.09**

**PFF Searched Score:**

**Matched peptide sequences: shown in Bold Red:**

**Spot No.: 114**

**Protein name: rubber elongation factor protein-like [Hevea brasiliensis]**

**Peptide sequences: K.YLDFVQAATVYAR.A(115);K.SVVRPVYNK.F(47);R.RVDAYVTVLDR.I(63);R.VDAYVTVLDR.I(35);R.ASIQAYSVAPGAAR.A(36);R.AVASYLPLHTK.R(39)**

**Accession No.: ref|XP_021653600.1|**

**Mascot score: 335 Sequence coverage %: 33%**

**Calculated Mr: 19612; Calculated pI:5.28**

**PFF Searched Score:**

**Matched peptide sequences: shown in Bold Red:**

**Spot No.: 115**

**Protein name: proteasome subunit beta type-3-A isoform X1 [Hevea brasiliensis]**

**Peptide sequences: K.NCFAIASDR.R(52);K.NCFAIASDRR.L(24);R.RLGVQLQTIATDFQR.I(4);R.LGVQLQTIATDFQR.I(125);R.LFIGLSGLATDAQTLYQR.L(169);R.DMKPETFASLVSALLYEK.R(44);R.FGPYFCQPVIAGLSDEDKPFICTMDSIGAK.E(27);R.DCLSGWGGHVYVVTPTEVK.E(53)**

**Accession No.: ref|XP_021667325.1|**

**Mascot score: 497 Sequence coverage %:53%**

**Calculated Mr: 23143 Calculated pI:5.33**

**PFF Searched Score:**

**Matched peptide sequences: shown in Bold Red:**

**Spot No.: 116**

**Protein name: hydroxymethylglutaryl-CoA synthase-like [Hevea brasiliensis]**

**Peptide sequences: K.NVGILAVDIYFPPTFVQQEALEAHDGASK.G(128);K.TFLMQIFEK.F(Ions;R.YGLVVCTDSAVYAEGPAR.P(96);R.GSHMSHAYDFYKPNLASEYPVVDGK.L(Ions;K.QFSISDAEYFVFHSPYNK.L(69);R.LVFNDFVR.N(13);K.LAPFSNLSGDESYQNR.D(128);K.VSQQVAKPLYDAK.V(46);K.ARHELPPEK.F(11);K.DCSLLASGTYYLTEVDSLYR.R(133)**

**Accession No.: ref|XP_021666594.1|**

**Mascot score: 724 Sequence coverage %:35 %**

**Calculated Mr:51638 Calculated pI:6.33**

**PFF Searched Score:**

**Matched peptide sequences: shown in Bold Red:**

**Spot No.: 117**

**Protein name: elicitor-responsive protein 3-like isoform X1 [Hevea brasiliensis]**

**Peptide sequences: M.PLGTVEVLLVGAK.G(103);K.GLENTDFLNGVDPYVVLACR.T(86);K.FSFEVSDGDTELTLK.I(160);K.GEITVGLTFTPEVEMDNVGVDGYDFR.L(Ions**

**Accession No.: ref|XP_021663495.1|**

**Mascot score: 446 Sequence coverage %: 52%**

**Calculated Mr: 15249; Calculated pI:4.06**

**PFF Searched Score:**

**Matched peptide sequences: shown in Bold Red:**

**Spot No.: 118**

**Protein name: adenosine kinase 2 [Hevea brasiliensis]**

**Peptide sequences: K.YNVEYIAGGATQNSIR.V(85);K.QAGVNVHYYEDETAPTGTCAVCVVGGER.S(79);K.RPENWALVEK.A(21);K.VLPYMDYVFGNETEAR.T(101);R.AGCYAANVIIQR.S(54)**

**Accession No.: ref|XP_021684501.1|**

**Mascot score: 339 Sequence coverage %: 24%**

**Calculated Mr:37935 Calculated pI:5.37**

**PFF Searched Score:**

**Matched peptide sequences: shown in Bold Red:**

**Spot No.: 119**

**Protein name: GDSL esterase/lipase At2g38180-like [Hevea brasiliensis]**

**Peptide sequences: R.VIFLGVPPVNEDMIR.E(118);R.EFFGNDTGR.T(69);R.IYSEACLEVCREEEIR.I(69);R.IPGIDLWNAIQR.K(99);K.TCFVDGIHLSAEGSEVVVEEILNVLR.E(224)**

**Accession No.: ref|XP_021639632.1|**

**Mascot score: 578 Sequence coverage %: 32%**

**Calculated Mr: 27072; Calculated pI: 5.19**

**PFF Searched Score:**

**Matched peptide sequences: shown in Bold Red:**

**Spot No.: 120**

**Protein name: elongation factor 2-like [Hevea brasiliensis]**

**Peptide sequences: K.FTAEELRR.I(17);R.NGNEYLINLIDSPGHVDFSSEVTAALR.I(79);R.ITDGALVVVDCVEGVCVQTETVLR.Q(92);R.CFLELQVDGEEAYQTFQR.V(77);K.YRVENLYEGPLDDPYATAIR.N(31);R.PMEDGLAEAIDEGR.I(49);K.ILSEEFGWDKDLAK.K(24);R.GHVFEELQRPGTPLYNIK.A(43);K.AYLPVVESFGFSGTLR.A(64)**

**Accession No.: ref|XP_021676573.1|**

**Mascot score: 476 Sequence coverage %: 18%**

**Calculated Mr: 94900; Calculated pI: 5.80**

**PFF Searched Score:**

**Matched peptide sequences: shown in Bold Red:**

**Spot No.: 121**

**Protein name: actin-depolymerizing factor 2-like [Hevea brasiliensis]**

**Peptide sequences: K.LGEPTQSYEDFTASLPADECR.Y(128);R.YAVYDFDFVTEENCQK.S(105);R.IFFIAWSPDTSR.V(92);R.ELDGIQVELQATDPTEMGLDVFK.S(58)**

**Accession No.: ref|XP_021689182.1|**

**Mascot score: 383 Sequence coverage %: 51%**

**Calculated Mr:16220 Calculated pI:5.92**

**PFF Searched Score:**

**Matched peptide sequences: shown in Bold Red:**

**Spot No.: 122**

**Protein name: actin-7 [Hevea brasiliensis]**

**Peptide sequences: R.AVFPSIVGRPR.H(17);R.VAPEEHPVLLTEAPLNPK.A(54);K.NYELPDGQVITIGAER.F(69);K.GEYDESGPSIVHR.K(56)**

**Accession No.: ref|XP_021666817.1|**

**Mascot score: 196 Sequence coverage %: 15%**

**Calculated Mr:41897 Calculated pI:5.31**

**PFF Searched Score:**

**Matched peptide sequences: shown in Bold Red:**

**Spot No.: 123**

**Protein name: uncharacterized protein LOC110671066 [Hevea brasiliensis]**

**Peptide sequences: R.FHVLVCAPDSEK.S(83);K.SAVSHSITWR.H(42);R.RVYIEGATAFAVSGTPADCASLGVSK.A(43);K.NQTYPSGCFLNIDLPTNVANHK.G(113);K.MLSTMTMDTNSAVQTETGASNVAQEHMWFR.R(72)**

**Accession No.: ref|XP_021689132.1|**

**Mascot score: 353 Sequence coverage %: 32%**

**Calculated Mr: 33735; Calculated pI:5.22**

**PFF Searched Score:**

**Matched peptide sequences: shown in Bold Red:**

**Spot No.: 124**

**Protein name: probable fructokinase-4 [Hevea brasiliensis]**

**Peptide sequences: K.APGGAPANVAIAVAR.L(105);R.NPSADMLLKPEELNLELIR.S(136);K.VFHYGSISLIVEPCR.S(102);K.DAGALLSYDPNLR.L(96);R.LPLWPSPEEAR.E(84);K.VSDVELEFLTGSDKIDDADALSLWHPNLK.L(71)**

**Accession No.: ref|XP_021688622.1|**

**Mascot score: 594 Sequence coverage %: 30%**

**Calculated Mr:35625 Calculated pI:5.22**

**PFF Searched Score:**

**Matched peptide sequences: shown in Bold Red:**

**Spot No.: 125**

**Protein name: pro-hevein [Hevea brasiliensis]**

**Peptide sequences: K.YGWTAFCGPVGAHGQPSCGK.C(3);R.IVDQCSNGGLDLDVNVFR.Q(123);R.QLDTDGKGYER.G(6)**

**Accession No.: ref|XP_021650927.1|**

**Mascot score: 132 Sequence coverage %: 23%**

**Calculated Mr:23154 Calculated pI:5.89**

**PFF Searched Score:**

**Matched peptide sequences: shown in Bold Red:**

**Spot No.: 126**

**Protein name: putative lactoylglutathione lyase isoform X1 [Hevea brasiliensis]**

**Peptide sequences: R.RFLHVVYR.V(14);R.FLHVVYR.V(44);K.FYTECFGMK.L(37);K.DPDGYIFELIQR.G(114);R.GPTPEPLCQVMLR.V(78);K.ITSFLDPDGWK.T(80)**

**Accession No.: ref|XP_021646132.1|**

**Mascot score: 368 Sequence coverage %: 17%**

**Calculated Mr:33815 Calculated pI:5.06**

**PFF Searched Score:**

**Matched peptide sequences: shown in Bold Red:**

**Spot No.: 127**

**Protein name: universal stress protein PHOS32-like [Hevea brasiliensis]**

**Peptide sequences: K.IGVAVDLSDESAYAVR.W(110);R.LGSVSDYCVHHCVCPVVVVR.Y(38)**

**Accession No.: ref|XP_021648769.1|**

**Mascot score: 148 Sequence coverage %: 13%**

**Calculated Mr:28561 Calculated pI:5.78**

**PFF Searched Score:**

**Matched peptide sequences: shown in Bold Red:**

**Spot No.: 128**

**Protein name:pro-hevein [Hevea brasiliensis]**

**Peptide sequences:**

**K.YGWTAFCGPVGAHGQPSCGK.C(5);R.QLDTDGKGYER.G(69)**

**Accession No.:ref|XP_021650927.1|**

**Mascot score:73 Sequence coverage %:14%**

**Calculated Mr:23154 Calculated pI:5.89**

**PFF Searched Score:**

**Matched peptide sequences: shown in Bold Red:**

**Spot No.: 129**

**Protein name:eukaryotic translation initiation factor 5A-like [Hevea brasiliensis]**

**Peptide sequences:**

**K.TYPQQAGTIR.K(63);K.CHFVGIDIFNAK.K(74);K.KLEDIVPSSHNCDVPHVTR.T(121);R.TDYQLIDISEDGFVSLLTENGNTK.D(65);K.DDLRLPTDENLLSQIK.D(78);R.LPTDENLLSQIK.D(37);K.DLVVTVMSSMGEEQICALK.D(46)**

**Accession No.:ref|XP_021645712.1|**

**Mascot score: 483 Sequence coverage %: 62**

**Calculated Mr: 17689 Calculated pI:5.60**

**PFF Searched Score:**

**Matched peptide sequences: shown in Bold Red:**

**Spot No.: 130**

**Protein name:guanosine nucleotide diphosphate dissociation inhibitor 2 [Hevea brasiliensis]**

**Peptide sequences:**

**K.YGLDDNTVDFIGHSLALHR.D(130);R.DDQYLDEPALDTVMR.M(108);R.FQGGSPYIYPLYGLGELPQAFAR.L(140);K.VVCDPSYLPNK.V(51);R.AIAIMSHPIPNTNDSHSVQVILPQK.Q(26);R.RSDMYLFCCSYTHNVAPK.G(12)**

**Accession No.:ref|XP_021673535.1|**

**Mascot score:467 Sequence coverage %:25%**

**Calculated Mr:50142 Calculated pI:5.54**

**PFF Searched Score:**

**Matched peptide sequences: shown in Bold Red:**

**Spot No.: 131**

**Protein name:superoxide dismutase [Cu-Zn]-like isoform X1 [Hevea brasiliensis]**

**Peptide sequences:**

**K.DHGGPEDENR.H(31);K.HIPLSGPHSIAGR.S(27);R.SVVFHEGRDDLGK.G(27)**

**Accession No.:ref|XP_021669573.1|**

**Mascot score:86 Sequence coverage %:22%**

**Calculated Mr:16423 Calculated pI:5.73**

**PFF Searched Score:**

**Matched peptide sequences: shown in Bold Red:**

**Spot No.: 132**

**Protein name:V-type proton ATPase catalytic subunit A [Hevea brasiliensis]**

**Peptide sequences:**

**R.LTTFEDSEKESEYGYVR.K(108);R.VGHDNLIGEIIR.L(100);R.LEGDSATIQVYEETAGLMVNDPVLR.T(174);K.QFTMLQTWPVR.T(61);K.LAADTPLLTGQR.V(75);R.EASIYTGITIAEYFR.D(152);R.LAEMPADSGYPAYLAAR.L(92);K.YSSALESFYDQFDPDFINIR.T(112);R.EVLQREDDLNEIVQLVGK.D(66)**

**Accession No.:ref|XP_021681620.1|**

**Mascot score:941 Sequence coverage %:23%**

**Calculated Mr:68933 Calculated pI:5.34**

**PFF Searched Score:**

**Matched peptide sequences: shown in Bold Red:**

**Spot No.: 133**

**Protein name:non-specific phospholipase C3-like [Hevea brasiliensis]**

**Peptide sequences:**

**K.TVVVLVQENR.S(47);K.ELAMNFAICDR.W(49);R.WFASIPSLTQPNR.L(64);K.HCEEGKLPNYVVIEPR.Y(105);K.LPNYVVIEPR.Y(62);R.YFDLLNLPANDDHPSHNVSEGQK.F(46);R.DAWAGTFESVLTR.T(103)**

**Accession No.:ref|XP_021673245.1|**

**Mascot score:476 Sequence coverage %:16%**

**Calculated Mr:58852 Calculated pI:5.17**

**PFF Searched Score:**

**Matched peptide sequences: shown in Bold Red:**

**Spot No.:134**

**Protein name:26S proteasome regulatory subunit 6B homolog [Hevea brasiliensis]**

**Peptide sequences:**

**R.EAVELPLTHHELYK.Q(4);K.AVANHTTAAFIR.V(49);K.ENAPAIIFIDEVDAIATAR.F(5);R.ADTLDPALLRPGR.L(7);K.KPDTDFEFYK.-(20)**

**Accession No.:ref|XP_021666129.1|**

**Mascot score:85 Sequence coverage %:16%**

**Calculated Mr:47304 Calculated pI:5.42**

**PFF Searched Score:**

**Matched peptide sequences: shown in Bold Red:**

**Spot No.:135**

**Protein name:GDSL esterase/lipase-like [Hevea brasiliensis]**

**Peptide sequences:**

**K.YLPSYHHPYGTTFFDYPTGR.F(50);R.TVVDFVAENVSLPR.I(48);K.AVYLISFGADDYLNYEIPSEASR.E(70);R.EQLESIVDVVLGNISDR.I(100);R.IKELYDFGAR.K(62);K.ELYDFGAR.K(5);K.EIIDAPGEHGFK.Y(85)**

**Accession No.:ref|XP_021664438.1|**

**Mascot score:419 Sequence coverage %:26%**

**Calculated Mr:41480 Calculated pI:4.60**

**PFF Searched Score:**

**Matched peptide sequences: shown in Bold Red:**

**Spot No.:136**

**Protein name:proline iminopeptidase isoform X1 [Hevea brasiliensis]**

**Peptide sequences:**

**R.IILFDQR.G(50);R.STPHACLVENTSWDLIADIEK.L(137);K.EIDWFYEGGAAAIYPDAWEPFR.D(171);R.DLIPENER.G(51);K.RGDDDDFSLAFAR.I(76);R.GDDDDFSLAFAR.I(81);K.GFFPSDSFLLDNVHK.I(104)**

**Accession No.:ref|XP_021656697.1|**

**Mascot score:670 Sequence coverage %:21%**

**Calculated Mr:44578 Calculated pI:5.80**

**PFF Searched Score:**

**Matched peptide sequences: shown in Bold Red:**

**Spot No.:137**

**Protein name:tubulin alpha-3 chain [Hevea brasiliensis]**

**Peptide sequences:**

**R.AVFVDLEPTVIDEVR.T(89);R.QLFHPEQLISGK.E(64);R.FDGAINVDVTEFQTNLVPYPR.I(100);K.CGINYQPPTVVPGGDLAR.V(90);R.AFVHWYVGEGMEEGEFSEAR.E(109)**

**Accession No.:ref|XP_021644460.1|**

**Mascot score:451 Sequence coverage %:19%**

**Calculated Mr:50244 Calculated pI:5.00**

**PFF Searched Score:**

**Matched peptide sequences: shown in Bold Red:**

**Spot No.:138**

**Protein name:pro-hevein [Hevea brasiliensis]**

**Peptide sequences:**

**K.YGWTAFCGPVGAHGQPSCGK.C(5);R.QLDTDGKGYER.G(69)**

**Accession No.:ref|XP_021650927.1|**

**Mascot score:73 Sequence coverage %:14%**

**Calculated Mr:23154 Calculated pI:5.89**

**PFF Searched Score:**

**Matched peptide sequences: shown in Bold Red:**

**Spot No.:139**

**Protein name: leucine aminopeptidase 1-like [Hevea brasiliensis]**

**Peptide sequences:**

**R.VGLVGLGQSASTTLAFR.N(134);K.LNTASAIASGTVLGIYEDNR.Y(154);K.LNTASAIASGTVLGIYEDNRYK.S(116);R.GVEVHFIVAACENMISGTGMRPGDIVTASNGK.T(2);K.VQWMHIDLAGPVWNEK.K(93)**

**Accession No.:ref|XP_021672059.1|**

**Mascot score:499 Sequence coverage %: 14%**

**Calculated Mr:61645 Calculated pI:6.30**

**PFF Searched Score:**

**Matched peptide sequences: shown in Bold Red:**

**Spot No.:140**

**Protein name:rubber elongation factor protein [Hevea brasiliensis]**

**Peptide sequences:**

**K.YLGFVQDAATYAVTTFSNVYLFAK.D(170);K.DKSGPLQPGVDIIEGPVK.N(118);K.NVAVPLYNR.F(63);K.FVDSTVVASVTIIDR.S(132);K.DASIQVVSAIR.A(72)**

**Accession No.:ref|XP_021653602.1|**

**Mascot score:554 Sequence coverage %:55%**

**Calculated Mr:14713 Calculated pI:5.04**

**PFF Searched Score:**

**Matched peptide sequences: shown in Bold Red:**

**Spot No.:141**

**Protein name:translationally-controlled tumor protein homolog [Hevea brasiliensis]**

**Peptide sequences:**

**K.EIHNGILWEVEGK.W(38);K.WVVQGAVDVDIGANPSAEGADEDEGVDDQAVK.V(51);K.VVDIVDTFR.L(68);R.LQEQPAFDKK.Q(36);K.LSDLQFFVGESMHDDGSLVFAYYR.E(121);R.EGATDPTFLYFAYALK.E(62)**

**Accession No.:ref|XP_021637200.1|**

**Mascot score:376 Sequence coverage %:61%**

**Calculated Mr:19156 Calculated pI:4.47**

**PFF Searched Score:**

**Matched peptide sequences: shown in Bold Red:**

**Spot No.:142**

**Protein name:proteasome subunit alpha type-3-like [Hevea brasiliensis]**

**Peptide sequences:**

**R.HSGMAVAGLAADGR.Q(86);K.SEATNYESVYGEPIPVNELAGR.V(126);R.DGPQLYMIEPSGISYR.Y(93);K.AFELEMSWVCDESKR.L(107)**

**Accession No.:ref|XP_021674040.1|**

**Mascot score:412 Sequence coverage %:26%**

**Calculated Mr:27595 Calculated pI:6.11**

**PFF Searched Score:**

**Matched peptide sequences: shown in Bold Red:**

**Spot No.:143**

**Protein name:non-specific phospholipase C3-like [Hevea brasiliensis]**

**Peptide sequences:**

**K.TVVVLVQENR.S(28);K.ELAMNFAICDR.W(55);R.WFASIPSLTQPNR.L(46);K.HCEEGKLPNYVVIEPR.Y(47);R.DAWAGTFESVLTR.T(94)**

**Accession No.:ref|XP_021673245.1|**

**Mascot score: 270 Sequence coverage %:12%**

**Calculated Mr:58852 Calculated pI:5.17**

**PFF Searched Score:**

**Matched peptide sequences: shown in Bold Red:**

**Spot No.:144**

**Protein name:malate dehydrogenase [Hevea brasiliensis]**

**Peptide sequences:**

**R.ELIKDDEWLNAEFITTVQQR.G(68);K.IVQGLHIDEFSR.K(69)**

**Accession No.:ref|XP_021640500.1|**

**Mascot score:137 Sequence coverage %:9%**

**Calculated Mr:36173 Calculated pI:6.19**

**PFF Searched Score:**

**Matched peptide sequences: shown in Bold Red:**
